# Preliminary investigations of plasma lipidome and selenium levels in adults with treated hypothyroidism and in healthy individuals without selenium deficiency

**DOI:** 10.1038/s41598-024-80862-9

**Published:** 2024-11-25

**Authors:** Anna Błażewicz, Julia Wojnicka, Andreas M. Grabrucker, Piotr Sosnowski, Alicja Trzpil, Anna Kozub-Pędrak, Klaudia Szałaj, Agnieszka Szmagara, Ewelina Grywalska, Katarzyna Skórzyńska-Dziduszko

**Affiliations:** 1 Department of Pathobiochemistry and Interdisciplinary Applications of Ion Chromatography, Chair of Biomedical Sciences, Medical University of Lublin, 1 Chodźki Street, 20-093 Lublin, Poland; 2 Department of Biological Sciences, University of Limerick, Limerick, V94 T9PX Ireland; 3 Bernal Institute, University of Limerick, Limerick, V94 T9PX Ireland; 4 Health Research Institute (HRI), University of Limerick, Limerick, V94 T9PX Ireland; 5 Department of Bioanalytics, Medical University of Lublin, ul. Jaczewskiego 8b, 20-090 Lublin, Poland; 6 Faculty of Medicine, Institute of Biological Sciences, Department of Chemistry, The John Paul II Catholic University of Lublin, Konstantynow 1J, 20-708 Lublin, Poland; 7 Department of Experimental Immunology, Medical University of Lublin, 4a Chodźki Street, 20-093 Lublin, Poland; 8 Department of Human Physiology, Medical University of Lublin, Radziwillowska 11 Str., 20-080 Lublin, Poland

**Keywords:** Human plasma lipidome, Selenium, Hypothyroidism, Biomarkers, Medical research

## Abstract

The present preliminary study aimed to provide a targeted lipidomic analysis of Hashimoto (HT) and non-HT patients with well-controlled hypothyroidism as well as in healthy adults, and is the first to demonstrate the association of several components of the human lipidome with hypothyroidism in relation to the total plasma selenium content. All the patients and age-, sex-, and BMI-matched healthy controls met the very strict qualification criteria. Se levels were analyzed by ICP-MS, and lipidome studies were conducted using TQ-LC/MS. The 40 acylcarnitines, 90 glycerophospholipids, and 15 sphingomyelins were identified and quantified. PCaaC26:0 and PCaaC40:1 were negatively correlated with Se concentrations. Other lipids that were negatively correlated with Se concentrations but did not present significant differences between the three groups in the Kruskal–Wallis ANOVA test were PCaaC32:0, PCaeC30:0, PCaeC36:5, SMC18:0, and SM C18:1. In the multiple linear regression analyses, Se levels showed negative relationship, whereas different phosphatidylcholines: PCaaC24:0, PCaaC26:0, PCaeC30:1, PCaeC34:0, PCaeC36:4, PCaeC42:0 were positively associated with the presence of (H). Different lipidome components were identified in healthy and hypothyroid patients regardless of the cause of that condition. Studies on larger populations are needed to determine cause-and-effect relations and the potential mechanisms underlying these associations.

## Introduction

Lipids are diverse and multifunctional molecules integral to cellular structure, signaling, and function. Studying lipid species in biological samples, including human body fluids or tissues, provides valuable insights into biology, physiology, disease mechanisms, and potential ways for therapeutic intervention. Research on human lipidome, i.e., the total lipid content within a cell, organ, or biological system, can be used in risk assessment model development, disease diagnosis, and its monitoring^1^. Understanding how lipids function and how to control their activity also offers the potential to develop novel treatments for controlling numerous diseases. The involvement of lipids in metabolic pathways and their role in the pathology of diseases related to the endocrine system is increasingly understood^2^. Hormones modulate every pathway in lipoprotein metabolism, influence the expression of lipoprotein receptors, the production of apolipoproteins, the activity of plasma lipoprotein-modifying enzymes, and the blood concentrations of substrates for triglycerides (TGs) synthesis, such as fatty acids and glucose. Therefore, it is anticipated that endocrine disruption, including hypothyroidism, alters the lipid profile^3^. Hypothyroidism is a common endocrine disorder resulting from a deficiency of thyroid hormones (TH). Worldwide, iodine deficiency is the most common cause of hypothyroidism; however, in Poland and other areas of adequate iodine intake, chronic autoimmune thyroiditis—the Hashimoto’s disease also known as the Hashimoto thyroiditis is the primary reason for hypothyroidism^4,5^.

Although it has been suggested that overt hypothyroidism results in elevated levels of low-density lipoprotein (LDL)- cholesterol, high-density lipoprotein (HDL)- cholesterol, and triglycerides (TGs) in serum^6^, the relationship between subclinical hypothyroidism (SCH) and blood lipid metabolism remains controversial, and the definite association between SCH and routinely measured blood lipid levels such as TC (total cholesterol), LDL-C, and HDL-C has not been confirmed^7^. In contrary, another study revealed that the total cholesterol level in SCH was positively correlated with the level of TSH^8^.

Recently, several studies have shown that thyroid hormones can affect incident dyslipidemia in a general euthyroid population^9,10^. Low but normal FT3 was associated with high dyslipidemia risk, especially for elevated TC and LDL-C, and normal TSH had a weak positive effect on the incidence of reduced HDL-C^9^. In another study, positive and significant relationships between TSH level and TG level as well as between FT4 level and TC, LDL-C, and HDL-C cholesterol levels were demonstrated^10^. Since levothyroxine (LT4) replacement therapy reduces levels of TSH, it is well known that the treatment could also reduce TC and LDL-C in hypothyroidic patients, including those with mild SCH^11,12^.

The current standard of care for hypothyroidism is levothyroxine (LT4) monotherapy to reduce levels of TSH within its reference range. Once a patient’s thyroid hormones are in the normal range, the Endocrine Society clinical practice guideline being the first to focus on lipid management in patients with endocrine disorders, recommends re-evaluating the lipid profile^12^. Since thyroid hormones modulate a number of pathways involved in lipid metabolism, it is doubtful whether a basic blood lipid profile assessment in hypothyroidic patients with biochemical euthyroidism (i.e. thyroid hormones and TSH within the normal range) brings adequate information on the complexity of lipids activity and turnover. Using a modern lipidomic approach, we intended to find out to what extent and which specific lipid molecules differ quantitatively in healthy and hypothyroid adults with biochemical euthyroidism and without any comorbidities. To the best of our knowledge, human lipidome alterations have not been studied in such patients with well-controlled hypothyroidism (i.e. stable TSH levels within the normal range on the appropriate dose of LT4 replacement therapy).

Furthermore, other factors may mediate the interplay between the action of thyroid hormones and lipid compounds, considering that thyroid hormones play an important role in maintaining energy homeostasis in the body. The essential trace element selenium (Se) is known to influence such a balance^13^. There are many reports on the disruption of the physiological levels of Se in the body that adversely affect the functioning of cells and tissues, which can lead to the development of hypothyroidism^14^. Selenium is required for the antioxidant function and the metabolism of thyroid hormones, and its intake has been associated with autoimmune disorders^15^. Duntas et al.^16^ investigated the effect of selenium treatment in the form of selenomethionine in patients with autoimmune thyroiditis by affecting the levels of TPOAb and TgAb after 3 and 6 months. In the supplement group TPOAb levels decreased by 46% after 3 months and by 46% after 6 months compared to a decrease of only 21% and 27%, respectively, at 3 and 6 months in the group treated with thyroxine. Nevertheless, there was no statistically significant statistically significant difference in TPOAb levels or in the levels of TSH, free T4 and T3 between the two groups. There were no significant changes of antibodies against thyroglobulin levels between these groups. In turn, another randomized double-blind placebo-controlled trial investigating whether adding selenium to standard LT4 treatment in Hashimoto’s thyroid patients can lead to improved quality of life and reduced autoimmune disease activity did not justify the routine use of selenium supplementation in patients with Hashimoto’s disease, as selenium supplementation for 12 months, compared to placebo, did not improve patients’ quality of life, LT4 dose or FT3I / FT4 ratio^17^. Despite some inconsistencies presented in the literature regarding either low or high Se status in hypothyroid patients, there is clear evidence of a close relationship between this element and thyroid health and function^18,19^. Recently, integrated analysis of miRNAs and the proteome in SCH mice highlighted an interesting associations between some miRNAs and proteins, including selenium-binding protein 2, which contributes to a better understanding of lipid metabolism disorders in subclinical hypothyroidism^20^. Although there are some data indicating that no strong correlation exists between serum selenium levels and lipid profile in humans without any pathology of the thyroid gland^21^, the associations between Se in plasma and the plasma lipidome in patients with hypothyroidism remain unknown.

Our cohort study aimed to reveal which lipid species are altered in hypothyroidic patients with biochemical euthyroidism who present normal serum lipid profiles (LDL-cholesterol, HDL-cholesterol, total cholesterol, and triglycerides). Furthermore, we intended to investigate whether selenium levels in these patients are associated with the content of individual lipid molecules and whether the possible Se-lipidome association is modified by thyroid status. This may provide a theoretical basis for the better understanding and control of thyroid dysfunctions and related lipid imbalances, as well as it may help to reveal more about human metabolism.

## Methods

### Patients and control group

The cross-sectional cohort study was initiated in 2021. The study protocol received ethical approval from the Medical University of Lublin Bioethics Committee (KE-0254/7/2021). Informed consent was obtained in writing from each participant. The study was conducted in accordance with the principles of the Declaration of Helsinki. The patients included in the study had a diagnosis of either Hashimoto’s disease or non-autoimmune hypothyroidism (Hypo-non-Hashimoto), according to endocrinological assessment. This study investigated plasma lipidome in 24 HT patients with autoimmune hypothyroidism, 11 non-HT patients with hypothyroidism without autoimmune thyroiditis, and 6 age-, sex-, and body mass index (BMI)-matched healthy controls. Subjects were recruited from a specialized thyroid outpatient unit with higher frequencies of thyroid disorders than in the general population. The control group consisted of individuals with normal thyroid function. Thyroid-stimulating hormone (TSH), free T4 (fT4), free T3 (fT3), serum lipid profile, fasting glucose, and fasting insulin were measured. Thyroid peroxidase antibody (TPOAb) and antithyroglobulin antibody (TgAb) were assessed to confirm autoimmune thyroiditis in patients with Hashimoto’s disease.

Patients with either Hashimoto’s disease or non-autoimmune hypothyroidism were receiving thyroid hormone replacement therapy with levothyroxine (LT4) in tablets (doses of LT4 are shown in Tables 1 and 2).

**Table 1:** Clinical profile of participating subjects with Hashimoto’s disease.

Variable	Hashimoto’s disease								
	Valid N	Mean	Median	Minimum	Maximum	Lower quartile	Upper quartile	Quartile range	Std. dev
Age (years)	24	33.500	29.500	22.000	59.000	23.000	43.000	20.00	11.527
BMI (kg/m^2^)	24	23.865	23.420	17.710	35.010	20.660	26.730	6.070	4.346
LT4 dose (µg)	24	75.682	75.000	12.500	137.000	56.250	100.000	43.750	30.362
Hypothyroidism (years)	24	5.750	5.000	1.000	19.000	2.500	8.000	5.500	4.406
TSH (uIU/mL)	24	1.989	1.910	0.870	3.795	1.550	2.380	0.830	0.722
fT4 (ng/dL)	24	1.180	1.100	0.810	1.710	0.970	1.410	0.440	0.269
fT3 (pg/mL)	24	2.983	2.985	2.040	4.650	2.445	3.415	0.970	0.6360
Total cholesterol (mg/dL)	24	173.741	175.500	98.000	245.000	158.500	188.500	30.000	31.957
HDL cholesterol (mg/dL)	24	62.715	63.500	39.000	98.000	55.200	68.500	13.300	14.062
LDL cholesterol (mg/dL)	24	92.081	92.600	49.000	147.200	67.900	105.800	37.900	25.080
Triglycerides (mg/dL)	24	89.226	96.000	36.000	142.000	61.500	114.000	52.500	31.058
AIP	24	0.135	0.149	-0.142	0.430	0.004	0.289	0.280	0.171
Fasting glucose (mg/dL)	24	88.271	89.000	77.000	98.000	83.500	92.000	8.500	5.651
Fasting insulin (mIU/L)	24	8.313	9.300	3.000	12.000	5.960	10.300	4.340	2.583
HOMA-IR	24	1.821	1.962	0.578	2.667	1.354	2.347	0.993	0.591
Se (µg/L)	24	89.993	93.805	53.660	122.200	76.300	101.700	25.400	20.251
Lipid species									
lysoPC a C14:0	24	2.111	1.970	1.120	4.110	1.575	2.310	0.735	0.755
lysoPC a C16:0	24	126.458	127.000	88.800	174.000	113.500	137.000	23.500	21.833
lysoPC a C16:1	24	2.940	2.885	1.760	4.810	2.430	3.305	0.875	0.708
lysoPC a C17:0	24	2.083	2.095	0.853	3.360	1.775	2.435	0.660	0.557
lysoPC a C18:0	24	39.338	39.150	19.700	53.800	34.700	46.500	11.800	8.537
lysoPC a C18:1	24	29.242	30.000	13.200	39.500	25.250	34.600	9.350	6.854
lysoPC a C18:2	24	40.404	39.600	12.000	66.400	30.850	51.250	20.400	13.928
lysoPC a C20:3	24	2.545	2.565	1.440	3.560	2.200	2.940	0.740	0.594
lysoPC a C20:4	24	7.774	7.425	3.790	11.900	6.350	9.050	2.700	2.173
lysoPC a C24:0	24	0.214	0.201	0.086	0.576	0.156	0.246	0.090	0.099
lysoPC a C26:0	24	0.332	0.233	0.134	1.330	0.188	0.381	0.193	0.255
lysoPC a C26:1	24	0.216	0.153	0.088	0.886	0.123	0.236	0.112	0.171
lysoPC a C28:0	24	0.299	0.247	0.144	0.955	0.200	0.332	0.132	0.169
lysoPC a C28:1	24	0.431	0.403	0.186	1.220	0.296	0.503	0.207	0.217
PC aa C24:0	24	50.577	50.692	0.083	101.000	0.138	101.000	100.862	51.507
PC aa C26:0	24	80.237	101.000	0.841	101.000	101.000	101.000	0.000	41.345
PC aa C28:1	24	2.727	2.845	1.480	3.870	2.170	3.180	1.0100	0.755
PC aa C30:0	24	3.775	3.680	1.860	10.300	2.820	4.220	1.40	1.801
PC aa C30:2	24	101.000	101.000	101.000	101.000	101.000	101.000	0.000	0.000
PC aa C32:0	24	12.525	12.150	7.700	25.800	10.200	14.050	3.850	3.644
PC aa C32:1	24	13.434	11.550	5.460	43.600	8.610	15.800	7.190	7.953
PC aa C32:2	24	2.854	2.735	0.966	5.900	1.960	3.425	1.465	1.236
PC aa C32:3	24	0.353	0.366	0.203	0.534	0.286	0.413	0.127	0.085
PC aa C34:1	24	207.667	198.000	116.000	457.000	171.500	220.500	49.000	64.836
PC aa C34:2	24	426.958	416.000	258.000	668.000	348.500	484.000	135.500	95.442
PC aa C34:3	24	13.174	12.450	7.310	23.600	11.000	14.750	3.7500	3.901
PC aa C34:4	24	1.428	1.330	0.489	3.650	0.978	1.555	0.5770	0.726
PC aa C36:0	24	1.087	1.075	0.198	2.060	0.765	1.450	0.6850	0.519
PC aa C36:1	24	39.442	38.200	24.200	82.200	31.700	44.050	12.350	12.060
PC aa C36:2	24	212.833	215.500	127.000	280.000	169.000	256.500	87.500	48.007
PC aa C36:3	24	105.892	100.650	59.700	167.000	87.600	111.500	23.900	27.680
PC aa C36:4	24	168.125	158.500	87.000	316.000	141.500	174.500	33.000	49.408
PC aa C36:5	24	18.322	16.950	7.750	59.200	13.150	21.500	8.350	10.006
PC aa C36:6	24	0.664	0.679	0.242	1.730	0.500	0.772	0.2720	0.2911
PC aa C38:0	24	2.366	2.200	1.540	3.750	1.825	2.780	0.955	0.693
PC aa C38:1	24	0.511	0.526	0.195	1.010	0.343	0.641	0.2980	0.205
PC aa C38:3	24	31.929	30.500	22.000	60.800	26.900	34.300	7.40	8.316
PC aa C38:4	24	78.104	74.350	49.200	146.000	64.650	86.600	21.950	20.336
PC aa C38:5	24	36.438	34.900	20.800	85.400	32.750	37.950	5.200	11.530
PC aa C38:6	24	64.133	59.150	29.900	140.000	46.800	71.200	24.400	23.620
PC aa C40:1	24	96.807	101.000	0.371	101.000	101.000	101.000	0.000	20.540
PC aa C40:2	24	0.173	0.174	0.096	0.264	0.148	0.192	0.044	0.039
PC aa C40:3	24	0.324	0.306	0.237	0.470	0.259	0.364	0.104	0.075
PC aa C40:4	24	1.893	1.795	0.955	4.520	1.480	2.105	0.625	0.6561
PC aa C40:5	24	5.324	4.900	3.380	16.900	4.255	5.445	1.190	2.5760
PC aa C40:6	24	18.375	16.400	10.800	52.000	13.500	19.550	6.050	8.589
PC aa C42:0	24	0.365	0.319	0.201	0.648	0.297	0.434	0.136	0.116
PC aa C42:1	24	0.172	0.166	0.090	0.289	0.121	0.216	0.094	0.055
PC aa C42:2	24	0.138	0.135	0.075	0.195	0.108	0.169	0.061	0.035
PC aa C42:4	24	0.105	0.105	0.063	0.167	0.095	0.119	0.025	0.025
PC aa C42:5	24	0.207	0.202	0.112	0.403	0.165	0.232	0.066	0.060
PC aa C42:6	24	0.236	0.227	0.146	0.444	0.201	0.260	0.059	0.063
PC ae C30:0	24	0.367	0.363	0.157	0.548	0.305	0.425	0.120	0.095
PC ae C30:1	24	71.574	101.000	0.016	101.000	0.243	101.000	100.757	46.842
PC ae C30:2	24	0.084	0.079	0.043	0.173	0.065	0.100	0.0355	0.029
PC ae C32:1	24	2.493	2.365	1.520	3.930	2.040	2.905	0.8650	0.597
PC ae C32:2	24	0.600	0.602	0.346	0.840	0.477	0.719	0.242	0.147
PC ae C34:0	24	1.086	1.030	0.523	2.070	0.909	1.175	0.260	0.321
PC ae C34:1	24	8.873	8.605	5.700	11.500	7.950	10.300	2.350	1.628
PC ae C34:2	24	11.637	12.050	5.840	17.300	8.650	13.850	5.200	3.243
PC ae C34:3	24	7.165	7.200	4.600	10.700	5.530	8.165	2.635	1.835
PC ae C36:0	24	0.561	0.583	0.380	0.806	0.451	0.664	0.213	0.122
PC ae C36:1	24	5.924	5.930	3.440	8.360	4.750	6.710	1.960	1.252
PC ae C36:2	24	11.070	11.200	6.530	16.100	8.875	13.100	4.225	2.584
PC ae C36:3	24	6.450	6.365	3.850	9.770	4.825	7.630	2.805	1.792
PC ae C36:4	24	16.777	17.000	7.240	28.800	12.300	20.300	8.000	5.047
PC ae C36:5	24	10.198	9.750	5.410	16.300	7.950	12.900	4.950	2.868
PC ae C38:0	24	1.422	1.370	0.854	2.370	1.240	1.615	0.375	0.323
PC ae C38:1	24	25.376	0.185	0.015	101.000	0.111	50.698	50.587	44.600
PC ae C38:2	24	1.011	1.000	0.611	1.580	0.870	1.120	0.250	0.2441
PC ae C38:3	24	2.414	2.390	1.500	3.340	2.160	2.740	0.580	0.474
PC ae C38:4	24	9.718	9.270	6.020	16.300	8.355	11.050	2.695	2.396
PC ae C38:5	24	14.589	15.050	9.940	22.500	11.250	17.500	6.250	3.424
PC ae C38:6	24	6.080	6.005	2.990	9.270	4.760	7.265	2.5050	1.673
PC ae C40:1	24	0.831	0.815	0.558	1.340	0.703	0.930	0.22	0.185
PC ae C40:2	24	1.242	1.215	0.632	1.810	0.933	1.455	0.522	0.33
PC ae C40:3	24	0.619	0.587	0.403	0.889	0.553	0.670	0.116	0.121
PC ae C40:4	24	1.454	1.385	0.859	2.540	1.300	1.555	0.255	0.364
PC ae C40:5	24	2.186	2.095	1.430	3.360	1.925	2.370	0.445	0.463
PC ae C40:6	24	2.983	2.880	1.880	4.860	2.435	3.375	0.940	0.760
PC ae C42:0	24	75.868	101.000	0.396	101.000	50.782	101.000	50.218	44.466
PC ae C42:1	24	0.185	0.184	0.118	0.272	0.158	0.214	0.055	0.040
PC ae C42:2	24	0.337	0.314	0.235	0.570	0.285	0.374	0.089	0.080
PC ae C42:3	24	0.479	0.473	0.222	0.691	0.413	0.578	0.165	0.116
PC ae C42:4	24	8.947	0.549	0.346	101.000	0.506	0.710	0.204	28.352
PC ae C42:5	24	1.492	1.365	0.874	2.580	1.255	1.695	0.440	0.407
PC ae C44:3	24	0.066	0.065	0.035	0.103	0.053	0.077	0.024	0.018
PC ae C44:4	24	0.244	0.220	0.114	0.421	0.208	0.282	0.074	0.072
PC ae C44:5	24	1.300	1.230	0.694	2.380	1.090	1.435	0.345	0.376
PC ae C44:6	24	0.789	0.748	0.423	1.460	0.658	0.863	0.205	0.251
SM (OH) C14:1	24	4.746	4.575	2.720	7.070	3.725	5.975	2.250	1.308
SM (OH) C16:1	24	2.448	2.410	1.410	3.790	2.050	2.830	0.780	0.661
SM (OH) C22:1	24	7.929	7.840	5.000	11.000	6.690	9.035	2.345	1.741
SM (OH) C22:2	24	7.336	7.105	5.040	10.400	5.825	9.050	3.225	1.647
SM (OH) C24:1	24	0.740	0.721	0.450	1.110	0.605	0.836	0.2305	0.178
SM C16:0	24	97.396	94.750	64.900	130.000	84.250	112.500	28.25	17.964
SM C16:1	24	12.260	11.750	8.090	17.100	9.635	14.650	5.015	2.7261
SM C18:0	24	17.160	16.600	9.540	24.300	14.250	20.550	6.300	4.2460
SM C18:1	24	7.770	7.410	4.550	11.300	6.305	9.125	2.820	1.8818
SM C20:2	24	0.205	0.178	0.069	0.376	0.140	0.266	0.126	0.0894
SM C22:3	24	101.000	101.000	101.000	101.000	101.000	101.000	0.000	0.0000
SM C24:0	24	12.099	11.750	8.220	20.500	10.450	13.600	3.150	2.591
SM C24:1	24	39.229	39.950	26.000	61.600	33.150	44.550	11.400	7.856
SM C26:0	24	0.088	0.089	0.016	0.141	0.065	0.109	0.044	0.029
SM C26:1	24	0.234	0.233	0.105	0.373	0.178	0.280	0.102	0.067

Abbreviations of lipid species: Lysophosphatidylcholine with acyl residue (lysoPC a); Phosphatidylcholine with diacyl residue (PC aa); Phosphatidylcholine with acyl-alkyl residue (PC ae); Hydroxysphingomyelin with acyl residue (SM (OH)); Sphingomyelin with acyl residue (SM).

**Table 2:** Clinical profile of participating subjects with non-autoimmune hypothyroidism.

Variable	Non-autoimmune hypothyroidism (Hypo-no-Hashimoto)								
	Valid N	Mean	Median	Minimum	Maximum	Lower quartile	Upper quartile	Quartile range	Std. dev
Age (years)	11	29.273	24.000	21.000	43.000	22.000	38.000	16.000	8.343
BMI (kg/m^2^)	11	23.072	24.510	19.000	25.820	19.840	25.350	5.510	2.711
LT4 dose (µg)	11	47.074	50.000	25.000	75.000	28.570	57.140	28.570	16.290
Hypothyroidism (years)	11	4.000	2.000	1.000	12.000	1.000	8.000	7.000	4.242
TSH (uIU/mL)	11	1.663	1.680	0.704	2.674	1.140	2.230	1.090	0.590
fT4 (ng/dL)	11	2.071	1.320	0.850	9.930	1.190	1.520	0.330	2.614
fT3 (pg/mL)	11	3.175	2.980	2.410	4.050	2.730	3.730	1.000	0.583
Total cholesterol (mg/dL)	11	164.273	164.000	134.000	202.000	152.000	174.000	22.000	18.810
HDL cholesterol (mg/dL)	11	58.182	57.000	48.000	89.000	49.000	59.000	10.000	12.015
LDL cholesterol (mg/dL)	11	90.900	92.500	69.600	134.000	73.000	97.200	24.200	18.397
Triglycerides (mg/dL)	11	87.000	89.000	36.000	123.000	70.000	105.000	35.000	24.157
AIP	11	0.163	0.225	-0.393	0.400	0.089	0.265	0.176	0.205
Fasting glucose (mg/dL)	11	88.091	86.000	80.000	101.000	82.000	93.000	11.000	6.876
Fasting insulin (mIU/L)	11	8.120	7.600	4.600	10.920	6.400	9.830	3.430	2.058
HOMA-IR	11	1.769	1.558	1.034	2.508	1.422	2.230	0.807	0.489
Se (µg/L)	11	84.593	82.500	45.550	126.667	57.917	100.250	42.333	27.545
Lipid species									
lysoPC a C14:0	11	1.782	1.540	1.020	3.180	1.290	2.170	0.880	0.705
lysoPC a C16:0	11	110.064	106.000	79.300	149.000	98.200	122.000	23.800	18.653
lysoPC a C16:1	11	2.618	2.450	1.780	4.920	1.960	3.020	1.060	0.886
lysoPC a C17:0	11	1.894	1.850	1.420	2.640	1.500	2.260	0.760	0.411
lysoPC a C18:0	11	33.227	35.800	18.500	44.900	25.800	39.300	13.500	8.256
lysoPC a C18:1	11	24.682	23.900	17.400	31.600	18.900	30.200	11.300	5.224
lysoPC a C18:2	11	31.736	31.600	13.900	50.000	28.000	38.200	10.200	10.055
lysoPC a C20:3	11	2.028	1.830	1.050	4.280	1.390	2.520	1.130	0.883
lysoPC a C20:4	11	6.275	5.950	3.850	8.740	4.840	8.230	3.390	1.833
lysoPC a C24:0	11	0.258	0.216	0.127	0.493	0.202	0.321	0.119	0.106
lysoPC a C26:0	11	0.414	0.404	0.128	0.822	0.212	0.653	0.441	0.235
lysoPC a C26:1	11	0.239	0.177	0.097	0.541	0.139	0.392	0.253	0.140
lysoPC a C28:0	11	0.323	0.295	0.166	0.652	0.218	0.364	0.146	0.141
lysoPC a C28:1	11	0.500	0.476	0.202	0.915	0.327	0.618	0.291	0.216
PC aa C24:0	11	36.811	0.142	0.076	101.000	0.124	101.000	100.876	50.890
PC aa C26:0	11	46.407	1.110	0.849	101.000	0.874	101.000	100.126	52.268
PC aa C28:1	11	2.611	2.600	1.990	4.120	2.060	2.970	0.910	0.619
PC aa C30:0	11	3.177	3.320	1.860	4.610	2.200	4.400	2.200	1.064
PC aa C30:2	11	101.000	101.000	101.000	101.000	101.000	101.000	0.000	0.000
PC aa C32:0	11	11.278	10.800	8.960	16.900	9.260	12.700	3.440	2.386
PC aa C32:1	11	11.562	11.200	4.920	23.800	7.860	13.300	5.440	5.405
PC aa C32:2	11	2.454	2.100	0.370	5.470	1.660	3.690	2.030	1.413
PC aa C32:3	11	0.330	0.305	0.203	0.553	0.238	0.442	0.204	0.115
PC aa C34:1	11	186.182	176.000	140.000	246.000	161.000	215.000	54.000	35.439
PC aa C34:2	11	383.091	375.000	321.000	485.000	356.000	404.000	48.000	43.532
PC aa C34:3	11	12.804	11.400	5.440	24.300	10.200	15.600	5.400	5.112
PC aa C34:4	11	1.179	0.947	0.343	2.410	0.818	1.750	0.932	0.650
PC aa C36:0	11	0.950	0.810	0.125	2.100	0.721	1.340	0.619	0.535
PC aa C36:1	11	34.600	35.200	27.200	43.500	28.100	38.900	10.800	5.939
PC aa C36:2	11	190.364	183.000	158.000	255.000	171.000	201.000	30.000	27.990
PC aa C36:3	11	96.245	88.700	66.400	124.000	85.800	111.000	25.200	16.881
PC aa C36:4	11	144.482	151.000	99.300	184.000	111.000	168.000	57.000	29.848
PC aa C36:5	11	18.834	16.800	9.370	33.900	13.200	24.100	10.900	7.238
PC aa C36:6	11	0.632	0.505	0.306	1.210	0.416	0.842	0.426	0.270
PC aa C38:0	11	2.074	2.000	1.460	3.140	1.670	2.230	0.560	0.557
PC aa C38:1	11	9.762	0.622	0.169	101.000	0.364	1.010	0.646	30.261
PC aa C38:3	11	28.336	25.500	18.200	41.500	23.100	37.600	14.500	7.390
PC aa C38:4	11	68.173	61.400	44.100	104.000	55.600	75.700	20.100	17.763
PC aa C38:5	11	34.509	34.200	23.700	51.000	26.500	40.800	14.300	7.814
PC aa C38:6	11	67.091	61.500	45.800	123.000	50.800	72.800	22.000	21.921
PC aa C40:1	11	91.854	101.000	0.391	101.000	101.000	101.000	0.000	30.334
PC aa C40:2	11	0.171	0.170	0.096	0.257	0.144	0.193	0.049	0.042
PC aa C40:3	11	0.319	0.309	0.224	0.448	0.270	0.348	0.078	0.063
PC aa C40:4	11	1.583	1.530	0.967	2.670	1.100	1.880	0.780	0.520
PC aa C40:5	11	4.902	4.220	2.800	9.340	3.810	5.760	1.950	1.809
PC aa C40:6	11	18.282	17.200	12.800	29.200	13.000	23.200	10.200	5.473
PC aa C42:0	11	0.364	0.328	0.252	0.703	0.306	0.379	0.073	0.119
PC aa C42:1	11	0.179	0.172	0.104	0.311	0.161	0.183	0.022	0.053
PC aa C42:2	11	0.139	0.131	0.106	0.198	0.112	0.165	0.053	0.028
PC aa C42:4	11	0.096	0.096	0.057	0.138	0.066	0.130	0.064	0.030
PC aa C42:5	11	0.214	0.206	0.148	0.366	0.155	0.247	0.092	0.068
PC aa C42:6	11	0.230	0.225	0.113	0.334	0.172	0.291	0.119	0.066
PC ae C30:0	11	0.334	0.311	0.207	0.475	0.251	0.412	0.161	0.092
PC ae C30:1	11	55.114	101.000	0.021	101.000	0.062	101.000	100.938	52.718
PC ae C30:2	11	0.084	0.085	0.051	0.118	0.067	0.099	0.032	0.018
PC ae C32:1	11	2.131	2.230	1.500	2.950	1.580	2.410	0.830	0.466
PC ae C32:2	11	0.532	0.507	0.452	0.737	0.454	0.592	0.138	0.090
PC ae C34:0	11	0.923	0.799	0.652	1.430	0.700	1.100	0.400	0.252
PC ae C34:1	11	8.244	7.730	5.710	11.400	6.590	9.860	3.270	1.917
PC ae C34:2	11	9.494	8.900	5.520	12.900	8.160	11.800	3.640	2.236
PC ae C34:3	11	6.322	5.490	4.210	11.200	4.960	7.350	2.390	1.988
PC ae C36:0	11	0.540	0.483	0.387	0.951	0.411	0.586	0.175	0.161
PC ae C36:1	11	5.577	5.680	3.850	7.570	4.220	7.010	2.790	1.350
PC ae C36:2	11	9.991	10.100	7.160	12.600	7.780	11.400	3.620	1.963
PC ae C36:3	11	5.029	4.740	3.070	6.630	4.350	6.210	1.860	1.082
PC ae C36:4	11	12.189	11.700	7.530	16.900	9.620	14.500	4.880	3.017
PC ae C36:5	11	8.080	7.530	5.170	14.600	5.990	9.470	3.480	2.718
PC ae C38:0	11	1.466	1.510	0.983	2.080	1.220	1.780	0.560	0.347
PC ae C38:1	11	36.861	0.276	0.068	101.000	0.171	101.000	100.829	50.850
PC ae C38:2	11	0.879	0.916	0.434	1.280	0.667	1.090	0.423	0.260
PC ae C38:3	11	2.224	2.300	1.060	3.010	1.910	2.690	0.780	0.583
PC ae C38:4	11	8.051	7.650	4.480	10.800	6.450	10.100	3.650	2.042
PC ae C38:5	11	11.657	11.500	8.610	16.700	9.390	13.500	4.110	2.553
PC ae C38:6	11	5.242	5.000	4.030	7.840	4.090	5.710	1.620	1.290
PC ae C40:1	11	0.779	0.717	0.550	1.160	0.670	0.890	0.220	0.173
PC ae C40:2	11	1.203	1.190	0.819	1.640	0.924	1.450	0.526	0.274
PC ae C40:3	11	0.588	0.575	0.412	0.781	0.460	0.730	0.270	0.129
PC ae C40:4	11	1.280	1.310	0.794	1.800	1.070	1.490	0.420	0.299
PC ae C40:5	11	1.946	1.880	1.390	3.060	1.480	2.340	0.860	0.517
PC ae C40:6	11	2.882	2.790	1.990	4.460	2.460	2.800	0.340	0.759
PC ae C42:0	11	55.321	101.000	0.388	101.000	0.473	101.000	100.527	52.480
PC ae C42:1	11	0.185	0.184	0.121	0.263	0.150	0.219	0.069	0.044
PC ae C42:2	11	0.336	0.321	0.246	0.558	0.290	0.340	0.050	0.082
PC ae C42:3	11	0.464	0.447	0.314	0.722	0.416	0.471	0.055	0.106
PC ae C42:4	11	0.526	0.517	0.335	0.724	0.443	0.604	0.161	0.123
PC ae C42:5	11	1.403	1.420	0.922	2.090	1.100	1.720	0.620	0.364
PC ae C44:3	11	0.074	0.073	0.042	0.111	0.048	0.096	0.048	0.024
PC ae C44:4	11	0.241	0.242	0.148	0.324	0.196	0.286	0.090	0.055
PC ae C44:5	11	1.247	1.220	0.526	1.980	0.919	1.570	0.651	0.434
PC ae C44:6	11	0.800	0.775	0.542	1.340	0.610	0.936	0.326	0.222
SM (OH) C14:1	11	4.487	4.140	2.970	6.940	3.710	5.590	1.880	1.236
SM (OH) C16:1	11	2.266	2.150	1.510	3.430	1.780	2.780	1.000	0.602
SM (OH) C22:1	11	7.995	7.380	5.200	15.000	6.270	8.840	2.570	2.681
SM (OH) C22:2	11	7.055	6.550	4.670	10.900	5.490	8.670	3.180	1.945
SM (OH) C24:1	11	0.711	0.665	0.419	1.320	0.559	0.824	0.265	0.242
SM C16:0	11	89.909	86.700	75.900	115.000	80.100	99.300	19.200	12.564
SM C16:1	11	11.305	10.600	9.020	15.100	9.920	12.600	2.680	1.853
SM C18:0	11	15.518	15.400	11.300	18.900	13.800	18.500	4.700	2.487
SM C18:1	11	6.884	6.800	4.470	8.980	5.990	7.770	1.780	1.382
SM C20:2	11	0.194	0.197	0.117	0.249	0.169	0.240	0.071	0.045
SM C22:3	11	101.000	101.000	101.000	101.000	101.000	101.000	0.000	0.000
SM C24:0	11	11.670	12.100	8.100	17.400	9.650	13.000	3.350	2.601
SM C24:1	11	36.964	36.100	28.700	45.100	32.100	43.000	10.900	5.440
SM C26:0	11	0.089	0.083	0.039	0.153	0.062	0.117	0.055	0.035
SM C26:1	11	0.229	0.212	0.132	0.421	0.171	0.262	0.091	0.081

The doses of levothyroxine were carefully selected (based on information such as patients’ weight, age, and other medical conditions) to maintain euthyreosis in hypothyroidic patients. There were no differences in the euthyroidic levels of TSH, fT4, and fT3 between both the hypothyroidic groups and the healthy control—see the results section.

The study participants were not receiving any other medical treatment. All members of the control and study groups were from the same geographic area (central and south-eastern Poland). Neither the control nor the two groups with hypothyroidism presented any pathologies (except the hypofunction of the thyroid gland), and they had taken no mineral or vitamin supplements for at least 3 months before the samples for analysis were collected.

They were not on any special diets during the tests. Other exclusion criteria included the presence of a chronic condition (particularly affecting the function of the thyroid gland or weight or limiting the patient’s ability to participate in the study), use of lipid-altering drugs, and refusal to give informed consent.

### Collection of the samples

Fasting blood specimens for lipidomic studies were collected from the patients and control groups into commercially available sterile anticoagulant-treated tubes, e.g., EDTA-treated (VACUETTE® K2E K2EDTA*)*. For Se measurements blood plasma was obtained by centrifuging heparinized whole blood (VACUETTE® TUBE NH Trace Elements Sodium Heparin (Greiner-Bio-One, Austria). All blood samples were separated for plasma immediately after collection and stored in − 80 °C freezers until laboratory analyses. Serum was used for routine diagnostic measurements (such as cholesterol, insulin, and thyroid hormones). Serum was collected in non-anticoagulant sterile tubes with a serum separator.

Serum levels of TC and TG were determined using enzymatic methods, and HDL-C was measured by immunoassay. The Friedewald formula using determined TC, HDL-C, and TG levels and the adopted TG-to-VLDL-C ratio was used to calculate the LDL-C level (LDL-C = TC – HDL-C – TG/5 (mg/dl))^22^.

### Targeted metabolomic analysis (TMA)

We applied TMA because such an analytical approach has better sensitivity and quantitative abilities than untargeted approaches where the metabolic, including lipid species of interest, are not predefined^23^. The quantifications of 145 lipids (such as 40 acylcarnitines, 90 glycerophospholipids, and 15 sphingomyelins) were undertaken. AbsoluteIDQ p180 kit (Biocrates Life Sciences AG, Innsbruck, Austria) analyzed metabolites. Sample preparation was performed following the manufacturer’s protocol. Briefly, 10 µl of plasma was pipetted onto the filter plate of the kit. Subsequently, 10 µl internal standards were added, and the plate was dried under a nitrogen stream. The samples were derivatized using phenylisothiocyanate and again dried under a nitrogen stream. Metabolites were extracted using 5 mm ammonium acetate in methanol and further diluted for LC–MS and FIA-MS experiments. Analyses were performed on an Agilent Infinity II 1290 HPLC coupled to an Agilent triple quadrupole mass spectrometer 6470 TQ LC/MS (Agilent Technologies, Santa Clara, CA, USA). Acquisition methods were set as provided by Absolute Biocrates for the p180 kit. Data acquisition was performed by Mass Hunter Acquisition B.10.0 (Agilent Technologies, Santa Clara, CA, USA). Data analysis was performed using MetIDQ (Biocrates, Innsbruck, Austria) and Mass Hunter Quantitative (Agilent Technologies, Santa Clara, CA, USA).

### Determination of se in plasma by inductively coupled plasma mass spectrometry (ICP-MS)

The plasma samples were dissolved with the acidic treatment in the microwave-assisted digestion system Ethos Up—Advanced Microwave Digestion Labstation (Milestone Srl, Italy) with user-selectable output power (0–1800W with 1W increment). Microwave-assisted acid digestion of samples was performed using a polypropylene rotor equipped with high-pressure Teflon vessels. The samples after the thawing were homogenized by sonification (15 min) and vortexing (30 s). Next, the plasma samples underwent microwave mineralization with 3.5 mL of 65% HNO_3_ Suprapur® grade and 1.5 mL of deionized water (DI water, conductivity < 0.08 µS/cm, HLP10 system, Hydrolab, Poland). The samples were dissolved with (DI) water up to a final volume of 7 mL. Solutions were stored at 4 °C prior to ICP-MS measurements.

The selenium standard solution was made from an individual standard (1.000 mg/L, TraceCERT®, Switzerland), and the calibration curves were prepared within range in the range of 0.2–50 µg/L. All solutions were prepared in freshly rinsed vials (with 1:1 nitric acid and deionized water (DI) at least three times). After microwave digestion, the plasma samples were diluted in the solution of 6% HNO_3_ in deionized water to reduce non-spectral interferences.

The total contents of Se in the plasma samples were measured by XSeries 2 ICP-MS (Thermo Fisher Scientific, Bremen, Germany) with PlasmaLab software, equipped with a collision/reaction cell operated using 7% H_2_ in He mixture gas (Linde Gaz Polska, Poland), used in conjunction with an ASX-510 autosampler (CETAC, Omaha, Nebraska, USA). The advantage of using ICP-MS is that it detects both organic and inorganic forms of Se in samples. Each sample was analyzed in triplicate, and the FullQuant analysis method was used to quantify the data. The linearity of the calibration curves was evaluated by the respective correlation coefficients (r2) = 0.9999; the LOD of the method was based on a 3 × standard deviation of 100 analytical blanks. To validate the method, the certified reference materials were used. We used both non-matrix matched CRMs – EP-H-2 (EnviroMAT Drinking Water), and EU-H-3 (EnviroMAT Waste Water) (SCP Science, Quebec, ON, Canada), as well as Seronorm™ Serum Seronorm Trace Elements serum L2 (Sero, Billingstad, Norway), where the concentration of Se was within the control range specified by the manufacturer (120–157 µg/L). Recoveries were in the range of 95.00–104.17% (Supplementary Table S1).

### Statistical analysis

Descriptive statistics were stratified by the presence of hypothyroidism. As the Kolmogorov–Smirnov and Lilliefors tests indicated that the variables were not normally distributed, the nonparametric Kruskal–Wallis ANOVA test was used for continuous variables. A multiple linear regression analysis was used to evaluate the association between the presence of hypothyroidism and plasma selenium levels as well as lipid species. All analyses were two-tailed, with a significance level of 0.05 and a power of 80%. Statistical analyses were performed using TIBCO Software Inc. (2017) Statistica, version 13.0.0.0 (TIBCO, Tulsa, OK, USA), licensed to the Medical University of Lublin.

## Results

A detailed characterization of the patients with Hashimoto’s disease or non-autoimmune hypothyroidism (Hypo-non-Hashimoto), as well as control, is presented in Tables 1, 2, and 3, respectively. As the variables were not normally distributed and nonparametric median tests were used to compare samples, the mean and standard deviation values are presented only to completely characterize the study groups. All the groups were age-, sex-, and BMI-matched. There were no significant differences in TSH or free thyroid hormone (fT4 and fT3) levels between the two hypothyroid groups (which included patients adequately treated with levothyroxine to maintain euthyreosis) and the healthy control. Furthermore, there were no significant differences in lipid profile, including total cholesterol (*p* = 0.13), LDL-cholesterol (*p* = 0.94), HDL-cholesterol (*p* = 0.09), and triglycerides (*p* = 0.16), as well as fasting glucose (*p* = 0.23), fasting insulin (*p* = 0.79), and HOMA-IR (*p* = 0.87) values between the Hashimoto’s disease group, non-autoimmune hypothyroidism (Hypo-non-Hashimoto) group and the healthy control (data not shown graphically).

**Table 3:** Clinical profile of the control group.

Variable	Control								
	Valid N	Mean	Median	Minimum	Maximum	Lower quartile	Upper quartile	Quartile range	Std. dev
Age (years)	6	27.167	23.000	23.000	45.000	23.000	26.000	3.000	8.818
BMI (kg/m^2^)	6	21.707	22.130	18.670	25.380	18.730	23.200	4.470	2.630
TSH (uIU/mL)	6	1.585	1.460	1.040	2.520	1.320	1.710	0.390	0.509
fT4 (ng/dL)	6	1.333	1.350	0.990	1.550	1.320	1.440	0.120	0.188
fT3 (pg/mL)	6	3.235	3.295	2.990	3.360	3.110	3.360	0.250	0.151
Total cholesterol (mg/dL)	6	153.333	148.000	134.000	191.000	137.000	162.000	25.000	21.869
HDL cholesterol (mg/dL)	6	51.833	53.000	41.000	62.000	45.000	57.000	12.000	7.705
LDL cholesterol (mg/dL)	6	90.500	87.000	69.000	123.000	83.000	94.000	11.000	17.952
Triglycerides (mg/dL)	6	62.667	65.000	32.000	83.000	57.000	74.000	17.000	17.750
AIP	6	0.068	0.087	-0.108	0.216	0.000	0.127	0.127	0.113
Fasting glucose (mg/dL)	6	93.000	93.500	86.000	99.000	89.000	97.000	8.000	5.099
Fasting insulin (mIU/L)	6	7.700	7.850	4.400	10.400	5.600	10.100	4.500	2.576
HOMA-IR	6	1.772	1.812	0.967	2.469	1.341	2.228	0.887	0.607
Se (µg/L)	6	130.529	131.388	111.057	152.500	112.690	144.153	31.463	16.899
Lipid species									
lysoPC a C14:0	6	1.832	1.645	1.380	2.730	1.500	2.090	0.590	0.503
lysoPC a C16:0	6	121.383	126.000	85.300	143.000	116.000	132.000	16.000	20.018
lysoPC a C16:1	6	3.065	2.890	2.150	4.810	2.310	3.340	1.030	0.974
lysoPC a C17:0	6	1.890	1.815	1.530	2.510	1.550	2.120	0.570	0.377
lysoPC a C18:0	6	35.117	32.400	26.300	49.400	27.900	42.300	14.400	9.120
lysoPC a C18:1	6	30.483	31.450	21.300	41.000	25.400	32.300	6.900	6.739
lysoPC a C18:2	6	44.517	41.300	36.400	62.500	39.100	46.500	7.400	9.423
lysoPC a C20:3	6	2.553	2.705	1.880	2.970	2.090	2.970	0.880	0.465
lysoPC a C20:4	6	7.463	7.970	3.940	10.100	5.890	8.910	3.020	2.262
lysoPC a C24:0	6	0.231	0.191	0.126	0.491	0.154	0.236	0.082	0.134
lysoPC a C26:0	6	0.444	0.281	0.117	1.380	0.176	0.429	0.253	0.474
lysoPC a C26:1	6	0.284	0.193	0.094	0.750	0.122	0.351	0.229	0.248
lysoPC a C28:0	6	0.339	0.255	0.143	0.898	0.144	0.337	0.193	0.285
lysoPC a C28:1	6	0.440	0.295	0.208	1.140	0.210	0.492	0.282	0.361
PC aa C24:0	6	0.140	0.096	0.048	0.346	0.065	0.187	0.122	0.112
PC aa C26:0	6	0.889	0.699	0.409	1.920	0.415	1.190	0.775	0.589
PC aa C28:1	6	2.160	2.130	1.630	2.810	1.640	2.620	0.980	0.540
PC aa C30:0	6	2.517	2.460	1.890	3.600	2.030	2.660	0.630	0.614
PC aa C30:2	6	101.000	101.000	101.000	101.000	101.000	101.000	0.000	0.000
PC aa C32:0	6	9.858	9.980	7.540	11.600	9.350	10.700	1.350	1.410
PC aa C32:1	6	11.228	8.870	6.300	24.300	6.930	12.100	5.170	6.825
PC aa C32:2	6	2.400	1.910	1.560	4.840	1.570	2.610	1.040	1.254
PC aa C32:3	6	0.315	0.289	0.256	0.442	0.274	0.338	0.064	0.068
PC aa C34:1	6	186.500	189.000	118.000	260.000	142.000	221.000	79.000	53.902
PC aa C34:2	6	373.500	349.000	287.000	495.000	306.000	455.000	149.000	85.971
PC aa C34:3	6	12.067	10.195	7.370	22.400	8.340	13.900	5.560	5.604
PC aa C34:4	6	1.054	0.982	0.645	1.880	0.694	1.140	0.446	0.451
PC aa C36:0	6	0.620	0.483	0.392	1.170	0.441	0.750	0.309	0.297
PC aa C36:1	6	32.033	30.000	22.400	41.300	29.200	39.300	10.100	7.049
PC aa C36:2	6	184.500	171.000	149.000	268.000	164.000	184.000	20.000	42.486
PC aa C36:3	6	92.250	87.400	68.700	120.000	73.000	117.000	44.000	22.552
PC aa C36:4	6	134.750	147.500	71.500	159.000	129.000	154.000	25.000	32.686
PC aa C36:5	6	16.890	18.200	5.640	24.900	10.800	23.600	12.800	8.102
PC aa C36:6	6	0.528	0.476	0.258	0.908	0.298	0.753	0.455	0.265
PC aa C38:0	6	1.822	1.645	1.350	2.520	1.580	2.190	0.610	0.439
PC aa C38:1	6	0.436	0.413	0.249	0.766	0.290	0.482	0.192	0.183
PC aa C38:3	6	25.600	26.700	18.800	28.700	25.000	27.700	2.700	3.578
PC aa C38:4	6	64.033	69.650	32.100	83.800	51.200	77.800	26.600	19.486
PC aa C38:5	6	32.467	33.150	16.300	42.200	31.900	38.100	6.200	8.856
PC aa C38:6	6	50.883	48.400	33.800	71.900	41.100	61.700	20.600	13.851
PC aa C40:1	6	0.287	0.275	0.265	0.326	0.265	0.315	0.050	0.026
PC aa C40:2	6	0.164	0.163	0.099	0.229	0.110	0.221	0.111	0.054
PC aa C40:3	6	0.300	0.312	0.179	0.373	0.266	0.356	0.090	0.071
PC aa C40:4	6	1.538	1.665	0.819	1.900	1.420	1.760	0.340	0.387
PC aa C40:5	6	4.623	5.205	2.200	5.550	4.060	5.520	1.460	1.308
PC aa C40:6	6	14.200	13.700	8.700	22.500	11.400	15.200	3.800	4.659
PC aa C42:0	6	0.371	0.385	0.214	0.498	0.277	0.464	0.187	0.108
PC aa C42:1	6	0.177	0.167	0.114	0.242	0.153	0.218	0.065	0.046
PC aa C42:2	6	0.137	0.127	0.110	0.190	0.110	0.159	0.049	0.032
PC aa C42:4	6	0.106	0.114	0.060	0.130	0.100	0.120	0.020	0.024
PC aa C42:5	6	0.203	0.190	0.128	0.293	0.174	0.243	0.069	0.057
PC aa C42:6	6	0.237	0.226	0.162	0.311	0.191	0.303	0.112	0.061
PC ae C30:0	6	0.275	0.287	0.216	0.297	0.270	0.296	0.026	0.030
PC ae C30:1	6	0.089	0.089	0.000	0.242	0.000	0.114	0.114	0.089
PC ae C30:2	6	0.081	0.072	0.052	0.131	0.057	0.100	0.043	0.030
PC ae C32:1	6	1.982	1.810	1.410	2.750	1.600	2.510	0.910	0.529
PC ae C32:2	6	0.493	0.472	0.372	0.668	0.410	0.561	0.151	0.107
PC ae C34:0	6	0.795	0.814	0.627	0.922	0.681	0.914	0.233	0.126
PC ae C34:1	6	7.875	7.385	6.550	10.000	6.880	9.050	2.170	1.358
PC ae C34:2	6	9.303	9.570	7.290	11.600	7.490	10.300	2.810	1.659
PC ae C34:3	6	5.805	5.580	4.750	7.570	4.850	6.500	1.650	1.071
PC ae C36:0	6	0.521	0.526	0.342	0.682	0.465	0.583	0.118	0.114
PC ae C36:1	6	5.003	5.260	3.740	5.920	4.040	5.800	1.760	0.916
PC ae C36:2	6	9.485	8.755	7.560	12.600	8.240	11.000	2.760	1.920
PC ae C36:3	6	5.528	5.365	4.390	7.080	4.450	6.520	2.070	1.086
PC ae C36:4	6	12.562	14.350	7.840	15.000	9.030	14.800	5.770	3.236
PC ae C36:5	6	7.058	7.650	4.200	9.050	4.810	8.990	4.180	2.090
PC ae C38:0	6	1.324	1.360	0.794	1.770	0.908	1.750	0.842	0.428
PC ae C38:1	6	0.171	0.088	0.000	0.604	0.039	0.208	0.169	0.223
PC ae C38:2	6	1.020	1.007	0.553	1.610	0.762	1.180	0.418	0.363
PC ae C38:3	6	1.980	1.940	1.640	2.340	1.720	2.300	0.580	0.299
PC ae C38:4	6	7.628	8.350	5.490	9.110	5.730	8.740	3.010	1.592
PC ae C38:5	6	12.338	13.200	7.420	16.300	8.810	15.100	6.290	3.506
PC ae C38:6	6	4.462	4.170	3.090	6.170	3.460	5.710	2.250	1.228
PC ae C40:1	6	0.768	0.735	0.498	1.020	0.650	0.968	0.318	0.196
PC ae C40:2	6	0.981	1.014	0.741	1.160	0.809	1.150	0.341	0.190
PC ae C40:3	6	0.617	0.582	0.510	0.753	0.542	0.735	0.193	0.102
PC ae C40:4	6	1.443	1.410	0.979	1.930	1.050	1.880	0.830	0.419
PC ae C40:5	6	2.050	2.010	1.530	2.780	1.570	2.400	0.830	0.538
PC ae C40:6	6	2.533	2.345	2.000	3.460	2.050	3.000	0.950	0.588
PC ae C42:0	6	0.363	0.361	0.276	0.452	0.288	0.442	0.154	0.074
PC ae C42:1	6	0.185	0.187	0.108	0.241	0.172	0.213	0.041	0.044
PC ae C42:2	6	0.320	0.310	0.253	0.467	0.254	0.327	0.073	0.079
PC ae C42:3	6	0.521	0.536	0.362	0.611	0.505	0.574	0.069	0.086
PC ae C42:4	6	0.633	0.576	0.456	0.891	0.467	0.834	0.367	0.190
PC ae C42:5	6	1.598	1.615	1.150	2.140	1.170	1.900	0.730	0.411
PC ae C44:3	6	0.072	0.071	0.040	0.101	0.057	0.092	0.035	0.022
PC ae C44:4	6	0.287	0.265	0.176	0.424	0.226	0.367	0.141	0.093
PC ae C44:5	6	1.523	1.550	0.887	2.060	1.160	1.930	0.770	0.445
PC ae C44:6	6	0.893	0.860	0.638	1.130	0.747	1.120	0.373	0.207
SM (OH) C14:1	6	3.578	3.365	2.590	4.690	3.140	4.320	1.180	0.780
SM (OH) C16:1	6	1.882	1.835	1.470	2.310	1.730	2.110	0.380	0.294
SM (OH) C22:1	6	6.777	6.885	5.030	8.140	5.650	8.070	2.420	1.291
SM (OH) C22:2	6	5.658	5.745	4.200	6.740	5.270	6.250	0.980	0.883
SM (OH) C24:1	6	0.677	0.680	0.488	0.915	0.572	0.728	0.156	0.146
SM C16:0	6	80.700	83.600	55.100	94.600	72.800	94.500	21.700	15.156
SM C16:1	6	9.902	10.335	5.980	12.500	8.060	12.200	4.140	2.603
SM C18:0	6	13.000	12.600	10.100	16.700	11.800	14.200	2.400	2.253
SM C18:1	6	5.770	5.870	4.390	7.180	4.610	6.700	2.090	1.152
SM C20:2	6	0.169	0.160	0.083	0.236	0.149	0.225	0.076	0.055
SM C22:3	6	101.000	101.000	101.000	101.000	101.000	101.000	0.000	0.000
SM C24:0	6	12.023	12.010	7.970	15.800	9.150	15.200	6.050	3.450
SM C24:1	6	33.683	33.600	22.900	41.500	32.000	38.500	6.500	6.416
SM C26:0	6	0.083	0.090	0.045	0.112	0.063	0.098	0.035	0.024
SM C26:1	6	0.214	0.190	0.174	0.321	0.176	0.232	0.056	0.057

**Fig. 1:**
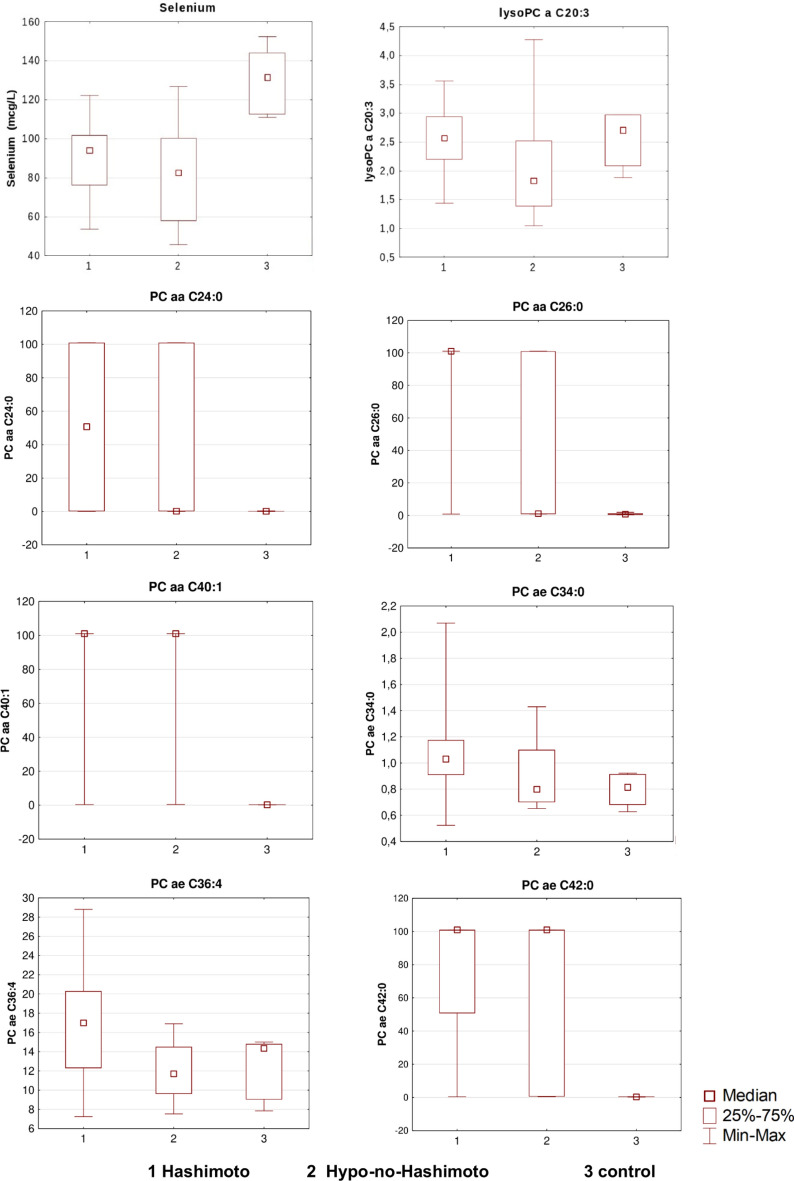
The significant Kruskal–Wallis ANOVA test results- plasma Se and lipids levels are presented as median values with interquartile ranges (IQR, 25–75%) and minimal/maximal values.

There were significant differences in selenium plasma levels between all hypothyroid patients (lower levels) and control (*p* = 0.005). However, there were no differences in selenium plasma levels between the two groups of patients with hypothyroidism (multiple comparisons of mean ranks are available in Fig. 1 and Table 4).

**Table 4:** Multiple comparisons of *p* values (2-tailed) of selenium plasma levels and selected lipid species in three groups: Hashimoto’s disease, non-autoimmune hypothyroidism (Hypo-non-Hashimoto), and healthy control.

Se (µg/L)	Kruskal–Wallis test: H ( 2. N = 41) = 11.82093 *p* = .0027		
	Hashimoto	Hypo-non- Hashimoto	Control
Hashimoto		1.000000	0.004944*
Hypo-non-Hashimoto	1.000000		0.003781*
Control	0.004944*	0.003781*	
LysoPC a C20:3	Kruskal–Wallis test: H ( 2. N = 41) = 6.675612 *p* = .0355		
Hashimoto		0.044077*	1.000000
Hypo-non-Hashimoto	0.044077*		0.157538
Control	1.000000	0.157538	
PC aa C24:0	Kruskal–Wallis test: H ( 2. N = 41) = 6.544281 *p* = .0379		
Hashimoto		1.000000	0.039785*
Hypo-non-Hashimoto	1.000000		0.279034
Control	0.039785*	0.279034	
PC aa C26:0	Kruskal–Wallis test: H ( 2. N = 41) = 15.82482 *p* = .0004		
Hashimoto		0.210365	0.001894*
Hypo-non-Hashimoto	0.210365		0.227979
Control	0.001894*	0.227979	
PC aa C40:1	Kruskal–Wallis test: H ( 2. N = 41) = 31.40706 *p* = .0000		
Hashimoto		1.000000	0.000443*
Hypo-non-Hashimoto	1.000000		0.003090*
Control	0.000443*	0.003090*	
PC ae C30:1	Kruskal–Wallis test: H ( 2. N = 41) = 8.596205 *p* = .0136		
Hashimoto		0.908467	0.025399*
Hypo-non-Hashimoto	0.908467		0.310172
Control	0.025399*	0.310172	
PC ae C34:0	Kruskal–Wallis test: H ( 2. N = 41) = 7.436659 *p* = .0243		
Hashimoto		0.370555	0.031015*
Hypo-non-Hashimoto	0.370555		0.688991
Control	0.031015*	0.688991	
PC ae C36:4	Kruskal–Wallis test: H ( 2. N = 41) = 8.468897 *p* = .0145		
Hashimoto		0.020913*	0.250969
Hypo-non-Hashimoto	0.020913*		1.000000
Control	0.250969	1.000000	
PC ae C42:0	Kruskal–Wallis test: H ( 2. N = 41) = 17.34746 *p* = .0002		
Hashimoto		1.000000	0.000601*
Hypo-non-Hashimoto	1.000000		0.014468*
Control	0.000601*	0.014468*	

Only significant values (marked with *) are presented.

Interestingly, lysoPC a C20:3 was lower (*p* = 0.04) in the Hypo-non-Hashimoto group than in the Hashimoto’s disease group and the control group (CG). PC aa C26:0 (*p* = 0.002), PC ae C30:1 (*p* = 0.025), PC ae C34:0 (*p* = 0.03), as well as PC aa C24:0 (*p* = 0.04), were higher in the group with Hashimoto’s disease than in the CG. There were no significant differences in the two hypothyroid groups for these lipids. PC aa C40:1 (*p* = 0.0004), as well as PC ae C42:0 (*p* = 0.0006), were higher in all hypothyroid patients in comparison to CG, and PC ae C36:4 was higher (*p* = 0.02) **i**n the group with the Hashimoto’s disease than in the group Hypo-non-Hashimoto, but not in CG, as it is seen in Fig. 1 and Table 4.

All values, both significant and not significant regarding the multiple comparison *p* values (two-sided) of plasma selenium levels and selected lipid species in the three groups are presented in supplementary Table S2.

As it is seen in Table 5, PC aa C26:0 and PC aa C40:1 were negatively correlated with plasma selenium concentrations. These lipid species also presented significant differences in the Kruskal–Wallis ANOVA test (see above). Other lipids that were negatively correlated with plasma selenium concentrations but did not present any significant differences between the three groups in the Kruskal–Wallis ANOVA test were PC aa C32:0, PC ae C30:0, PC ae C36:5, SM C18:0, and SM C18:1.

**Table 5:** The Spearman rank order – correlations of plasma selenium levels with lipid species. Only significant values are presented.

Variable	Se (µg/L)
HDL cholesterol (mg/dL)	− 0.323333
Triglycerides (mg/dL)	− 0.386700
PC aa C26:0	− 0.421332
PC aa C32:0	− 0.311255
PC aa C40:1	− 0.496637
PC ae C30:0	− 0.355506
PC ae C36:5	− 0.319829
SM C18:0	− 0.354579
SM C18:1	− 0.312337

In the multiple linear regression analyses (Table 6), plasma selenium levels showed a negative relationship with the presence of hypothyroidism. PC aa C24:0, PC aa C26:0, PC ae C30:1, PC ae C34:0, PC ae C36:4, PC ae C42:0 were positively associated with the presence of hypothyroidism.

**Table 6:** Multiple linear regression analyses of the association between the presence of hypothyroidism and plasma selenium levels as well as selected lipid species.

Regression summary N = 41	Se (µg/L)					
	b*	Std.Err. of b*	b	Std.Err. of b	t(39)	*p*-value
Hashimoto 1 Hypo-non-Hashimoto 2 control 3	0.413249	0.145816	14.65842	5.172253	2.834049	0.007243
PC aa C24:0						
Hashimoto 1 Hypo-non-Hashimoto 2 control 3	− 0.338973	0.150648	− 22.7159	10.09553	− 2.25010	0.030159
PC aa C26:0						
Hashimoto 1 Hypo-non-Hashimoto 2 control 3	− 0.572336	0.131308	− 38.3971	8.80924	− 4.35873	0.000092
PC ae C30:1						
Hashimoto 1 Hypo-non-Hashimoto 2 control 3	− 0.462195	0.141998	− 31.5287	9.68642	− 3.25494	0.002348
PC ae C34:0						
Hashimoto 1 Hypo-non-Hashimoto 2 control 3	− 0.370008	0.148764	− 0.149118	0.059954	− 2.48722	0.017259
PC ae C36:4						
Hashimoto 1 Hypo-non-Hashimoto 2 control 3	− 0.408951	0.146126	− 2.64950	0.946718	− 2.79862	0.007936
PC ae C42:0						
Hashimoto 1 Hypo-non-Hashimoto 2 control 3	− 0.503739	0.138328	− 33.9922	9.33434	− 3.64163	0.000786

## Discussion

It is known that a wide range of hormones regulate lipid metabolism simultaneously in a time-specific manner. Thyroid hormones regulate and affect both cholesterol and fatty acid metabolism. They stimulate fatty acid synthesis (lipogenesis), triglyceride breakdown (lipolysis), fatty acid oxidation, cholesterol synthesis, and low-density lipoprotein (LDL) receptors. Genes involved in lipogenesis in the liver and positively regulated by HT include fatty acid synthase (FAS), acetyl-CoA carboxylase (ACC), spot14 protein, and malate enzyme (ME) genes. Fatty acid oxidation-related genes positively regulated by HT include carnitine acyltransferase (CPT), acyl-CoA translocase (TAC), and long-chain fatty acid oxidase (AOX) genes. Cholesterol synthesis is influenced by T3 by stimulating the expression of the hydroxymethylglutaryl-CoA reductase (RHMG) gene and the activity of this enzyme. Inhibition by T3 of the expression of cholesterol 7α-hydroxylase (CYP7A1) contributes to the reduction of bile acid synthesis^24^. Very recently, Zhang et al.^25^ provided new insight into hormones regulating lipid metabolism and highlighted the role of thyroid hormone receptors (THR) in lipid metabolism. Although the effect of THRs and subsequent pathways in lipid metabolism is still under investigation, this research, citing animal studies, emphasizes that THs not only directly regulate lipogenic gene expression but also affect the activity of other transcription factors, such as sterol regulatory element binding protein-1c (SREBP1c) and carbohydrate-responsive element-binding protein (ChREBP), indirectly influencing hepatic lipogenesis^26^. THs promote the lipolysis of white adipose tissue, which is a source of circulating free fatty acids (FFAs), induces the protein transporter expression such as fatty acid transporter proteins (FATPs), liver fatty acid-binding proteins (L-FABPs) and fatty acid translocase^27^.

Thyroid hormones, such as thyroxine (T4) and triiodothyronine (T3), exert their effects by binding to specific nuclear receptors, which then modulate gene expression^24^. Proper membrane structure is essential for the function and localization of these receptors. Its integrity and fluidity are precisely ensured by, for example, glycerophospholipids^28^. The membrane of thyrocytes contains thyroid peroxidase (TPO), an enzyme involved in the synthesis of thyroid hormones, and proteins involved in the processes of iodine uptake, incorporation of this element into thyroglobulin, as well as the release of thyroid hormones into the bloodstream and their transport^29^. In addition, glycerophospholipids may play a role in immune system recognition and response, so changes in membrane composition could potentially affect antigen presentation and immune responses, contributing to the development of autoimmune diseases such as Hashimoto’s^30^.

The imbalance between free radical production and antioxidant defense, is associated with damage to a wide range of molecular species, including lipids, proteins and nucleic acids^31^. Oxidative damage affecting glycerophospholipids, among others, leads to lipid peroxidation and the release of inflammatory molecules, exacerbating thyroid dysfunction^30^. Lipid peroxidation occurs when a hydroxyl radical strips an electron from an unsaturated fatty acid. The unstable lipid radical that forms can then react with oxygen to form a fatty acid peroxyl radical can react with another unsaturated fatty acid. As a result, the generated fatty acid hydroperoxide and new lipid radical promote damage to cell membranes^32^. Free radicals play a very important role in the functioning and regulation of the immune system^33^. They intensify the activation of T lymphocytes and cause leukocyte cells to fuse with the endothelium, allowing them to move from the circulatory system to the site of the inflammatory response. Reactive oxygen species are signaling molecules, in their action very similar to hormone messengers and hormones themselves^34^. Oxidative stress is implicated in multiple diseases, including diabetes, obesity, neurological diseases, cardiovascular disease and cancer^35^.

Research on oxidative stress indicates associations of thyroid diseases, including thyroid gland tumors, as well as autoimmune thyroid disease^36,37^. Hashimoto’s thyroiditis is likely related to iodide-mediated oxidative stress and inflammation^38^. Thyroid disorders may initiate or increase ROS release and oxidative stress, enhancing oxidative damage, which appears to be involved in both the initiation and progression of carcinogenesis^37^.

With advances in analytical methodologies and techniques for determining compounds of indisputable relevance in health and disease states, such as lipids, there has been an increased interest in individual metabolites. Lipidomics, belonging to the field of metabolomics, may be used to investigate metabolic changes in various diseases^39^. Recent studies have shown an association between the results of lipidomic profiling and selected disease onset and progression^40,41^. Lipidomics involves complex lipidome analysis and is quite challenging due to the vast number of lipid species. Owing to their considerable structural diversity, lipids are important players in various complex physiological processes where they execute important functions, acting as cellular membrane components, signaling mediators, energy reserve molecules, and endocrine regulators^42^. It has been revealed that not only the main “clinical” lipids (TG, CHOLs) are useful during routine diagnostics and treatment, but there is also a great hope among scientists that analysis of the human lipidome will help in detecting and monitoring an increasing number of human diseases.

An essential advantage of lipidomics is the ability to identify specific plasma lipid abnormalities, e.g., changes in molecular species containing particular-chain-length fatty acids. The specific molecular changes would remain undetected if one were using the enzymatic lipid assays that are routinely applied in clinical laboratories to determine total lipid (TL) content in serum. In our study, the following lipid classes: acylcarnitines (more specifically, a plasma acylcarnitine profile can aid in the diagnosis of organic acidemias in addition to fatty acid oxidation disorders), glycerophospholipids (structural components of biological membranes, important constituents of lipoproteins, performing functions in other cellular processes such as signal induction and transport), and sphingomyelins (having significant structural and functional roles in the cell, as plasma membrane components and as participants in many signaling pathways) were identified and quantified. Our results suggest that among almost two hundred metabolites, which we could quantify using targeted metabolomics analysis, only a few correlated with hypothyroidism and Hashimoto disease (see Tables 4, 5, and 6).

Although glycerophospholipids are known to dominate cell membranes, providing stability, fluidity and permeability, are required for the proper function of membrane proteins, receptors and ion channels, and act as reservoirs of second messengers and their precursors, not all species of this broad class of lipids are well understood in the context of interactions with thyroid hormones. The different combinations of glycerophospholipid head groups and fatty acyl chains give rise to thousands of molecular species^43^. Among them are glycerophospholipids such as PC ae C30:1, PC ae C36:5, and PC ae C42:0. According to our best knowledge, no studies describe a significant association of these molecules in any disease. Due to the complexity of metabolism and insufficient research, there is a lack of thorough information on the function of these specific lipids in the human body. There is no conclusive data that glycerophospholipids have a direct effect on the onset and development of thyroid diseases, such as hypothyroidism or Hashimoto’s, which are complex and often multifactorial, involving factors such as genetics, immune responses, hormonal imbalances, and also environmental factors^44,45^.

Although PC ae C34:0 has not been previously linked to hypothyroidism or Hashimoto’s, this lipid has been linked to other diseases. PC ae C34:0 levels have been characterized in three papers on Alzheimer’s disease (AD), colorectal cancer (CRC), and VZV (chickenpox and hemiplegia virus) meningitis. In a study performed by Huo et al.^46^ concerning Alzheimer’s disease, elevated serum levels of this lipid measured prior to diagnosis (AD) predicted a faster decline in global cognition and three cognitive domains (episodic memory, perceptual speed, semantic memory). At the same time, it showed protective effects on neuropathology in brain tissue samples. It is also suggested that this lipid may be a predictor of high risk of colorectal cancer recurrence in the next 6 months after hepatectomy^47^.

Furthermore, PC ae C34:0 can be considered as a biomarker in the diagnosis of meningitis resulting from VZV since, in addition to the other lipids mentioned, its concentration characterized inflammation and pathological processes in parenchymal cells of the central nervous system, without a clear effect on the number of leukocytes in the cerebrospinal fluid^48^. PC ae C36: 4 was characterized in the study on determining metabolic changes after metformin use to treat type 2 diabetes mellitus (T2DM)^49^. A significant decrease in phosphatidylcholine PC ae C36:4 was demonstrated not with the first dose of this drug but with its prolonged use in the 4–6 weeks range. PC aa C36:4 was also demonstrated to be positively associated with BMI^50^.

Interestingly, in our study the analyses of the association between the presence of hypothyroidism and plasma selenium levels as well as selected lipid species revealed significant values for phosphatidylcholines (PCs) which are among the major constituents of cell membranes and represent important components of lipoproteins^51^. Out of 145 different lipid molecules analyzed qualitatively by TLA in the present study for only six PCs, i.e. PC aa C24:0, PC aa C26:0, PC ae C30:1, PC ae C34:0, PC ae C36:4, PC ae C42:0 (aa indicating two acyl-bound and ae indicating one acyl- and one alkyl-bound fatty acids) the mentioned significant associations were found.

Most research on sphingomyelin (SM) has focused on its role in cell membrane structure, myelin sheath formation, neurological functions, and various cell signaling pathways^52–55^. Although sphingolipids, including sphingomyelins, are known to be involved in cell signaling and membrane functions, their specific involvement in thyroid diseases, such as hypothyroidism or Hashimoto’s thyroiditis, has not been thoroughly studied or established. Sphingolipids, like glycerophospholipids, are major components of cell membranes, contributing to their structural integrity and fluidity. They are particularly abundant in the outer layer of the plasma membrane, where they help form lipid rafts—microdomains that play a role in signaling, protein transport, and membrane organization. They participate in intracellular membrane transport and vesicle formation during endocytosis, exocytosis, and vesicular transport. Sphingolipids are involved in various signaling pathways that regulate cell growth, differentiation, survival, and apoptosis (programmed cell death). They can modulate immune responses by affecting immune cell activity and cytokine secretion^56^. Both the de novo synthesis of sphingolipids and the sphingomyelinase pathway are important in the pathogenesis of some auto-immune disorders, e.g., autoimmune encephalomyelitis^57^ or arthritis^58^.

SMs are major components of cell membranes, particularly abundant in the outer layer of the plasma membrane. Along with cholesterol, SMs contribute to membrane stability, fluidity, and lipid raft formation. This interaction is vital for maintaining the proper balance between rigidity and elasticity of cell membranes. It is also a key component of myelin sheaths in neurons. Contributing to the insulation and integrity of nerve fibers, it enables efficient transmission of nerve signals. Sphingomyelin can act as a reservoir of enzymes involved in sphingolipid metabolism, such as sphingomyelinases. As a result of their action, bioactive lipid molecules can be formed, including ceramides, which are involved in cell signaling^59^. Disruption of sphingomyelin metabolism is associated with various diseases, including Niemann-Pick disease, lysosomal storage disorder, and arteriosclerosis, in which the accumulation of sphingomyelin in blood vessels can contribute to the formation of atherosclerotic plaques^60,61^.

Research revealed a significant association of C18:1 sphingomyelin (SM) with multisite musculoskeletal pain (MSMP)^62^ with alcohol use and smoking^63^, liver cirrhosis^64^, and also in association with other sphingomyelins, contribute to T2D through effects on BMI^50^. Elevated levels of SM C18 have been reported as a probable predictor of Alzheimer’s disease^65^, diagnosis of insulin-related disorders, and especially type 1 diabetes mellitus (T1DM) and latent autoimmune diabetes in adults (LADA)^66^. In addition, increased levels of this sphingomyelin were detected in a study group with endocrine hypertension (EHT)^67^, hyperglycemic patients were more likely to develop chronic kidney disease (CKD)^68^, and in patients with polycystic ovary syndrome (PCOS)^69^. Significantly reduced levels of SM C18:1 were reported in studies^70^ concerning only peritoneal fluid samples from patients diagnosed with endometriosis.

To the best of our knowledge, the present study is the first to demonstrate the possible association of several components of the human lipidome with hypothyroidism. Our finding is significant as it may provide a basis for further investigation to clarify the role of selected lipid molecules in hypothyroidism.

In our current study, we were interested in whether Se, a micronutrient whose excess is just as problematic as deficiency, may be associated with particular lipid species linked to hypothyroidism. The relationship between hypothyroidism and selenium levels is complex and bidirectional—hypothyroidism is associated with an increased risk of selenium deficiency. In contrast, selenium deficiency can exacerbate thyroid dysfunction because selenium is required to convert the inactive thyroid hormone T4 (thyroxine) into the active form T3 (triiodothyronine). Without sufficient selenium, this conversion process may be impaired, contributing to the symptoms of hypothyroidism. In individuals with hypothyroidism, the body’s metabolic rate is decreased, leading to reduced absorption of nutrients, including selenium, from the gastrointestinal tract—this can result in a feedback loop, further lowering selenium levels in the bloodstream. Selenium is thus regarded as one of the most potent antioxidants. The role of Se-containing selenoenzymes and selenoproteins is to speed up the oxidation reaction of proteins scavenging reactive oxygen species. Se protects the body from oxidative stress, which can cause immune disorders, cardiovascular disease, and cancer. Selenium deficiencies can lead to a significant weakening of the immune response. This is due to, among other things, a decrease in the activity of T lymphocytes, macrophages, and NK cells. Studies indicate that selenium supplementation causes a decrease in anti-thyroid peroxidase antibodies (anti-TPO) and also an increase in the ratio between FT3 and FT4 (selenium is an important component of the enzyme that converts thyroxine T4 into triiodothyronine T3)^71^. Khorasani et al.^71^ investigated Se status in patients categorized into two groups depending on the type of diagnosis, i.e., hypothyroidism and autoimmune thyroiditis, and found significant differences in serum concentrations of Se between hypothyroid patients (with a lower concentration of Se) and controls. However, no relationship was found between the type of thyroid disease and a pronounced selenium deficiency. The findings of Stojsavljević et al.^72^ indicated apparent differences in Se profiles between hypothyroidism and healthy subjects. However, in contrast to studies of Khorasani et al.^71^ significantly higher concentrations of selenium in the group of respondents suffering from hypothyroidism compared with healthy subjects were found.

Studies on the relationship between body selenium levels and hyperlidpidemia emphasize the role of oxidative stress and the inflammatory response^73^. This is not the only possible pathway, as selenium may interact with other elements and this may cause dyslipidaemia by encouraging the release of lipids from the liver and adipose tissues, resulting in decreased activity of antioxidant enzymes such as superoxide dismutase and catalase, increased lipid peroxide levels in the liver and kidneys and markers of abnormal liver function. Oxidation of cell membranes can potentially cause hepatotoxic effects that affect liver function and reduce lipid production in hepatocytes^74^. Studies showed that selenium deficiency increases lipid peroxidation in the membranes, and clearly leads to the cell death. Research confirmed that the lipid hydroperoxides, play a causative role in the oxidative damage to cells induced by selenium deficiency^75^.

Recent animal studies revealed that selenium deficiency in diet may adversely influence the fatty acid profile (e.g., conversion of linolenic acid (ALA) to eicosapentaenoic acid (EPA) and docosahexaenoic acid (DHA), resulting in the unbeneficial n-6/n-3 ratio in tissue lipids)^76^. A meta-analysis based on human studies that explored Se levels and the effect of Se supplementation on lipid profile (including total cholesterol (TC), triglyceride (TG), low-density lipoprotein (LDL), high-density lipoprotein (HDL), very low-density lipoprotein (VLDL)) measurements) suggested that the effect of selenium supplementation on the serum levels of TG and VLDL was marginally significant and that the effect of selenium supplementation on lipid profile was negative^77^. While it has been reported that Se may be associated with lipid levels, the available data seem to be conflicting^78^. Examining the relation of serum selenium concentrations with serum lipids in large, Se-replete men and women from the US revealed the association of Se and TC, LDL-C, HDL-C, TGs, apo B, and apo A-I. However, the cause-and-effect relations and the potential mechanisms underlying these associations remain unknown^79^. About two decades ago, a considerable number of Polish inhabitants had a relatively low concentration of Se in blood plasma—about 50–55 μg/l, and the calculated daily dietary intake was about 30–40 μg/day^80^. Selenium deficiency was diagnosed by measuring the serum or plasma selenium level, which should be at least 85 μg/L^81^. Recent studies on the Polish population report Se levels of approximately 79 μg/L in healthy adults^82^. Although various national and international guidelines often provide reference ranges, there is no specific data on Se levels in compensated (well-controlled) hypothyroidism in the Polish population of adults.

## Strong and weak points of the study

The great advantage of our work is that we follow very strict criteria for qualifying patients for the research. It is rare in patients suffering from hypothyroidism for the lipid profile—usually routinely prescribed for patients (total cholesterol, LDL, HDL, TG)- for glucose and insulin levels to be within the accepted norms. The exclusion of the most common fluctuations in biochemical parameters and the well-controlled levels of thyroid hormones in the study group is the basis for suspecting that individual species of the lipidome are typical of hypothyroidism itself, perfectly compensated without comorbidities. Both studied, and the control groups were age- and BMI-matched. Moreover, there were no statistically significant differences in TC, LDL-C, HDL-C, TGs, glucose/insulin, and degree of insulin resistance. We ensured that all participants in the study were not taking any medication, and dietary supplements that could affect the lipid profile. The project participants came from the same geographical region and were on a similar diet.

We indicated that the hypothyroid group had lower levels of Se than the controls (the normal concentration in adult human blood serum is between 110 and 165 mcg/L). There is a lack of such current data on this specific human population. In the state of selenium deficiency associated with loss of glutathione peroxidase activity, the serum concentration is usually below 40 mcg/L^83^. We assessed selenium status by measuring plasma selenium, which responds to changes in intake. It is essential to note that while selenium plays a role in thyroid function, not everyone with hypothyroidism will have its deficiency, and the relationship between the two can vary from person to person. In our study, we eliminated the results of patients whose selenium levels would have indicated a severe deficiency.

The study’s weakness is the small sample size, especially the control group. During recruitment for the study, many volunteers had to be excluded due to the diagnosis of other diseases. It also turned out that it was challenging to select a group of people who would meet the rigorous qualification criteria for our project. The power of our study is relatively low. However, the data acquired here may be considered preliminary for broader research. Interactions with other nutrients, such as vitamins and some minerals, may also influence the effects of selenium on plasma lipids. We will examine this in the next stage of our project.

Completing our future studies with the identified limitations may contribute to a deeper understanding of the complex relationship between Se and lipids’ metabolism and its modification by various thyroid pathologies. Moreover, considering that the biological effects and bioavailability of Se may strongly depend on its chemical forms and their amounts in diet^84,85^, our research could be extended to investigate speciation forms of that element and their distribution in different tissues, as well as their response to varying dietary selenium content.

## Conclusions

The present preliminary study is the first to demonstrate the association of several components of the human lipidome with hypothyroidism in relation to the total plasma selenium content. Different lipidome profiles were identified in healthy and hypothyroid patients regardless of the cause of that condition. Our studies emphasize the contributing role of Se in altered lipid metabolism in patients with hypothyroidism. The association between Se and particular lipid molecules was modified by thyroid pathology. Out of 145 different lipid molecules analyzed qualitatively by TLA in the present study only six PCs, i.e. PC aa C24:0, PC aa C26:0, PC ae C30:1, PC ae C34:0, PC ae C36:4, PC ae C42:0 were positively associated with the presence of hypothyroidism. Plasma selenium levels showed a negative relationship with the presence of hypothyroidism. Subsequent studies are required to elucidate the mechanisms of such association and clarify the role of particular lipid species in hypothyroidism.

## Supplementary Information

Supplementary Information 1.

Supplementary Information 2.

## Data Availability

The data that support the findings of this study are not openly available due to reasons of sensitivity and are available from the corresponding author upon reasonable request. Data are located in controlled access data storage at Medical University of Lublin.

## Abbreviations

a: Acyl residue
aa: Diacyl resid
ae: Acyl-alkyl residue
BMI: Body mass index
FG: Fasting plasma glucose
FINS: Fasting plasma insulin
FT3: Free triiodothyronine
FT4: Free thyroxine
HDL-C: High-density lipoprotein cholesterol
HOMA-IR: Homeostasis model assessment of insulin resistance
HT: Hashimoto thyroiditis
ICP-MS: Inductively coupled plasma mass spectrometry
LDL-C: Low-density lipoprotein cholesterol
LT4: Levothyroxine
lysoPC: Lysophosphatidylcholine
PC: Phosphatidylcholine
Se: Selenium
SM: Sphingomyelin
SM (OH): Hydroxysphingomyelin
TC: Total cholesterol
TG: Triglyceride
TgAb: Antithyroglobulin antibody
TLA: Targeted lipidomic analysis
TQ-LC/MS: Triple quadrupole liquid chromatography/mass spectrometry
TPOAb: Thyroid peroxidase antibody
TSH: Thyrotropin

## References

[1] Wang, J., Wang, C. & Han, X. Tutorial on lipidomics. *Anal. Chim. Acta.* **1061**, 28–41. 10.1016/j.aca.2019.01.043 (2019). 30926037 10.1016/j.aca.2019.01.043 PMC7375172

[2] Płaczkiewicz-Jankowska, E. & Jankowski, P. Diagnostyka i leczenie zaburzeń lipidowych u chorych z zaburzeniami endokrynnymi. Podsumowanie wytycznych the Endocrine Society 2020 z odniesieniem do wytycznych European Society of Cardiology (2019) i zaleceń Polskiego Towarzystwa Kardiologicznego, Polskiego Towarzystwa Lipidologicznego i Kolegium Lekarzy Rodzinnych w Polsce. *Med. Prakt.* **5**, 12–36 (2021).

[3] Newman, C. B. et al. Lipid management in patients with endocrine disorders: An Endocrine Society clinical practice guideline. *J. Clin. Endocrinol. Metab.* **105**, 1–70. 10.1210/clinem/dgaa674 (2020). 32951056 10.1210/clinem/dgaa674

[4] Płaczkiewicz-Jankowska, E. Leczenie niedoczynności tarczycy: podsumowanie wytycznych American thyroid association task force on thyroid hormone replacement. *Med. Prakt.* **5**, 12–26 (2014).

[5] Jonklaas, J. et al. American thyroid association task force on thyroid hormone replacement. Guidelines for the treatment of hypothyroidism: prepared by the american thyroid association task force on thyroid hormone replacement. *Thyroid.* **12**, 1670–1751. 10.1089/thy.2014.0028 (2014). 10.1089/thy.2014.0028 PMC4267409 25266247

[6] Duntas, L. H. Thyroid disease and lipids. *Thyroid* **12**, 287–293. 10.1089/10507250252949405 (2002). 12034052 10.1089/10507250252949405

[7] Luo, Y. et al. Assessment of the relationship between subclinical hypothyroidism and blood lipid profile: reliable or not?. *Lipids Health Disease* **21**, 137. 10.1186/s12944-022-01749-0 (2022). 10.1186/s12944-022-01749-0 PMC9746155 36514152

[8] Monzani, F. et al. Effect of levothyroxine replacement on lipid profile and intima-media thickness in subclinical hypothyroidism: A double-blind, placebo-controlled study. *J. Clin. Endocrinol. Metab.* **89**(5), 2099–2106. 10.1210/jc.2003-031669 (2004). 15126526 10.1210/jc.2003-031669

[9] Gu, Y. et al. Thyroid function and lipid profile in euthyroid adults: the TCLSIH cohort study. *Endocrine* **70**(1), 107–114. 10.1007/s12020-020-02312-6 (2020). 32328967 10.1007/s12020-020-02312-6

[10] Chin, K. Y. et al. The relationships between thyroid hormones and thyroid-stimulating hormone with lipid profile in euthyroid men. *Int J Med Sci.* **11**(4), 349–355. 10.7150/ijms.7104 (2014). 24578612 10.7150/ijms.7104 PMC3936029

[11] Li, X., Wang, Y., Guan, Q., Zhao, J. & Gao, L. The lipid-lowering effect of levothyroxine in patients with subclinical hypothyroidism: A systematic review and meta-analysis of randomized controlled trials. *Clin Endocrinol.* **87**, 1–9. 10.1111/cen.13338 (2017). 10.1111/cen.13338 28342184

[12] Newman, C. B. et al. Lipid management in patients with endocrine disorders: An endocrine society clinical practice guideline. *J. Clin. Endocrinol. Metabolism*10.1210/clinem/dgaa674 (2020). 10.1210/clinem/dgaa674 32951056

[13] Toh, P., Nicholson, J. L., Vetter, A. M., Berry, M. J. & Torres, D. J. Selenium in bodily homeostasis: Hypothalamus, hormones, and highways of communication. *Int. J. Mol. Sci.* **23**, 15445. 10.3390/ijms232315445 (2022). 36499772 10.3390/ijms232315445 PMC9739294

[14] Błażewicz, A., Wiśniewska, P. & Skórzyńska-Dziduszko, K. Selected essential and toxic chemical elements in hypothyroidism—A literature review (2001–2021). *Int. J. Mol. Sci.* **22**, 10147. 10.3390/ijms221810147 (2021). 34576309 10.3390/ijms221810147 PMC8472829

[15] Ventura, M., Melo, M. & Carrilho, F. Selenium and thyroid disease: from pathophysiology to treatment. *Int. J. Endocrinol.*10.1155/2017/1297658 (2017). 28255299 10.1155/2017/1297658 PMC5307254

[16] Duntas, L. H., Mantzou, E. & Koutras, D. A. Effects of a 6 month treatment with selenomethionine in patients with autoimmune thyroiditis. *Eur. J. Endocrinol.* **148**, 389–393 (2003). 12656658 10.1530/eje.0.1480389

[17] Larsen, C. B. et al. Selenium supplementation and placebo are equally effective in improving quality of life in patients with hypothyroidism. *Eur. Thyroid J.*10.1530/ETJ-23-0175 (2024). 38215286 10.1530/ETJ-23-0175 PMC10895332

[18] Gorini, F., Sabatino, L., Pingitore, A. & Vassalle, C. Selenium: An element of life essential for thyroid function. *Molecules* **23**, 7084. 10.3390/molecules26237084 (2021). 10.3390/molecules26237084 PMC8658851 34885664

[19] Leonidas, H. D. Selenium and the thyroid: A close-knit connection. *J. Clin. Endocrinol. Metabol.* **95**(12), 5180–5188. 10.1210/jc.2010-0191 (2010). 10.1210/jc.2010-0191 20810577

[20] Zhang, L. et al. Integrated microRNA and proteome analysis reveal a regulatory module in hepatic lipid metabolism disorders in mice with subclinical hypothyroidism. *Exper. Therapeutic Med.* **19**, 897–906 (2020). 10.3892/etm.2019.8281 PMC6966133 32010250

[21] Mazaheri-Tehrani, S. et al. Serum selenium levels and lipid profile: A systematic review and meta-analysis of observational studies. *Biol. Trace Elem. Res.*10.1007/s12011-024-04365-4 (2024). 39256333 10.1007/s12011-024-04365-4 PMC12125032

[22] Solnica, B. et al. Guidelines of the polish society of laboratory diagnostics (PSLD) and the polish lipid association (PoLA) on laboratory diagnostics of lipid metabolism disorders. *Arch. Med. Sci.* **16**(2), 237–252. 10.5114/aoms.2020.93253 (2020). 32190133 10.5114/aoms.2020.93253 PMC7069434

[23] Swinnen, J. V. & Rueda, N. A beginner’s guide to lipidomics. *The Biochemist* **44**, 1. 10.1042/bio_2021_181 (2022).

[24] Liu, Y. Y. & Brent, G. A. Thyroid hormone crosstalk with nuclear receptor signaling in metabolic regulation. *Trends Endocrinol. Metab.* **21**, 166–173 (2010). 20015660 10.1016/j.tem.2009.11.004 PMC2831161

[25] Zhang, D. et al. Important hormones regulating lipid metabolism. *Molecules* **27**(20), 7052. 10.3390/molecules27207052 (2022). 36296646 10.3390/molecules27207052 PMC9607181

[26] Wang, Y., Viscarra, J., Kim, S. J. & Sul, H. S. Transcriptional regulation of hepatic lipogenesis. *Nat. Rev. Mol. Cell Biol.* **16**, 678–689. 10.1038/nrm4074 (2015). 26490400 10.1038/nrm4074 PMC4884795

[27] Santana-Farre, R. et al. Influence of neonatal hypothyroidism on hepatic gene expression and lipid metabolism in adulthood. *PLoS One*10.1371/journal.pone.0037386 (2012). 22666351 10.1371/journal.pone.0037386 PMC3354003

[28] Sinha, R., Yen, P.M., Feingold, K.R., Anawalt, B., Blackman, M.R., Boyce, A., Chrousos, G., Corpas, E., de Herder, W.W., Dhatariya, K., Dungan, K., Hofland, J., Kalra, S., Kaltsas, G., Kapoor, N., Koch, C., Kopp, P., Korbonits, M., Kovacs, C.S., Kuohung, W., Laferrère, B., Levy, M., McGee, E.A., McLachlan, R., New, M., Purnell, J., Sahay, R., Shah, A.S., Singer, F., Sperling, M.A., Stratakis, C.A., Trence, D.L. & Wilson, D.P. (2018) Cellular Action of Thyroid Hormone.

[29] Johnson, J. L. & Johnson, L. A. *Homeostasis of Lipid Metabolism in Disorders of the Brain Encyclopedia of Behavioral Neuroscience* 2nd edn, 372–382 (Elsevier, 2022). 10.1016/B978-0-12-819641-0.00146-8.

[30] Sorrenti, S. et al. Iodine: Its role in thyroid hormone biosynthesis and beyond. *Nutrients* **13**(12), 4469. 10.3390/nu13124469 (2021). 34960019 10.3390/nu13124469 PMC8709459

[31] McCord, J. M. The evolution of free radicals and oxidative stress. *Am. J. Med.* **108**, 652–659 (2000). 10856414 10.1016/s0002-9343(00)00412-5

[32] McGill, M. R. & Jaeschke, H. Chapter 4 - Oxidant Stress, Antioxidant Defense, and Liver Injury. in Drug-Induced Liver Disease (Third Edition) (eds. Kaplowitz, N. & DeLeve, L. D.) 71–84 (Academic Press, Boston, 2013). 10.1016/B978-0-12-387817-5.00004-2.

[33] Ahsan, H., Hasan, M. Y. & Ahmad, R. Chapter 16 - Role of free radicals in autoimmune diseases. In *Translational autoimmunity* (ed. Rezaei, N.) (Academic Press, 2022). 10.1016/B978-0-12-822564-6.00016-1.

[34] Lobo, V., Patil, A., Phatak, A. & Chandra, N. Free radicals, antioxidants and functional foods: Impact on human health. *Pharmacogn Rev.* **4**, 118–126. 10.4103/0973-7847.70902 (2010). 22228951 10.4103/0973-7847.70902 PMC3249911

[35] Reddy, V. P. Oxidative stress in health and disease. *Biomedicines* **11**, 2925 (2023). 38001926 10.3390/biomedicines11112925 PMC10669448

[36] Halliwell, B. & Gutteridge, J. M. C. Cellular responses to oxidative stress: adaptation, damage, repair, senescence and death. *Free Radic. Biol. Med.* **4**(1), 187–267 (2007).

[37] Kochman, J., Jakubczyk, K., Bargiel, P. & Janda-Milczarek, K. The influence of oxidative stress on thyroid diseases. *Antioxidants* **10**, 1442 (2021). 34573074 10.3390/antiox10091442 PMC8465820

[38] Sugawara, M. Reactive Oxygen Species and Thyroid Diseases. in Systems Biology of Free Radicals and Antioxidants (ed. Laher, I.) 3521–3538 (Springer, Berlin, Heidelberg, 2014). 10.1007/978-3-642-30018-9_150.

[39] Hubler, M. J. & Kennedy, A. J. Role of lipids in the metabolism and activation of immune cells. *J. Nutr. Biochem.* **34**, 1–7. 10.1016/j.jnutbio.2015.11.002 (2016). 27424223 10.1016/j.jnutbio.2015.11.002 PMC5694687

[40] Cengiz, H., Demirci, T., Varim, C. & Tamer, A. The effect of thyroid autoimmunity on dyslipidemia in patients with euthyroid hashimoto thyroiditis. *Pak. J. Med. Sci.* **37**(5), 1365–1370. 10.12669/pjms.37.5.3883 (2021). 34475913 10.12669/pjms.37.5.3883 PMC8377896

[41] Ahluwalia, K. et al. Lipidomics in understanding pathophysiology and pharmacologic effects in inflammatory diseases: considerations for drug development. *Metabolites* **12**(4), 333. 10.3390/metabo12040333 (2022). 35448520 10.3390/metabo12040333 PMC9030008

[42] Zandl-Lang, M., Plecko, B. & Köfeler, H. Lipidomics—paving the road towards better insight and precision medicine in rare metabolic diseases. *Int. J. Mol. Sci.* **24**, 1709. 10.3390/ijms24021709 (2023). 36675224 10.3390/ijms24021709 PMC9866746

[43] Johnson, J. L. & Johnson, L. A. Homeostasis of Lipid Metabolism in Disorders of the Brain. In *Encyclopedia of Behavioral Neuroscience* 2nd edn (ed. Della Sala, S.) 372–382 (Elsevier, 2022). 10.1016/B978-0-12-819641-0.00146-8.

[44] Li, L., Zhang, M., Men, Y., Wang, W. & Zhang, W. Heavy metals interfere with plasma metabolites. Including lipids and amino acids. in patients with breast cancer. *OncolLett.* **19**, 2925–2933. 10.3892/ol.2020.11402 (2020). 10.3892/ol.2020.11402 PMC7068226 32218848

[45] Franco, J.S., Amaya-Amaya, J. & Anaya, J.M. (2013) Thyroid disease and autoimmune diseases. Autoimmunity: From Bench to Bedside [Internet]. https://www.ncbi.nlm.nih.gov/books/NBK459466/

[46] Huo, Z. et al. Brain and blood metabolome for Alzheimer’s dementia: findings from a targeted metabolomics analysis. *Neurobiol Aging.* **86**, 123–133. 10.1016/j.neurobiolaging.2019.10.014 (2020). 31785839 10.1016/j.neurobiolaging.2019.10.014 PMC6995427

[47] Jonas, J. P. et al. Circulating metabolites as a concept beyond tumor biology determining disease recurrence after resection of colorectal liver metastasis. *HPB* **24**(1), 116–129. 10.1016/j.hpb.2021.06.415 (2022). 34257019 10.1016/j.hpb.2021.06.415

[48] Kuhn, M. et al. Mass spectrometric profiling of cerebrospinal fluid reveals metabolite biomarkers for CNS involvement in varicella - zoster virus reactivation. *J. Neuroinflammation.* **15**(1), 20. 10.12669/pjms.37.5.3883 (2018). 29343258 10.1186/s12974-017-1041-0 PMC5773076

[49] Breier, M. et al. Immediate reduction of serum citrulline but no change of steroid profile after initiation of metformin in individuals with type 2 diabetes. *J. Steroid Biochem. Molecular Biol.* **174**, 114–119. 10.1016/j.jsbmb.2017.08.004 (2017). 10.1016/j.jsbmb.2017.08.004 28801099

[50] Dong, Q. et al. Metabolic signatures elucidate the effect of body mass index on Type 2 diabetes. *Metabolites* **13**(2), 227. 10.3390/metabo13020227 (2023). 36837846 10.3390/metabo13020227 PMC9965667

[51] Quell, J. D. et al. Characterization of bulk phosphatidylcholine compositions in human plasma using side-chain resolving lipidomics. *Metabolites* **9**(6), 109. 10.3390/metabo9060109 (2019). 31181753 10.3390/metabo9060109 PMC6631474

[52] Signorelli, P., Conte, C. & Albi, E. The multiple roles of sphingomyelin in Parkinson’s Disease. *Biomolecules* **11**(9), 1311. 10.3390/biom11091311 (2021). 34572524 10.3390/biom11091311 PMC8469734

[53] Varma, V. R. et al. Brain and blood metabolite signatures of pathology and progression in Alzheimer disease: A targeted metabolomics study. *PLoS Med.* **15**(1), e1002482. 10.1371/journal.pmed.1002482 (2018). 29370177 10.1371/journal.pmed.1002482 PMC5784884

[54] Olsen, A. S. B. & Færgeman, N. J. Sphingolipids: membrane microdomains in brain development function and neurological diseases. *Open Biol.* **7**(5), 170069. 10.1098/rsob.170069 (2017). 28566300 10.1098/rsob.170069 PMC5451547

[55] Podbielska, M., Ariga, T. & Pokryszko-Dragan, A. Sphingolipid players in multiple sclerosis: Their influence on the initiation and course of the disease. *Int. J. Mol. Sci.* **23**, 5330. 10.3390/ijms23105330 (2022). 35628142 10.3390/ijms23105330 PMC9140914

[56] Lee, M., Lee, S. Y. & Bae, Y. S. Functional roles of sphingolipids in immunity and their implication in disease. *Exp Mol Med* **55**, 1110–1130. 10.1038/s12276-023-01018-9 (2023). 37258585 10.1038/s12276-023-01018-9 PMC10318102

[57] Walter, S. et al. Pharmacological inhibition of acid sphingomyelinase ameliorates experimental autoimmune encephalomyelitis. *Neurosignals* **27**, 20–31. 10.33594/000000183 (2019). 31778303 10.33594/000000183

[58] Beckmann, N. et al. Regulation of arthritis severity by the acid sphingomyelinase. *Cell Physiol. Biochem.* **43**, 1460–1471. 10.1159/000481968 (2017). 29035882 10.1159/000481968

[59] Grassi, S., Chiricozzi, E., Mauri, L., Sonnino, S. & Prinetti, A. Sphingolipids and neuronal degeneration in lysosomal storage disorders. *J. Neurochem.* **148**, 600–611. 10.1111/jnc.14540 (2019). 29959861 10.1111/jnc.14540

[60] Carsana, E. V. et al. Massive accumulation of sphingomyelin affects the lysosomal and mitochondria compartments and promotes apoptosis in Niemann-Pick disease type A. *J. Mol. Neurosci.* **72**(7), 1482–1499. 10.1007/s12031-022-02036-4 (2022). 35727525 10.1007/s12031-022-02036-4 PMC9293875

[61] Edsfeldt, A. et al. sphingolipids contribute to human atherosclerotic plaque inflammation. *Arteriosclerosis Trombosis Vascular Biol.*10.1161/ATVBAHA.116.305675 (2016). 10.1161/ATVBAHA.116.305675 27055903

[62] Pan, F. et al. Sphingomyelin is involved in multisite musculoskeletal pain: evidence from metabolomic analysis in 2 independent cohorts. *Pain* **162**(6), 1876–1881. 10.1097/j.pain.0000000000002163 (2021). 33273416 10.1097/j.pain.0000000000002163

[63] Assi, N. et al. Do metabolic signatures mediate the association between lifestyle factors and hepatocellular carcinoma risk? Results from a nested case-control study in EPIC. *Cancer Epidemiol Biomarkers Prev.* **27**(5), 531–540. 10.1158/1055-9965.EPI-17-0649 (2018). 29563134 10.1158/1055-9965.EPI-17-0649 PMC7444360

[64] Meyer, J. J. et al. Blood-based targeted metabolomics discriminate patients with alcoholic liver cirrhosis from those with non-cirrhotic liver damage: An explorative study. *Dig Dis.* **40**(2), 223–231. 10.1159/000516488 (2022). 33866312 10.1159/000516488

[65] Varma, V. R. et al. Brain and blood metabolite signatures of pathology and progression in Alzheimer disease: A targeted metabolomics study. *PLoS Med.* **15**(1), e1002482. 10.1371/journal.pmed.1002482 (2018). 29370177 10.1371/journal.pmed.1002482 PMC5784884

[66] Sokolowska, E. et al. Sphingomyelin profiling in patients with diabetes could be potentially useful as differential diagnostics biomarker: A pilot study. *Adv. Med. Sci.* **67**(2), 250–256. 10.1016/j.advms.2022.06.001 (2022). 35785598 10.1016/j.advms.2022.06.001

[67] Erlic, Z. et al. Targeted metabolomics as a tool in discriminating endocrine from primary hypertension. *J. Clin. Endocrinol. Metab.* **106**(4), 1111–1128. 10.1210/clinem/dgaa954 (2021). 33382876 10.1210/clinem/dgaa954 PMC7993566

[68] Huang, J. et al. Validation of candidate phospholipid biomarkers of chronic kidney disease in hyperglycemic individuals and their organ-specific exploration in leptin receptor-deficient db/db mouse. *Metabolites* **11**(2), 89. 10.3390/metabo11020089 (2021). 33546276 10.3390/metabo11020089 PMC7913334

[69] Ożegowska, K., Plewa, S., Mantaj, U., Pawelczyk, L. & Matysiak, J. Serum metabolomics in PCOS women with different body mass index. *J. Clin. Med.* **10**(13), 2811. 10.3390/jcm10132811 (2021). 34202365 10.3390/jcm10132811 PMC8268990

[70] Vouk, K., Ribič-Pucelj, M., Adamski, J. & Lanišnik Rižner, T. Altered levels of acylcarnitines. phosphatidylcholines. and sphingomyelins in peritoneal fluid from ovarian endometriosis patients. *J. Steroid Biochem. Molecular Biol.* **159**, 60–69. 10.1016/j.jsbmb.2016.02.023 (2016). 10.1016/j.jsbmb.2016.02.023 26921767

[71] Khorasani, E., Mirhafez, S. R. & Niroumand, S. Assessment of the selenium status in hypothyroid children from north east of Iran. *J. Biol. Today’s World* **6**, 21–26 (2017).

[72] Stojsavljević, A. et al. Significance of arsenic and lead in hashimoto’s thyroiditis demonstrated on thyroid tissue, blood, and urine samples. *Environ. Res.* **186**, 109538 (2020). 32334172 10.1016/j.envres.2020.109538

[73] Soujanya, K. V. & Jayadeep, A. P. Obesity-associated biochemical markers of inflammation and the role of grain phytochemicals. *J. Food Biochem.* **46**, e14257 (2022). 35674206 10.1111/jfbc.14257

[74] Saito, Y., Yoshida, Y., Akazawa, T., Takahashi, K. & Niki, E. Cell death caused by selenium deficiency and protective effect of antioxidants. *JBC* **278**(41), 39428–39434 (2003). 10.1074/jbc.M305542200 12888577

[75] Finelli, C. Molecular mechanisms and mediators of hepatotoxicity resulting from an excess of lipids and non-alcoholic fatty liver disease. *Gastrointest. Disord.* **5**, 243–260. 10.3390/gidisord5020020 (2023).

[76] Bień, D., Michalczuk, M., Szkopek, D., Kinsner, M. & Konieczka, P. Changes in lipids metabolism indices as a result of different form of selenium supplementation in chickens. *Sci. Rep.* **12**(1), 13817. 10.1038/s41598-022-18101-2 (2022). 35970995 10.1038/s41598-022-18101-2 PMC9378790

[77] Hasani, M. et al. Effect of selenium supplementation on lipid profile: A systematic review and meta-analysis. *Horm Metab Res.* **50**(10), 715–727. 10.1055/a-0749-6655 (2018). 30312982 10.1055/a-0749-6655

[78] Christensen, K., Werner, M. & Malecki, K. Serum selenium and lipid levels: Associations observed in the National Health and Nutrition Examination Survey (NHANES) 2011–2012, Environmental Research, 140. *ISSN* **76–84**, 0013–9351. 10.1016/j.envres.2015.03.020 (2015). 10.1016/j.envres.2015.03.020 25836721

[79] Bleys, J. et al. Serum selenium and serum lipids in US adults. *Am. J. Clin. Nutr.* **88**, 416–423. 10.1093/ajcn/88.2.416 (2008). 18689378 10.1093/ajcn/88.2.416 PMC2553708

[80] Wasowicz, W., Gromadzinska, J., Rydzynski, K. & Tomczak, J. Selenium status of low-selenium area residents: Polish experience. *Toxicol. Lett.* **137**, 95–101. 10.1016/S0378-4274(02)00383-1 (2003). 12505435 10.1016/s0378-4274(02)00383-1

[81] Rayman, M. The importance of selenium to human health. *Lancet* **356**, 233–241. 10.1016/S0140-6736(00)02490-9 (2000). 10963212 10.1016/S0140-6736(00)02490-9

[82] Janowska, M. et al. An assessment of serum selenium concentration in women with endometrial cancer. *Nutrients, MDPI* **14**(5), 958. 10.3390/nu14050958 (2022). 10.3390/nu14050958 PMC8912795 35267933

[83] Muntau, A. C. et al. Age-related reference values for serum selenium concentrations in infants and children. *Clin Chem.* **48**(3), 555–560. 10.1093/clinchem/48.3.555 (2002). 11861447

[84] Vinceti, M. et al. Selenium speciation in human serum and its implications for epidemiologic research: a cross-sectional study. *J. Trace Elements Med. Biol.* **31**, 1–10. 10.1016/j.jtemb.2015.02.001 (2015). 10.1016/j.jtemb.2015.02.001 26004885

[85] Pyrzynska, K. & Sentkowska, A. selenium species in diabetes mellitus type 2. *Biol. Trace Elem. Res.*10.1007/s12011-023-03900-z (2023). 37880477 10.1007/s12011-023-03900-z PMC11074226

