# Supplementary material for: Preliminary investigations of plasma lipidome and selenium levels in adults with treated hypothyroidism and in healthy individuals without selenium deficiency
Source: Sci Rep. 2024 Nov 25;14:29140. doi: 10.1038/s41598-024-80862-9 (PMC11589578; doi:10.1038/s41598-024-80862-9)
Supplement: Supplementary file 1 — Supplementary Information 1. [file 41598_2024_80862_MOESM1_ESM.pdf]

Table S1: Recovery and precision of the assay.

Certified Reference Material	Concentration [µg/L]		Recovery (%)	Reapetability/ Precision RSD** (%)
	Certified	Measured ± SD*		
EU-H-3	11.60	12.08 ± 0.42	104.17	3.49
EP-H-2	12.00	12.30 ± 0.45	102.50	3.69
Seronorm™ Trace element Serum L2	136.00	129.20±3.70	95.00	2.86

*SD – standard deviation, **RSD – relative standard deviation (RSD = SD*100%/ mean), precision in terms of % RSD for replicate measurements (n = 3) at three different concentrations.
