# Supplementary material for: Preliminary investigations of plasma lipidome and selenium levels in adults with treated hypothyroidism and in healthy individuals without selenium deficiency
Source: Sci Rep. 2024 Nov 25;14:29140. doi: 10.1038/s41598-024-80862-9 (PMC11589578; doi:10.1038/s41598-024-80862-9)
Supplement: Supplementary file 2 — Supplementary Information 2. [file 41598_2024_80862_MOESM2_ESM.pdf]

Table S2. Multiple comparisons of p values (2-tailed) of selenium plasma levels and selected lipid species in three groups: Hashimoto's disease, non-autoimmune hypothyroidism (Hypo-non-Hashimoto), and healthy control. All values are presented.

	Kruskal-Wallis ANOVA by Ranks; Se mcg/L<br>Independent (grouping) variable: Hashimoto 1 Hypo-no-Hashimoto 2 control 3<br>Kruskal-Wallis test: H (2, N= 41) =11,82093 p =,0027			
Depend.:<br>Se mcg/L	Code	Valid<br>N	Sum of<br>Ranks	Mean<br>Rank
1	1	24	459.0000	19.12500
2	2	11	184.0000	16.72727
3	3	6	218.0000	36.33333

Dependent:<br>Se mcg/L	Median Test, Overall Median = 98,8520; Se mcg/L<br>Independent (grouping) variable: Hashimoto 1 Hypo-no-Hashimoto 2 control 3<br>Chi-Square = 7,464899 df = 2 p = ,0239			
	1	2	3	Total
<= Median: observed	14.00000	7.00000	0.00000	21.00000
expected	12.29268	5.63415	3.07317	
obs.-exp.	1.70732	1.36585	-3,07317	
> Median: observed	10.00000	4.00000	6.00000	20.00000
expected	11.70732	5.36585	2.92683	
obs.-exp.	-1,70732	-1,36585	3.07317	
Total: observed	24.00000	11.00000	6.00000	41.00000

	Kruskal-Wallis ANOVA by Ranks; lysoPC a C14:0<br>Independent (grouping) variable: Hashimoto 1 Hypo-no-Hashimoto 2 control 3<br>Kruskal-Wallis test: H (2, N= 41) =2,564029 p =,2775			
Depend.:<br>lysoPC a C14:0	Code	Valid<br>N	Sum of<br>Ranks	Mean<br>Rank
1	1	24	562.5000	23.43750
2	2	11	183.5000	16.68182
3	3	6	115.0000	19.16667

Dependent:<br>lysoPC a C14:0	Median Test, Overall Median = 1,87000; lysoPC a C14:0<br>Independent (grouping) variable: Hashimoto 1 Hypo-no-Hashimoto 2 control 3<br>Chi-Square = 2,128391 df = 2 p = ,3450			
	1	2	3	Total
<= Median: observed	10.00000	7.00000	4.000000	21.00000
expected	12.29268	5.63415	3.073171	
obs.-exp.	-2,29268	1.36585	0.926829	
> Median: observed	14.00000	4.00000	2.000000	20.00000
expected	11.70732	5.36585	2.926829	
obs.-exp.	2.29268	-1,36585	-0,926829	
Total: observed	24.00000	11.00000	6.000000	41.00000

	Kruskal-Wallis ANOVA by Ranks; lysoPC a C16:0<br>Independent (grouping) variable: Hashimoto 1 Hypo-no-Hashimoto 2 control 3<br>Kruskal-Wallis test: H (2, N= 41) =4,717705 p =,0945			
Depend.:<br>lysoPC a C16:0	Code	Valid<br>N	Sum of<br>Ranks	Mean<br>Rank
1	1	24	574.0000	23.91667
2	2	11	159.0000	14.45455
3	3	6	128.0000	21.33333

Dependent: lysoPC a C16:0	Median Test, Overall Median = 121,000; lysoPC a C16:0<br>Independent (grouping) variable: Hashimoto 1 Hypo-no-Hashimoto 2 control 3<br>Chi-Square = 2,916739 df = 2 p = ,2326			
	1	2	3	Total
<= Median: observed	10.00000	8.00000	3.000000	21.00000
expected	12.29268	5.63415	3.073171	
obs.-exp.	-2,29268	2.36585	-0,073171	
> Median: observed	14.00000	3.00000	3.000000	20.00000
expected	11.70732	5.36585	2.926829	
obs.-exp.	2.29268	-2,36585	0.073171	
Total: observed	24.00000	11.00000	6.000000	41.00000

Depend.: lysoPC a C16:1	Kruskal-Wallis ANOVA by Ranks; lysoPC a C16:1<br>Independent (grouping) variable: Hashimoto 1 Hypo-no-Hashimoto 2 control 3<br>Kruskal-Wallis test: H (2, N= 41) =2,918812 p =,2324			
	Code	Valid N	Sum of Ranks	Mean Rank
1	1	24	548.5000	22.85417
2	2	11	173.0000	15.72727
3	3	6	139.5000	23.25000

Dependent: lysoPC a C16:1	Median Test, Overall Median = 2,71000; lysoPC a C16:1<br>Independent (grouping) variable: Hashimoto 1 Hypo-no-Hashimoto 2 control 3<br>Chi-Square = 2,916739 df = 2 p = ,2326			
	1	2	3	Total
<= Median: observed	10.00000	8.00000	3.000000	21.00000
expected	12.29268	5.63415	3.073171	
obs.-exp.	-2,29268	2.36585	-0,073171	
> Median: observed	14.00000	3.00000	3.000000	20.00000
expected	11.70732	5.36585	2.926829	
obs.-exp.	2.29268	-2,36585	0.073171	
Total: observed	24.00000	11.00000	6.000000	41.00000

Depend.: lysoPC a C17:0	Kruskal-Wallis ANOVA by Ranks; lysoPC a C17:0<br>Independent (grouping) variable: Hashimoto 1 Hypo-no-Hashimoto 2 control 3<br>Kruskal-Wallis test: H (2, N= 41) =1,625275 p =,4437			
	Code	Valid N	Sum of Ranks	Mean Rank
1	1	24	552.0000	23.00000
2	2	11	197.5000	17.95455
3	3	6	111.5000	18.58333

Dependent: lysoPC a C17:0	Median Test, Overall Median = 1,95000; lysoPC a C17:0<br>Independent (grouping) variable: Hashimoto 1 Hypo-no-Hashimoto 2 control 3<br>Chi-Square = 2,128391 df = 2 p = ,3450			
	1	2	3	Total
<= Median: observed	10.00000	7.00000	4.000000	21.00000
expected	12.29268	5.63415	3.073171	
obs.-exp.	-2,29268	1.36585	0.926829	
> Median: observed	14.00000	4.00000	2.000000	20.00000
expected	11.70732	5.36585	2.926829	
obs.-exp.	2.29268	-1,36585	-0,926829	
Total: observed	24.00000	11.00000	6.000000	41.00000

Depend.: lysoPC a C18:0	Kruskal-Wallis ANOVA by Ranks; lysoPC a C18:0<br>Independent (grouping) variable: Hashimoto 1 Hypo-no-Hashimoto 2 control 3<br>Kruskal-Wallis test: H (2, N= 41) =3,782203 p =,1509			
	Code	Valid N	Sum of Ranks	Mean Rank
1	1	24	577.0000	24.04167
2	2	11	178.5000	16.22727
3	3	6	105.5000	17.58333

Dependent: lysoPC a C18:0	Median Test, Overall Median = 36,5000; lysoPC a C18:0<br>Independent (grouping) variable: Hashimoto 1 Hypo-no-Hashimoto 2 control 3<br>Chi-Square = 2,128391 df = 2 p = ,3450			
	1	2	3	Total
<= Median: observed	10.00000	7.00000	4.000000	21.00000
expected	12.29268	5.63415	3.073171	
obs.-exp.	-2,29268	1.36585	0.926829	
> Median: observed	14.00000	4.00000	2.000000	20.00000
expected	11.70732	5.36585	2.926829	
obs.-exp.	2.29268	-1,36585	-0,926829	
Total: observed	24.00000	11.00000	6.000000	41.00000

Depend.: lysoPC a C18:1	Kruskal-Wallis ANOVA by Ranks; lysoPC a C18:1<br>Independent (grouping) variable: Hashimoto 1 Hypo-no-Hashimoto 2 control 3<br>Kruskal-Wallis test: H (2, N= 41) =5,245047 p =,0726			
	Code	Valid N	Sum of Ranks	Mean Rank
1	1	24	560.5000	23.35417
2	2	11	153.5000	13.95455
3	3	6	147.0000	24.50000

Dependent: lysoPC a C18:1	Median Test, Overall Median = 29,1000; lysoPC a C18:1<br>Independent (grouping) variable: Hashimoto 1 Hypo-no-Hashimoto 2 control 3<br>Chi-Square = 3,083505 df = 2 p = ,2140			
	1	2	3	Total
<= Median: observed	11.00000	8.00000	2.00000	21.00000
expected	12.29268	5.63415	3.07317	
obs.-exp.	-1,29268	2.36585	-1,07317	
> Median: observed	13.00000	3.00000	4.00000	20.00000
expected	11.70732	5.36585	2.92683	
obs.-exp.	1.29268	-2,36585	1.07317	
Total: observed	24.00000	11.00000	6.00000	41.00000

Depend.: lysoPC a C18:2	Kruskal-Wallis ANOVA by Ranks; lysoPC a C18:2<br>Independent (grouping) variable: Hashimoto 1 Hypo-no-Hashimoto 2 control 3<br>Kruskal-Wallis test: H (2, N= 41) =5,742201 p =,0566			
	Code	Valid N	Sum of Ranks	Mean Rank
1	1	24	537.0000	22.37500
2	2	11	157.0000	14.27273
3	3	6	167.0000	27.83333

Dependent: lysoPC a C18:2	Median Test, Overall Median = 38,2000; lysoPC a C18:2<br>Independent (grouping) variable: Hashimoto 1 Hypo-no-Hashimoto 2 control 3<br>Chi-Square = 7,267812 df = 2 p = ,0264			
	1	2	3	Total
<= Median: observed	11.00000	9.00000	1.00000	21.00000
expected	12.29268	5.63415	3.07317	
obs.-exp.	-1,29268	3.36585	-2,07317	
> Median: observed	13.00000	2.00000	5.00000	20.00000
expected	11.70732	5.36585	2.92683	
obs.-exp.	1.29268	-3,36585	2.07317	
Total: observed	24.00000	11.00000	6.00000	41.00000

Depend.: lysoPC a C20:3	Kruskal-Wallis ANOVA by Ranks; lysoPC a C20:3<br>Independent (grouping) variable: Hashimoto 1 Hypo-no-Hashimoto 2 control 3<br>Kruskal-Wallis test: H (2, N= 41) =6,675612 p =,0355			
	Code	Valid N	Sum of Ranks	Mean Rank
1	1	24	568.5000	23.68750
2	2	11	143.5000	13.04545
3	3	6	149.0000	24.83333

Dependent: lysoPC a C20:3	Median Test, Overall Median = 2,38000; lysoPC a C20:3<br>Independent (grouping) variable: Hashimoto 1 Hypo-no-Hashimoto 2 control 3<br>Chi-Square = 3,083505 df = 2 p = ,2140			
	1	2	3	Total
<= Median: observed	11.00000	8.00000	2.00000	21.00000
expected	12.29268	5.63415	3.07317	
obs.-exp.	-1,29268	2.36585	-1,07317	
> Median: observed	13.00000	3.00000	4.00000	20.00000
expected	11.70732	5.36585	2.92683	
obs.-exp.	1.29268	-2,36585	1.07317	
Total: observed	24.00000	11.00000	6.00000	41.00000

Depend.: lysoPC a C20:4	Kruskal-Wallis ANOVA by Ranks; lysoPC a C20:4<br>Independent (grouping) variable: Hashimoto 1 Hypo-no-Hashimoto 2 control 3<br>Kruskal-Wallis test: H (2, N= 41) =2,722178 p =,2564			
	Code	Valid N	Sum of Ranks	Mean Rank
1	1	24	551.0000	22.95833
2	2	11	175.0000	15.90909
3	3	6	135.0000	22.50000

Dependent: lysoPC a C20:4	Median Test, Overall Median = 7,32000; lysoPC a C20:4<br>Independent (grouping) variable: Hashimoto 1 Hypo-no-Hashimoto 2 control 3<br>Chi-Square = ,9610299 df = 2 p = ,6185			
	1	2	3	Total
<= Median: observed	11.00000	7.00000	3.000000	21.00000
expected	12.29268	5.63415	3.073171	
obs.-exp.	-1,29268	1.36585	-0,073171	
> Median: observed	13.00000	4.00000	3.000000	20.00000
expected	11.70732	5.36585	2.926829	
obs.-exp.	1.29268	-1,36585	0.073171	
Total: observed	24.00000	11.00000	6.000000	41.00000

Depend.: lysoPC a C24:0	Kruskal-Wallis ANOVA by Ranks; lysoPC a C24:0<br>Independent (grouping) variable: Hashimoto 1 Hypo-no-Hashimoto 2 control 3<br>Kruskal-Wallis test: H (2, N= 41) =2,188112 p =,3349			
	Code	Valid N	Sum of Ranks	Mean Rank
1	1	24	460.0000	19.16667
2	2	11	281.0000	25.54545
3	3	6	120.0000	20.00000

Dependent: lysoPC a C24:0	Median Test, Overall Median = ,203000; lysoPC a C24:0 Independent (grouping) variable: Hashimoto 1 Hypo-no-Hashimoto 2 control 3 Chi-Square = 3,750568 df = 2 p = ,1533			
	1	2	3	Total
<= Median: observed	15.00000	3.00000	3.000000	21.00000
expected	12.29268	5.63415	3.073171	
obs.-exp.	2.70732	-2,63415	-0,073171	
> Median: observed	9.00000	8.00000	3.000000	20.00000
expected	11.70732	5.36585	2.926829	
obs.-exp.	-2,70732	2.63415	0.073171	
Total: observed	24.00000	11.00000	6.000000	41.00000

Depend.: lysoPC a C26:0	Kruskal-Wallis ANOVA by Ranks; lysoPC a C26:0 Independent (grouping) variable: Hashimoto 1 Hypo-no-Hashimoto 2 control 3 Kruskal-Wallis test: H (2, N= 41) =1,321587 p =,5164			
	Code	Valid N	Sum of Ranks	Mean Rank
1	1	24	471.0000	19.62500
2	2	11	270.0000	24.54545
3	3	6	120.0000	20.00000

Dependent: lysoPC a C26:0	Median Test, Overall Median = ,275000; lysoPC a C26:0 Independent (grouping) variable: Hashimoto 1 Hypo-no-Hashimoto 2 control 3 Chi-Square = ,2333243 df = 2 p = ,8899			
	1	2	3	Total
<= Median: observed	13.00000	5.00000	3.000000	21.00000
expected	12.29268	5.63415	3.073171	
obs.-exp.	0.70732	-0,63415	-0,073171	
> Median: observed	11.00000	6.00000	3.000000	20.00000
expected	11.70732	5.36585	2.926829	
obs.-exp.	-0,70732	0.63415	0.073171	
Total: observed	24.00000	11.00000	6.000000	41.00000

Depend.: lysoPC a C26:1	Kruskal-Wallis ANOVA by Ranks; lysoPC a C26:1 Independent (grouping) variable: Hashimoto 1 Hypo-no-Hashimoto 2 control 3 Kruskal-Wallis test: H (2, N= 41) =,8012846 p =,6699			
	Code	Valid N	Sum of Ranks	Mean Rank
1	1	24	473.0000	19.70833
2	2	11	259.5000	23.59091
3	3	6	128.5000	21.41667

Dependent: lysoPC a C26:1	Median Test, Overall Median = ,153000; lysoPC a C26:1<br>Independent (grouping) variable: Hashimoto 1 Hypo-no-Hashimoto 2 control 3<br>Chi-Square = 1,461328 df = 2 p = ,4816			
	1	2	3	Total
<= Median: observed	14.00000	4.00000	3.000000	21.00000
expected	12.29268	5.63415	3.073171	
obs.-exp.	1.70732	-1,63415	-0,073171	
> Median: observed	10.00000	7.00000	3.000000	20.00000
expected	11.70732	5.36585	2.926829	
obs.-exp.	-1,70732	1.63415	0.073171	
Total: observed	24.00000	11.00000	6.000000	41.00000

Depend.: lysoPC a C28:0	Kruskal-Wallis ANOVA by Ranks; lysoPC a C28:0<br>Independent (grouping) variable: Hashimoto 1 Hypo-no-Hashimoto 2 control 3<br>Kruskal-Wallis test: H (2, N= 41) =1,084261 p =,5815			
	Code	Valid N	Sum of Ranks	Mean Rank
1	1	24	486.0000	20.25000
2	2	11	264.5000	24.04545
3	3	6	110.5000	18.41667

Dependent: lysoPC a C28:0	Median Test, Overall Median = ,250000; lysoPC a C28:0<br>Independent (grouping) variable: Hashimoto 1 Hypo-no-Hashimoto 2 control 3<br>Chi-Square = ,2333243 df = 2 p = ,8899			
	1	2	3	Total
<= Median: observed	13.00000	5.00000	3.000000	21.00000
expected	12.29268	5.63415	3.073171	
obs.-exp.	0.70732	-0,63415	-0,073171	
> Median: observed	11.00000	6.00000	3.000000	20.00000
expected	11.70732	5.36585	2.926829	
obs.-exp.	-0,70732	0.63415	0.073171	
Total: observed	24.00000	11.00000	6.000000	41.00000

Depend.: lysoPC a C28:1	Kruskal-Wallis ANOVA by Ranks; lysoPC a C28:1<br>Independent (grouping) variable: Hashimoto 1 Hypo-no-Hashimoto 2 control 3<br>Kruskal-Wallis test: H (2, N= 41) =1,360296 p =,5065			
	Code	Valid N	Sum of Ranks	Mean Rank
1	1	24	488.0000	20.33333
2	2	11	267.0000	24.27273
3	3	6	106.0000	17.66667

Dependent: lysoPC a C28:1	Median Test, Overall Median = ,394000; lysoPC a C28:1<br>Independent (grouping) variable: Hashimoto 1 Hypo-no-Hashimoto 2 control 3<br>Chi-Square = ,7336219 df = 2 p = ,6929			
	1	2	3	Total
<= Median: observed	12.00000	5.00000	4.000000	21.00000
expected	12.29268	5.63415	3.073171	
obs.-exp.	-0,29268	-0,63415	0.926829	
> Median: observed	12.00000	6.00000	2.000000	20.00000
expected	11.70732	5.36585	2.926829	
obs.-exp.	0.29268	0.63415	-0,926829	
Total: observed	24.00000	11.00000	6.000000	41.00000

Depend.: PC aa C24:0	Kruskal-Wallis ANOVA by Ranks; PC aa C24:0<br>Independent (grouping) variable: Hashimoto 1 Hypo-no-Hashimoto 2 control 3<br>Kruskal-Wallis test: H (2, N= 41) =6,544281 p =,0379			
	Code	Valid N	Sum of Ranks	Mean Rank
1	1	24	573.0000	23.87500
2	2	11	226.0000	20.54545
3	3	6	62.0000	10.33333

Dependent: PC aa C24:0	Median Test, Overall Median = ,187000; PC aa C24:0<br>Independent (grouping) variable: Hashimoto 1 Hypo-no-Hashimoto 2 control 3<br>Chi-Square = 3,401876 df = 2 p = ,1825			
	1	2	3	Total
<= Median: observed	10.00000	6.00000	5.00000	21.00000
expected	12.29268	5.63415	3.07317	
obs.-exp.	-2,29268	0.36585	1.92683	
> Median: observed	14.00000	5.00000	1.00000	20.00000
expected	11.70732	5.36585	2.92683	
obs.-exp.	2.29268	-0,36585	-1,92683	
Total: observed	24.00000	11.00000	6.00000	41.00000

Depend.: PC aa C26:0	Kruskal-Wallis ANOVA by Ranks; PC aa C26:0<br>Independent (grouping) variable: Hashimoto 1 Hypo-no-Hashimoto 2 control 3<br>Kruskal-Wallis test: H (2, N= 41) =15,82482 p =,0004			
	Code	Valid N	Sum of Ranks	Mean Rank
1	1	24	620.5000	25.85417
2	2	11	197.5000	17.95455
3	3	6	43.0000	7.16667

Dependent: PC aa C26:0	Median Test, Overall Median = 101,000; PC aa C26:0<br>Independent (grouping) variable: Hashimoto 1 Hypo-no-Hashimoto 2 control 3<br>Chi-Square = 0,000000 df = 2 p = 1,000			
	1	2	3	Total
<= Median: observed	24.00000	11.00000	6.000000	41.00000
expected	24.00000	11.00000	6.000000	
obs.-exp.	0.00000	0.00000	0.000000	
> Median: observed	0.00000	0.00000	0.000000	0.00000
expected	0.00000	0.00000	0.000000	
obs.-exp.	0.00000	0.00000	0.000000	
Total: observed	24.00000	11.00000	6.000000	41.00000

Depend.: PC aa C28:1	Kruskal-Wallis ANOVA by Ranks; PC aa C28:1<br>Independent (grouping) variable: Hashimoto 1 Hypo-no-Hashimoto 2 control 3<br>Kruskal-Wallis test: H (2, N= 41) =2,799776 p =,2466			
	Code	Valid N	Sum of Ranks	Mean Rank
1	1	24	551.0000	22.95833
2	2	11	227.0000	20.63636
3	3	6	83.0000	13.83333

Dependent: PC aa C28:1	Median Test, Overall Median = 2,60000; PC aa C28:1<br>Independent (grouping) variable: Hashimoto 1 Hypo-no-Hashimoto 2 control 3<br>Chi-Square = ,9003878 df = 2 p = ,6375			
	1	2	3	Total
<= Median: observed	11.00000	6.00000	4.000000	21.00000
expected	12.29268	5.63415	3.073171	
obs.-exp.	-1,29268	0.36585	0.926829	
> Median: observed	13.00000	5.00000	2.000000	20.00000
expected	11.70732	5.36585	2.926829	
obs.-exp.	1.29268	-0,36585	-0,926829	
Total: observed	24.00000	11.00000	6.000000	41.00000

Depend.: PC aa C30:0	Kruskal-Wallis ANOVA by Ranks; PC aa C30:0<br>Independent (grouping) variable: Hashimoto 1 Hypo-no-Hashimoto 2 control 3<br>Kruskal-Wallis test: H (2, N= 41) =4,849983 p =,0885			
	Code	Valid N	Sum of Ranks	Mean Rank
1	1	24	567.5000	23.64583
2	2	11	223.5000	20.31818
3	3	6	70.0000	11.66667

Dependent: PC aa C30:0	Median Test, Overall Median = 3,32000; PC aa C30:0<br>Independent (grouping) variable: Hashimoto 1 Hypo-no-Hashimoto 2 control 3<br>Chi-Square = 3,401876 df = 2 p = ,1825			
	1	2	3	Total
<= Median: observed	10.00000	6.00000	5.00000	21.00000
expected	12.29268	5.63415	3.07317	
obs.-exp.	-2,29268	0.36585	1.92683	
> Median: observed	14.00000	5.00000	1.00000	20.00000
expected	11.70732	5.36585	2.92683	
obs.-exp.	2.29268	-0,36585	-1,92683	
Total: observed	24.00000	11.00000	6.00000	41.00000

Depend.: PC aa C30:2	Kruskal-Wallis ANOVA by Ranks; PC aa C30:2<br>Independent (grouping) variable: Hashimoto 1 Hypo-no-Hashimoto 2 control 3<br>Kruskal-Wallis test: H (2, N= 41) =0,000000 p =1,000			
	Code	Valid N	Sum of Ranks	Mean Rank
1	1	24	504.0000	21.00000
2	2	11	231.0000	21.00000
3	3	6	126.0000	21.00000

Dependent: PC aa C30:2	Median Test, Overall Median = 101,000; PC aa C30:2<br>Independent (grouping) variable: Hashimoto 1 Hypo-no-Hashimoto 2 control 3<br>Chi-Square = 0,000000 df = 2 p = 1,000			
	1	2	3	Total
<= Median: observed	24.00000	11.00000	6.000000	41.00000
expected	24.00000	11.00000	6.000000	
obs.-exp.	0.00000	0.00000	0.000000	
> Median: observed	0.00000	0.00000	0.000000	0.00000
expected	0.00000	0.00000	0.000000	
obs.-exp.	0.00000	0.00000	0.000000	
Total: observed	24.00000	11.00000	6.000000	41.00000

Depend.: PC aa C32:0	Kruskal-Wallis ANOVA by Ranks; PC aa C32:0<br>Independent (grouping) variable: Hashimoto 1 Hypo-no-Hashimoto 2 control 3<br>Kruskal-Wallis test: H (2, N= 41) =4,083830 p =,1298			
	Code	Valid N	Sum of Ranks	Mean Rank
1	1	24	571.0000	23.79167
2	2	11	210.5000	19.13636
3	3	6	79.5000	13.25000

Dependent: PC aa C32:0	Median Test, Overall Median = 10,8000; PC aa C32:0<br>Independent (grouping) variable: Hashimoto 1 Hypo-no-Hashimoto 2 control 3<br>Chi-Square = 3,401876 df = 2 p = ,1825			
	1	2	3	Total
<= Median: observed	10.00000	6.00000	5.00000	21.00000
expected	12.29268	5.63415	3.07317	
obs.-exp.	-2,29268	0.36585	1.92683	
> Median: observed	14.00000	5.00000	1.00000	20.00000
expected	11.70732	5.36585	2.92683	
obs.-exp.	2.29268	-0,36585	-1,92683	
Total: observed	24.00000	11.00000	6.00000	41.00000

Depend.: PC aa C32:1	Kruskal-Wallis ANOVA by Ranks; PC aa C32:1<br>Independent (grouping) variable: Hashimoto 1 Hypo-no-Hashimoto 2 control 3<br>Kruskal-Wallis test: H (2, N= 41) =,9301031 p =,6281			
	Code	Valid N	Sum of Ranks	Mean Rank
1	1	24	538.5000	22.43750
2	2	11	216.0000	19.63636
3	3	6	106.5000	17.75000

Dependent: PC aa C32:1	Median Test, Overall Median = 11,2000; PC aa C32:1<br>Independent (grouping) variable: Hashimoto 1 Hypo-no-Hashimoto 2 control 3<br>Chi-Square = ,9003878 df = 2 p = ,6375			
	1	2	3	Total
<= Median: observed	11.00000	6.00000	4.000000	21.00000
expected	12.29268	5.63415	3.073171	
obs.-exp.	-1,29268	0.36585	0.926829	
> Median: observed	13.00000	5.00000	2.000000	20.00000
expected	11.70732	5.36585	2.926829	
obs.-exp.	1.29268	-0,36585	-0,926829	
Total: observed	24.00000	11.00000	6.000000	41.00000

Depend.: PC aa C32:2	Kruskal-Wallis ANOVA by Ranks; PC aa C32:2<br>Independent (grouping) variable: Hashimoto 1 Hypo-no-Hashimoto 2 control 3<br>Kruskal-Wallis test: H (2, N= 41) =1,985590 p =,3705			
	Code	Valid N	Sum of Ranks	Mean Rank
1	1	24	556.0000	23.16667
2	2	11	204.5000	18.59091
3	3	6	100.5000	16.75000

Dependent: PC aa C32:2	Median Test, Overall Median = 2,27000; PC aa C32:2<br>Independent (grouping) variable: Hashimoto 1 Hypo-no-Hashimoto 2 control 3<br>Chi-Square = 2,128391 df = 2 p = ,3450			
	1	2	3	Total
<= Median: observed	10.00000	7.00000	4.000000	21.00000
expected	12.29268	5.63415	3.073171	
obs.-exp.	-2,29268	1.36585	0.926829	
> Median: observed	14.00000	4.00000	2.000000	20.00000
expected	11.70732	5.36585	2.926829	
obs.-exp.	2.29268	-1,36585	-0,926829	
Total: observed	24.00000	11.00000	6.000000	41.00000

Depend.: PC aa C32:3	Kruskal-Wallis ANOVA by Ranks; PC aa C32:3<br>Independent (grouping) variable: Hashimoto 1 Hypo-no-Hashimoto 2 control 3<br>Kruskal-Wallis test: H (2, N= 41) =1,406820 p =,4949			
	Code	Valid N	Sum of Ranks	Mean Rank
1	1	24	548.5000	22.85417
2	2	11	205.5000	18.68182
3	3	6	107.0000	17.83333

Dependent: PC aa C32:3	Median Test, Overall Median = ,330000; PC aa C32:3<br>Independent (grouping) variable: Hashimoto 1 Hypo-no-Hashimoto 2 control 3<br>Chi-Square = 2,128391 df = 2 p = ,3450			
	1	2	3	Total
<= Median: observed	10.00000	7.00000	4.000000	21.00000
expected	12.29268	5.63415	3.073171	
obs.-exp.	-2,29268	1.36585	0.926829	
> Median: observed	14.00000	4.00000	2.000000	20.00000
expected	11.70732	5.36585	2.926829	
obs.-exp.	2.29268	-1,36585	-0,926829	
Total: observed	24.00000	11.00000	6.000000	41.00000

Depend.: PC aa C34:1	Kruskal-Wallis ANOVA by Ranks; PC aa C34:1<br>Independent (grouping) variable: Hashimoto 1 Hypo-no-Hashimoto 2 control 3<br>Kruskal-Wallis test: H (2, N= 41) =1,163256 p =,5590			
	Code	Valid N	Sum of Ranks	Mean Rank
1	1	24	544.5000	22.68750
2	2	11	202.0000	18.36364
3	3	6	114.5000	19.08333

Dependent: PC aa C34:1	Median Test, Overall Median = 188,000; PC aa C34:1<br>Independent (grouping) variable: Hashimoto 1 Hypo-no-Hashimoto 2 control 3<br>Chi-Square = ,9610299 df = 2 p = ,6185			
	1	2	3	Total
<= Median: observed	11.00000	7.00000	3.000000	21.00000
expected	12.29268	5.63415	3.073171	
obs.-exp.	-1,29268	1.36585	-0,073171	
> Median: observed	13.00000	4.00000	3.000000	20.00000
expected	11.70732	5.36585	2.926829	
obs.-exp.	1.29268	-1,36585	0.073171	
Total: observed	24.00000	11.00000	6.000000	41.00000

Depend.: PC aa C34:2	Kruskal-Wallis ANOVA by Ranks; PC aa C34:2<br>Independent (grouping) variable: Hashimoto 1 Hypo-no-Hashimoto 2 control 3<br>Kruskal-Wallis test: H (2, N= 41) =3,370050 p =,1854			
	Code	Valid N	Sum of Ranks	Mean Rank
1	1	24	572.5000	23.85417
2	2	11	193.5000	17.59091
3	3	6	95.0000	15.83333

Dependent: PC aa C34:2	Median Test, Overall Median = 400,000; PC aa C34:2<br>Independent (grouping) variable: Hashimoto 1 Hypo-no-Hashimoto 2 control 3<br>Chi-Square = 4,417632 df = 2 p = ,1098			
	1	2	3	Total
<= Median: observed	9.00000	8.00000	4.000000	21.00000
expected	12.29268	5.63415	3.073171	
obs.-exp.	-3,29268	2.36585	0.926829	
> Median: observed	15.00000	3.00000	2.000000	20.00000
expected	11.70732	5.36585	2.926829	
obs.-exp.	3.29268	-2,36585	-0,926829	
Total: observed	24.00000	11.00000	6.000000	41.00000

Depend.: PC aa C34:3	Kruskal-Wallis ANOVA by Ranks; PC aa C34:3<br>Independent (grouping) variable: Hashimoto 1 Hypo-no-Hashimoto 2 control 3<br>Kruskal-Wallis test: H (2, N= 41) =1,430817 p =,4890			
	Code	Valid N	Sum of Ranks	Mean Rank
1	1	24	547.5000	22.81250
2	2	11	210.5000	19.13636
3	3	6	103.0000	17.16667

Dependent: PC aa C34:3	Median Test, Overall Median = 12,0000; PC aa C34:3<br>Independent (grouping) variable: Hashimoto 1 Hypo-no-Hashimoto 2 control 3<br>Chi-Square = 4,417632 df = 2 p = ,1098			
	1	2	3	Total
<= Median: observed	9.00000	8.00000	4.000000	21.00000
expected	12.29268	5.63415	3.073171	
obs.-exp.	-3,29268	2.36585	0.926829	
> Median: observed	15.00000	3.00000	2.000000	20.00000
expected	11.70732	5.36585	2.926829	
obs.-exp.	3.29268	-2,36585	-0,926829	
Total: observed	24.00000	11.00000	6.000000	41.00000

Depend.: PC aa C34:4	Kruskal-Wallis ANOVA by Ranks; PC aa C34:4<br>Independent (grouping) variable: Hashimoto 1 Hypo-no-Hashimoto 2 control 3<br>Kruskal-Wallis test: H (2, N= 41) =2,941642 p =,2297			
	Code	Valid N	Sum of Ranks	Mean Rank
1	1	24	567.5000	23.64583
2	2	11	198.0000	18.00000
3	3	6	95.5000	15.91667

Dependent: PC aa C34:4	Median Test, Overall Median = 1,15000; PC aa C34:4<br>Independent (grouping) variable: Hashimoto 1 Hypo-no-Hashimoto 2 control 3<br>Chi-Square = 4,963411 df = 2 p = ,0836			
	1	2	3	Total
<= Median: observed	9.00000	7.00000	5.00000	21.00000
expected	12.29268	5.63415	3.07317	
obs.-exp.	-3,29268	1.36585	1.92683	
> Median: observed	15.00000	4.00000	1.00000	20.00000
expected	11.70732	5.36585	2.92683	
obs.-exp.	3.29268	-1,36585	-1,92683	
Total: observed	24.00000	11.00000	6.00000	41.00000

Depend.: PC aa C36:0	Kruskal-Wallis ANOVA by Ranks; PC aa C36:0<br>Independent (grouping) variable: Hashimoto 1 Hypo-no-Hashimoto 2 control 3<br>Kruskal-Wallis test: H (2, N= 41) =4,692229 p =,0957			
	Code	Valid N	Sum of Ranks	Mean Rank
1	1	24	569.0000	23.70833
2	2	11	220.0000	20.00000
3	3	6	72.0000	12.00000

Dependent:<br>PC aa C36:0	Median Test, Overall Median = ,847000; PC aa C36:0<br>Independent (grouping) variable: Hashimoto 1 Hypo-no-Hashimoto 2 control 3<br>Chi-Square = 3,401876 df = 2 p = ,1825			
	1	2	3	Total
<= Median: observed	10.00000	6.00000	5.00000	21.00000
expected	12.29268	5.63415	3.07317	
obs.-exp.	-2,29268	0.36585	1.92683	
> Median: observed	14.00000	5.00000	1.00000	20.00000
expected	11.70732	5.36585	2.92683	
obs.-exp.	2.29268	-0,36585	-1,92683	
Total: observed	24.00000	11.00000	6.00000	41.00000

Depend.:<br>PC aa C36:1	Kruskal-Wallis ANOVA by Ranks; PC aa C36:1<br>Independent (grouping) variable: Hashimoto 1 Hypo-no-Hashimoto 2 control 3<br>Kruskal-Wallis test: H (2, N= 41) =2,468280 p =,2911			
	Code	Valid N	Sum of Ranks	Mean Rank
1	1	24	561.5000	23.39583
2	2	11	203.0000	18.45455
3	3	6	96.5000	16.08333

Dependent:<br>PC aa C36:1	Median Test, Overall Median = 36,6000; PC aa C36:1<br>Independent (grouping) variable: Hashimoto 1 Hypo-no-Hashimoto 2 control 3<br>Chi-Square = ,9003878 df = 2 p = ,6375			
	1	2	3	Total
<= Median: observed	11.00000	6.00000	4.000000	21.00000
expected	12.29268	5.63415	3.073171	
obs.-exp.	-1,29268	0.36585	0.926829	
> Median: observed	13.00000	5.00000	2.000000	20.00000
expected	11.70732	5.36585	2.926829	
obs.-exp.	1.29268	-0,36585	-0,926829	
Total: observed	24.00000	11.00000	6.000000	41.00000

Depend.:<br>PC aa C36:2	Kruskal-Wallis ANOVA by Ranks; PC aa C36:2<br>Independent (grouping) variable: Hashimoto 1 Hypo-no-Hashimoto 2 control 3<br>Kruskal-Wallis test: H (2, N= 41) =2,805639 p =,2459			
	Code	Valid N	Sum of Ranks	Mean Rank
1	1	24	565.5000	23.56250
2	2	11	200.5000	18.22727
3	3	6	95.0000	15.83333

Dependent: PC aa C36:2	Median Test, Overall Median = 199,000; PC aa C36:2 Chi-Square = 7,586183 df = 2 p = ,0225			
	1	2	3	Total
<= Median: observed	8.00000	8.00000	5.00000	21.00000
expected	12.29268	5.63415	3.07317	
obs.-exp.	-4,29268	2.36585	1.92683	
> Median: observed	16.00000	3.00000	1.00000	20.00000
expected	11.70732	5.36585	2.92683	
obs.-exp.	4.29268	-2,36585	-1,92683	
Total: observed	24.00000	11.00000	6.00000	41.00000

Depend.: PC aa C36:3	Kruskal-Wallis ANOVA by Ranks; PC aa C36:3<br>Independent (grouping) variable: Hashimoto 1 Hypo-no-Hashimoto 2 control 3<br>Kruskal-Wallis test: H (2, N= 41) =1,187274 p =,5523			
	Code	Valid N	Sum of Ranks	Mean Rank
1	1	24	543.0000	22.62500
2	2	11	214.0000	19.45455
3	3	6	104.0000	17.33333

Dependent: PC aa C36:3	Median Test, Overall Median = 97,1000; PC aa C36:3<br>Independent (grouping) variable: Hashimoto 1 Hypo-no-Hashimoto 2 control 3<br>Chi-Square = ,9003878 df = 2 p = ,6375			
	1	2	3	Total
<= Median: observed	11.00000	6.00000	4.000000	21.00000
expected	12.29268	5.63415	3.073171	
obs.-exp.	-1,29268	0.36585	0.926829	
> Median: observed	13.00000	5.00000	2.000000	20.00000
expected	11.70732	5.36585	2.926829	
obs.-exp.	1.29268	-0,36585	-0,926829	
Total: observed	24.00000	11.00000	6.000000	41.00000

Depend.: PC aa C36:4	Kruskal-Wallis ANOVA by Ranks; PC aa C36:4<br>Independent (grouping) variable: Hashimoto 1 Hypo-no-Hashimoto 2 control 3<br>Kruskal-Wallis test: H (2, N= 41) =2,891786 p =,2355			
	Code	Valid N	Sum of Ranks	Mean Rank
1	1	24	564.0000	23.50000
2	2	11	206.5000	18.77273
3	3	6	90.5000	15.08333

Dependent: PC aa C36:4	Median Test, Overall Median = 153,000; PC aa C36:4<br>Independent (grouping) variable: Hashimoto 1 Hypo-no-Hashimoto 2 control 3<br>Chi-Square = ,9003878 df = 2 p = ,6375			
	1	2	3	Total
<= Median: observed	11.00000	6.00000	4.000000	21.00000
expected	12.29268	5.63415	3.073171	
obs.-exp.	-1,29268	0.36585	0.926829	
> Median: observed	13.00000	5.00000	2.000000	20.00000
expected	11.70732	5.36585	2.926829	
obs.-exp.	1.29268	-0,36585	-0,926829	
Total: observed	24.00000	11.00000	6.000000	41.00000

Depend.: PC aa C36:5	Kruskal-Wallis ANOVA by Ranks; PC aa C36:5<br>Independent (grouping) variable: Hashimoto 1 Hypo-no-Hashimoto 2 control 3<br>Kruskal-Wallis test: H (2, N= 41) =,2505579 p =,8823			
	Code	Valid N	Sum of Ranks	Mean Rank
1	1	24	490.0000	20.41667
2	2	11	248.0000	22.54545
3	3	6	123.0000	20.50000

Dependent: PC aa C36:5	Median Test, Overall Median = 16,9000; PC aa C36:5<br>Independent (grouping) variable: Hashimoto 1 Hypo-no-Hashimoto 2 control 3<br>Chi-Square = ,0665584 df = 2 p = ,9673			
	1	2	3	Total
<= Median: observed	12.00000	6.00000	3.000000	21.00000
expected	12.29268	5.63415	3.073171	
obs.-exp.	-0,29268	0.36585	-0,073171	
> Median: observed	12.00000	5.00000	3.000000	20.00000
expected	11.70732	5.36585	2.926829	
obs.-exp.	0.29268	-0,36585	0.073171	
Total: observed	24.00000	11.00000	6.000000	41.00000

Depend.: PC aa C36:6	Kruskal-Wallis ANOVA by Ranks; PC aa C36:6<br>Independent (grouping) variable: Hashimoto 1 Hypo-no-Hashimoto 2 control 3<br>Kruskal-Wallis test: H (2, N= 41) =1,206234 p =,5471			
	Code	Valid N	Sum of Ranks	Mean Rank
1	1	24	534.0000	22.25000
2	2	11	229.5000	20.86364
3	3	6	97.5000	16.25000

Dependent: PC aa C36:6	Median Test, Overall Median = ,596000; PC aa C36:6<br>Independent (grouping) variable: Hashimoto 1 Hypo-no-Hashimoto 2 control 3<br>Chi-Square = ,9003878 df = 2 p = ,6375			
	1	2	3	Total
<= Median: observed	11.00000	6.00000	4.000000	21.00000
expected	12.29268	5.63415	3.073171	
obs.-exp.	-1,29268	0.36585	0.926829	
> Median: observed	13.00000	5.00000	2.000000	20.00000
expected	11.70732	5.36585	2.926829	
obs.-exp.	1.29268	-0,36585	-0,926829	
Total: observed	24.00000	11.00000	6.000000	41.00000

Depend.: PC aa C38:0	Kruskal-Wallis ANOVA by Ranks; PC aa C38:0<br>Independent (grouping) variable: Hashimoto 1 Hypo-no-Hashimoto 2 control 3<br>Kruskal-Wallis test: H (2, N= 41) =3,748093 p =,1535			
	Code	Valid N	Sum of Ranks	Mean Rank
1	1	24	570.0000	23.75000
2	2	11	208.0000	18.90909
3	3	6	83.0000	13.83333

Dependent: PC aa C38:0	Median Test, Overall Median = 2,06000; PC aa C38:0<br>Independent (grouping) variable: Hashimoto 1 Hypo-no-Hashimoto 2 control 3<br>Chi-Square = ,9003878 df = 2 p = ,6375			
	1	2	3	Total
<= Median: observed	11.00000	6.00000	4.000000	21.00000
expected	12.29268	5.63415	3.073171	
obs.-exp.	-1,29268	0.36585	0.926829	
> Median: observed	13.00000	5.00000	2.000000	20.00000
expected	11.70732	5.36585	2.926829	
obs.-exp.	1.29268	-0,36585	-0,926829	
Total: observed	24.00000	11.00000	6.000000	41.00000

Depend.: PC aa C38:1	Kruskal-Wallis ANOVA by Ranks; PC aa C38:1<br>Independent (grouping) variable: Hashimoto 1 Hypo-no-Hashimoto 2 control 3<br>Kruskal-Wallis test: H (2, N= 41) =2,671646 p =,2629			
	Code	Valid N	Sum of Ranks	Mean Rank
1	1	24	485.0000	20.20833
2	2	11	280.0000	25.45455
3	3	6	96.0000	16.00000

Dependent: PC aa C38:1	Median Test, Overall Median = ,516000; PC aa C38:1<br>Independent (grouping) variable: Hashimoto 1 Hypo-no-Hashimoto 2 control 3<br>Chi-Square = 3,462518 df = 2 p = ,1771			
	1	2	3	Total
<= Median: observed	12.00000	4.00000	5.00000	21.00000
expected	12.29268	5.63415	3.07317	
obs.-exp.	-0,29268	-1,63415	1.92683	
> Median: observed	12.00000	7.00000	1.00000	20.00000
expected	11.70732	5.36585	2.92683	
obs.-exp.	0.29268	1.63415	-1,92683	
Total: observed	24.00000	11.00000	6.00000	41.00000

Depend.: PC aa C38:3	Kruskal-Wallis ANOVA by Ranks; PC aa C38:3<br>Independent (grouping) variable: Hashimoto 1 Hypo-no-Hashimoto 2 control 3<br>Kruskal-Wallis test: H (2, N= 41) =4,919548 p =,0855			
	Code	Valid N	Sum of Ranks	Mean Rank
1	1	24	585.0000	24.37500
2	2	11	192.0000	17.45455
3	3	6	84.0000	14.00000

Dependent: PC aa C38:3	Median Test, Overall Median = 27,9000; PC aa C38:3<br>Independent (grouping) variable: Hashimoto 1 Hypo-no-Hashimoto 2 control 3<br>Chi-Square = 4,963411 df = 2 p = ,0836			
	1	2	3	Total
<= Median: observed	9.00000	7.00000	5.00000	21.00000
expected	12.29268	5.63415	3.07317	
obs.-exp.	-3,29268	1.36585	1.92683	
> Median: observed	15.00000	4.00000	1.00000	20.00000
expected	11.70732	5.36585	2.92683	
obs.-exp.	3.29268	-1,36585	-1,92683	
Total: observed	24.00000	11.00000	6.00000	41.00000

Depend.: PC aa C38:4	Kruskal-Wallis ANOVA by Ranks; PC aa C38:4<br>Independent (grouping) variable: Hashimoto 1 Hypo-no-Hashimoto 2 control 3<br>Kruskal-Wallis test: H (2, N= 41) =2,932727 p =,2308			
	Code	Valid N	Sum of Ranks	Mean Rank
1	1	24	568.5000	23.68750
2	2	11	186.0000	16.90909
3	3	6	106.5000	17.75000

Dependent: PC aa C38:4	Median Test, Overall Median = 73,2000; PC aa C38:4<br>Independent (grouping) variable: Hashimoto 1 Hypo-no-Hashimoto 2 control 3<br>Chi-Square = ,9610299 df = 2 p = ,6185			
	1	2	3	Total
<= Median: observed	11.00000	7.00000	3.000000	21.00000
expected	12.29268	5.63415	3.073171	
obs.-exp.	-1,29268	1.36585	-0,073171	
> Median: observed	13.00000	4.00000	3.000000	20.00000
expected	11.70732	5.36585	2.926829	
obs.-exp.	1.29268	-1,36585	0.073171	
Total: observed	24.00000	11.00000	6.000000	41.00000

Depend.: PC aa C38:5	Kruskal-Wallis ANOVA by Ranks; PC aa C38:5<br>Independent (grouping) variable: Hashimoto 1 Hypo-no-Hashimoto 2 control 3<br>Kruskal-Wallis test: H (2, N= 41) =,5155108 p =,7728			
	Code	Valid N	Sum of Ranks	Mean Rank
1	1	24	530.0000	22.08333
2	2	11	219.0000	19.90909
3	3	6	112.0000	18.66667

Dependent: PC aa C38:5	Median Test, Overall Median = 34,8000; PC aa C38:5<br>Independent (grouping) variable: Hashimoto 1 Hypo-no-Hashimoto 2 control 3<br>Chi-Square = ,5409598 df = 2 p = ,7630			
	1	2	3	Total
<= Median: observed	12.00000	6.00000	4.000000	22.00000
expected	12.87805	5.90244	3.219512	
obs.-exp.	-0,87805	0.09756	0.780488	
> Median: observed	12.00000	5.00000	2.000000	19.00000
expected	11.12195	5.09756	2.780488	
obs.-exp.	0.87805	-0,09756	-0,780488	
Total: observed	24.00000	11.00000	6.000000	41.00000

Depend.: PC aa C38:6	Kruskal-Wallis ANOVA by Ranks; PC aa C38:6<br>Independent (grouping) variable: Hashimoto 1 Hypo-no-Hashimoto 2 control 3<br>Kruskal-Wallis test: H (2, N= 41) =2,574167 p =,2761			
	Code	Valid N	Sum of Ranks	Mean Rank
1	1	24	514.5000	21.43750
2	2	11	261.5000	23.77273
3	3	6	85.0000	14.16667

Median Test, Overall Median = 58,2000; PC aa C38:6
Independent (grouping) variable: Hashimoto 1 Hypo-no-Hashimoto 2 control 3
Chi-Square = ,7336219 df = 2 p = ,6929

Dependent: PC aa C38:6	1	2	3	Total
<= Median: observed	12.00000	5.00000	4.000000	21.00000
expected	12.29268	5.63415	3.073171	
obs.-exp.	-0,29268	-0,63415	0.926829	
> Median: observed	12.00000	6.00000	2.000000	20.00000
expected	11.70732	5.36585	2.926829	
obs.-exp.	0.29268	0.63415	-0,926829	
Total: observed	24.00000	11.00000	6.000000	41.00000

Kruskal-Wallis ANOVA by Ranks; PC aa C40:1
Independent (grouping) variable: Hashimoto 1 Hypo-no-Hashimoto 2 control 3
Kruskal-Wallis test: H (2, N= 41) =31,40706 p =,0000

Depend.: PC aa C40:1	Code	Valid N	Sum of Ranks	Mean Rank
1	1	24	582.0000	24.25000
2	2	11	258.0000	23.45455
3	3	6	21.0000	3.50000

Median Test, Overall Median = 101,000; PC aa C40:1
Independent (grouping) variable: Hashimoto 1 Hypo-no-Hashimoto 2 control 3
Chi-Square = 0,000000 df = 2 p = 1,000

Dependent: PC aa C40:1	1	2	3	Total
<= Median: observed	24.00000	11.00000	6.000000	41.00000
expected	24.00000	11.00000	6.000000	
obs.-exp.	0.00000	0.00000	0.000000	
> Median: observed	0.00000	0.00000	0.000000	0.00000
expected	0.00000	0.00000	0.000000	
obs.-exp.	0.00000	0.00000	0.000000	
Total: observed	24.00000	11.00000	6.000000	41.00000

Kruskal-Wallis ANOVA by Ranks; PC aa C40:2
Independent (grouping) variable: Hashimoto 1 Hypo-no-Hashimoto 2 control 3
Kruskal-Wallis test: H (2, N= 41) =,0892477 p =,9564

Depend.: PC aa C40:2	Code	Valid N	Sum of Ranks	Mean Rank
1	1	24	511.0000	21.29167
2	2	11	232.0000	21.09091
3	3	6	118.0000	19.66667

Dependent: PC aa C40:2	Median Test, Overall Median = ,173000; PC aa C40:2<br>Independent (grouping) variable: Hashimoto 1 Hypo-no-Hashimoto 2 control 3<br>Chi-Square = ,0665584 df = 2 p = ,9673			
	1	2	3	Total
<= Median: observed	12.00000	6.00000	3.000000	21.00000
expected	12.29268	5.63415	3.073171	
obs.-exp.	-0,29268	0.36585	-0,073171	
> Median: observed	12.00000	5.00000	3.000000	20.00000
expected	11.70732	5.36585	2.926829	
obs.-exp.	0.29268	-0,36585	0.073171	
Total: observed	24.00000	11.00000	6.000000	41.00000

Depend.: PC aa C40:3	Kruskal-Wallis ANOVA by Ranks; PC aa C40:3<br>Independent (grouping) variable: Hashimoto 1 Hypo-no-Hashimoto 2 control 3<br>Kruskal-Wallis test: H (2, N= 41) =,0612556 p =,9698			
	Code	Valid N	Sum of Ranks	Mean Rank
1	1	24	502.0000	20.91667
2	2	11	238.0000	21.63636
3	3	6	121.0000	20.16667

Dependent: PC aa C40:3	Median Test, Overall Median = ,306000; PC aa C40:3<br>Independent (grouping) variable: Hashimoto 1 Hypo-no-Hashimoto 2 control 3<br>Chi-Square = ,2333243 df = 2 p = ,8899			
	1	2	3	Total
<= Median: observed	13.00000	5.00000	3.000000	21.00000
expected	12.29268	5.63415	3.073171	
obs.-exp.	0.70732	-0,63415	-0,073171	
> Median: observed	11.00000	6.00000	3.000000	20.00000
expected	11.70732	5.36585	2.926829	
obs.-exp.	-0,70732	0.63415	0.073171	
Total: observed	24.00000	11.00000	6.000000	41.00000

Depend.: PC aa C40:4	Kruskal-Wallis ANOVA by Ranks; PC aa C40:4<br>Independent (grouping) variable: Hashimoto 1 Hypo-no-Hashimoto 2 control 3<br>Kruskal-Wallis test: H (2, N= 41) =3,180061 p =,2039			
	Code	Valid N	Sum of Ranks	Mean Rank
1	1	24	571.0000	23.79167
2	2	11	192.0000	17.45455
3	3	6	98.0000	16.33333

Dependent: PC aa C40:4	Median Test, Overall Median = 1,78000; PC aa C40:4<br>Independent (grouping) variable: Hashimoto 1 Hypo-no-Hashimoto 2 control 3<br>Chi-Square = 2,180244 df = 2 p = ,3362			
	1	2	3	Total
<= Median: observed	12.00000	6.00000	5.00000	23.00000
expected	13.46341	6.17073	3.36585	
obs.-exp.	-1,46341	-0,17073	1.63415	
> Median: observed	12.00000	5.00000	1.00000	18.00000
expected	10.53659	4.82927	2.63415	
obs.-exp.	1.46341	0.17073	-1,63415	
Total: observed	24.00000	11.00000	6.00000	41.00000

Depend.: PC aa C40:5	Kruskal-Wallis ANOVA by Ranks; PC aa C40:5<br>Independent (grouping) variable: Hashimoto 1 Hypo-no-Hashimoto 2 control 3<br>Kruskal-Wallis test: H (2, N= 41) =,3172856 p =,8533			
	Code	Valid N	Sum of Ranks	Mean Rank
1	1	24	521.0000	21.70833
2	2	11	212.0000	19.27273
3	3	6	128.0000	21.33333

Dependent: PC aa C40:5	Median Test, Overall Median = 4,88000; PC aa C40:5<br>Independent (grouping) variable: Hashimoto 1 Hypo-no-Hashimoto 2 control 3<br>Chi-Square = 1,461328 df = 2 p = ,4816			
	1	2	3	Total
<= Median: observed	12.00000	7.00000	2.00000	21.00000
expected	12.29268	5.63415	3.07317	
obs.-exp.	-0,29268	1.36585	-1,07317	
> Median: observed	12.00000	4.00000	4.00000	20.00000
expected	11.70732	5.36585	2.92683	
obs.-exp.	0.29268	-1,36585	1.07317	
Total: observed	24.00000	11.00000	6.00000	41.00000

Depend.: PC aa C40:6	Kruskal-Wallis ANOVA by Ranks; PC aa C40:6<br>Independent (grouping) variable: Hashimoto 1 Hypo-no-Hashimoto 2 control 3<br>Kruskal-Wallis test: H (2, N= 41) =2,594611 p =,2733			
	Code	Valid N	Sum of Ranks	Mean Rank
1	1	24	521.0000	21.70833
2	2	11	256.5000	23.31818
3	3	6	83.5000	13.91667

Dependent: PC aa C40:6	Median Test, Overall Median = 15,6000; PC aa C40:6<br>Independent (grouping) variable: Hashimoto 1 Hypo-no-Hashimoto 2 control 3<br>Chi-Square = 2,901578 df = 2 p = ,2344			
	1	2	3	Total
<= Median: observed	11.00000	5.00000	5.00000	21.00000
expected	12.29268	5.63415	3.07317	
obs.-exp.	-1,29268	-0,63415	1.92683	
> Median: observed	13.00000	6.00000	1.00000	20.00000
expected	11.70732	5.36585	2.92683	
obs.-exp.	1.29268	0.63415	-1,92683	
Total: observed	24.00000	11.00000	6.00000	41.00000

Depend.: PC aa C42:0	Kruskal-Wallis ANOVA by Ranks; PC aa C42:0<br>Independent (grouping) variable: Hashimoto 1 Hypo-no-Hashimoto 2 control 3<br>Kruskal-Wallis test: H (2, N= 41) =,2267513 p =,8928			
	Code	Valid N	Sum of Ranks	Mean Rank
1	1	24	489.0000	20.37500
2	2	11	234.5000	21.31818
3	3	6	137.5000	22.91667

Dependent: PC aa C42:0	Median Test, Overall Median = ,328000; PC aa C42:0<br>Independent (grouping) variable: Hashimoto 1 Hypo-no-Hashimoto 2 control 3<br>Chi-Square = ,9003878 df = 2 p = ,6375			
	1	2	3	Total
<= Median: observed	13.00000	6.00000	2.00000	21.00000
expected	12.29268	5.63415	3.07317	
obs.-exp.	0.70732	0.36585	-1,07317	
> Median: observed	11.00000	5.00000	4.00000	20.00000
expected	11.70732	5.36585	2.92683	
obs.-exp.	-0,70732	-0,36585	1.07317	
Total: observed	24.00000	11.00000	6.00000	41.00000

Depend.: PC aa C42:1	Kruskal-Wallis ANOVA by Ranks; PC aa C42:1<br>Independent (grouping) variable: Hashimoto 1 Hypo-no-Hashimoto 2 control 3<br>Kruskal-Wallis test: H (2, N= 41) =,2312210 p =,8908			
	Code	Valid N	Sum of Ranks	Mean Rank
1	1	24	487.0000	20.29167
2	2	11	246.0000	22.36364
3	3	6	128.0000	21.33333

Dependent: PC aa C42:1	Median Test, Overall Median = ,172000; PC aa C42:1<br>Independent (grouping) variable: Hashimoto 1 Hypo-no-Hashimoto 2 control 3<br>Chi-Square = ,0665584 df = 2 p = ,9673			
	1	2	3	Total
<= Median: observed	12.00000	6.00000	3.000000	21.00000
expected	12.29268	5.63415	3.073171	
obs.-exp.	-0,29268	0.36585	-0,073171	
> Median: observed	12.00000	5.00000	3.000000	20.00000
expected	11.70732	5.36585	2.926829	
obs.-exp.	0.29268	-0,36585	0.073171	
Total: observed	24.00000	11.00000	6.000000	41.00000

Depend.: PC aa C42:2	Kruskal-Wallis ANOVA by Ranks; PC aa C42:2<br>Independent (grouping) variable: Hashimoto 1 Hypo-no-Hashimoto 2 control 3<br>Kruskal-Wallis test: H (2, N= 41) =,0107442 p =,9946			
	Code	Valid N	Sum of Ranks	Mean Rank
1	1	24	501.5000	20.89583
2	2	11	234.5000	21.31818
3	3	6	125.0000	20.83333

Dependent: PC aa C42:2	Median Test, Overall Median = ,133000; PC aa C42:2<br>Independent (grouping) variable: Hashimoto 1 Hypo-no-Hashimoto 2 control 3<br>Chi-Square = ,0665584 df = 2 p = ,9673			
	1	2	3	Total
<= Median: observed	12.00000	6.00000	3.000000	21.00000
expected	12.29268	5.63415	3.073171	
obs.-exp.	-0,29268	0.36585	-0,073171	
> Median: observed	12.00000	5.00000	3.000000	20.00000
expected	11.70732	5.36585	2.926829	
obs.-exp.	0.29268	-0,36585	0.073171	
Total: observed	24.00000	11.00000	6.000000	41.00000

Depend.: PC aa C42:4	Kruskal-Wallis ANOVA by Ranks; PC aa C42:4<br>Independent (grouping) variable: Hashimoto 1 Hypo-no-Hashimoto 2 control 3<br>Kruskal-Wallis test: H (2, N= 41) =,8079135 p =,6677			
	Code	Valid N	Sum of Ranks	Mean Rank
1	1	24	517.0000	21.54167
2	2	11	203.0000	18.45455
3	3	6	141.0000	23.50000

Dependent: PC aa C42:4	Median Test, Overall Median = ,104000; PC aa C42:4<br>Independent (grouping) variable: Hashimoto 1 Hypo-no-Hashimoto 2 control 3<br>Chi-Square = 1,461328 df = 2 p = ,4816			
	1	2	3	Total
<= Median: observed	12.00000	7.00000	2.00000	21.00000
expected	12.29268	5.63415	3.07317	
obs.-exp.	-0,29268	1.36585	-1,07317	
> Median: observed	12.00000	4.00000	4.00000	20.00000
expected	11.70732	5.36585	2.92683	
obs.-exp.	0.29268	-1,36585	1.07317	
Total: observed	24.00000	11.00000	6.00000	41.00000

Depend.: PC aa C42:5	Kruskal-Wallis ANOVA by Ranks; PC aa C42:5<br>Independent (grouping) variable: Hashimoto 1 Hypo-no-Hashimoto 2 control 3<br>Kruskal-Wallis test: H (2, N= 41) =,1114412 p =,9458			
	Code	Valid N	Sum of Ranks	Mean Rank
1	1	24	503.0000	20.95833
2	2	11	239.5000	21.77273
3	3	6	118.5000	19.75000

Dependent: PC aa C42:5	Median Test, Overall Median = ,199000; PC aa C42:5<br>Independent (grouping) variable: Hashimoto 1 Hypo-no-Hashimoto 2 control 3<br>Chi-Square = ,7336219 df = 2 p = ,6929			
	1	2	3	Total
<= Median: observed	12.00000	5.00000	4.000000	21.00000
expected	12.29268	5.63415	3.073171	
obs.-exp.	-0,29268	-0,63415	0.926829	
> Median: observed	12.00000	6.00000	2.000000	20.00000
expected	11.70732	5.36585	2.926829	
obs.-exp.	0.29268	0.63415	-0,926829	
Total: observed	24.00000	11.00000	6.000000	41.00000

Depend.: PC aa C42:6	Kruskal-Wallis ANOVA by Ranks; PC aa C42:6<br>Independent (grouping) variable: Hashimoto 1 Hypo-no-Hashimoto 2 control 3<br>Kruskal-Wallis test: H (2, N= 41) =,0231049 p =,9885			
	Code	Valid N	Sum of Ranks	Mean Rank
1	1	24	509.0000	21.20833
2	2	11	226.0000	20.54545
3	3	6	126.0000	21.00000

Dependent: PC aa C42:6	Median Test, Overall Median = ,226000; PC aa C42:6<br>Independent (grouping) variable: Hashimoto 1 Hypo-no-Hashimoto 2 control 3<br>Chi-Square = ,6018921 df = 2 p = ,7401			
	1	2	3	Total
<= Median: observed	12.00000	7.00000	3.000000	22.00000
expected	12.87805	5.90244	3.219512	
obs.-exp.	-0,87805	1.09756	-0,219512	
> Median: observed	12.00000	4.00000	3.000000	19.00000
expected	11.12195	5.09756	2.780488	
obs.-exp.	0.87805	-1,09756	0.219512	
Total: observed	24.00000	11.00000	6.000000	41.00000

Depend.: PC ae C30:0	Kruskal-Wallis ANOVA by Ranks; PC ae C30:0<br>Independent (grouping) variable: Hashimoto 1 Hypo-no-Hashimoto 2 control 3<br>Kruskal-Wallis test: H (2, N= 41) =5,768472 p =,0559			
	Code	Valid N	Sum of Ranks	Mean Rank
1	1	24	580.5000	24.18750
2	2	11	212.0000	19.27273
3	3	6	68.5000	11.41667

Dependent: PC ae C30:0	Median Test, Overall Median = ,325000; PC ae C30:0<br>Independent (grouping) variable: Hashimoto 1 Hypo-no-Hashimoto 2 control 3<br>Chi-Square = 7,571023 df = 2 p = ,0227			
	1	2	3	Total
<= Median: observed	9.00000	6.00000	6.00000	21.00000
expected	12.29268	5.63415	3.07317	
obs.-exp.	-3,29268	0.36585	2.92683	
> Median: observed	15.00000	5.00000	0.00000	20.00000
expected	11.70732	5.36585	2.92683	
obs.-exp.	3.29268	-0,36585	-2,92683	
Total: observed	24.00000	11.00000	6.00000	41.00000

Depend.: PC ae C30:1	Kruskal-Wallis ANOVA by Ranks; PC ae C30:1<br>Independent (grouping) variable: Hashimoto 1 Hypo-no-Hashimoto 2 control 3<br>Kruskal-Wallis test: H (2, N= 41) =8,596205 p =,0136			
	Code	Valid N	Sum of Ranks	Mean Rank
1	1	24	583.5000	24.31250
2	2	11	218.0000	19.81818
3	3	6	59.5000	9.91667

Dependent: PC ae C30:1	Median Test, Overall Median = 101,000; PC ae C30:1<br>Independent (grouping) variable: Hashimoto 1 Hypo-no-Hashimoto 2 control 3<br>Chi-Square = 0,000000 df = 2 p = 1,000			
	1	2	3	Total
<= Median: observed	24.00000	11.00000	6.000000	41.00000
expected	24.00000	11.00000	6.000000	
obs.-exp.	0.00000	0.00000	0.000000	
> Median: observed	0.00000	0.00000	0.000000	0.00000
expected	0.00000	0.00000	0.000000	
obs.-exp.	0.00000	0.00000	0.000000	
Total: observed	24.00000	11.00000	6.000000	41.00000

Depend.: PC ae C30:2	Kruskal-Wallis ANOVA by Ranks; PC ae C30:2<br>Independent (grouping) variable: Hashimoto 1 Hypo-no-Hashimoto 2 control 3<br>Kruskal-Wallis test: H (2, N= 41) =,2798519 p =,8694			
	Code	Valid N	Sum of Ranks	Mean Rank
1	1	24	501.5000	20.89583
2	2	11	245.0000	22.27273
3	3	6	114.5000	19.08333

Dependent: PC ae C30:2	Median Test, Overall Median = ,083000; PC ae C30:2<br>Independent (grouping) variable: Hashimoto 1 Hypo-no-Hashimoto 2 control 3<br>Chi-Square = ,2333243 df = 2 p = ,8899			
	1	2	3	Total
<= Median: observed	13.00000	5.00000	3.000000	21.00000
expected	12.29268	5.63415	3.073171	
obs.-exp.	0.70732	-0,63415	-0,073171	
> Median: observed	11.00000	6.00000	3.000000	20.00000
expected	11.70732	5.36585	2.926829	
obs.-exp.	-0,70732	0.63415	0.073171	
Total: observed	24.00000	11.00000	6.000000	41.00000

Depend.: PC ae C32:1	Kruskal-Wallis ANOVA by Ranks; PC ae C32:1<br>Independent (grouping) variable: Hashimoto 1 Hypo-no-Hashimoto 2 control 3<br>Kruskal-Wallis test: H (2, N= 41) =4,827489 p =,0895			
	Code	Valid N	Sum of Ranks	Mean Rank
1	1	24	583.5000	24.31250
2	2	11	194.5000	17.68182
3	3	6	83.0000	13.83333

Dependent:<br>PC ae C32:1	Median Test, Overall Median = 2,25000; PC ae C32:1<br>Independent (grouping) variable: Hashimoto 1 Hypo-no-Hashimoto 2 control 3<br>Chi-Square = ,9003878 df = 2 p = ,6375			
	1	2	3	Total
<= Median: observed	11.00000	6.00000	4.000000	21.00000
expected	12.29268	5.63415	3.073171	
obs.-exp.	-1,29268	0.36585	0.926829	
> Median: observed	13.00000	5.00000	2.000000	20.00000
expected	11.70732	5.36585	2.926829	
obs.-exp.	1.29268	-0,36585	-0,926829	
Total: observed	24.00000	11.00000	6.000000	41.00000

Depend.:<br>PC ae C32:2	Kruskal-Wallis ANOVA by Ranks; PC ae C32:2<br>Independent (grouping) variable: Hashimoto 1 Hypo-no-Hashimoto 2 control 3<br>Kruskal-Wallis test: H (2, N= 41) =3,919400 p =,1409			
	Code	Valid N	Sum of Ranks	Mean Rank
1	1	24	574.5000	23.93750
2	2	11	201.0000	18.27273
3	3	6	85.5000	14.25000

Dependent:<br>PC ae C32:2	Median Test, Overall Median = ,561000; PC ae C32:2<br>Independent (grouping) variable: Hashimoto 1 Hypo-no-Hashimoto 2 control 3<br>Chi-Square = 4,963411 df = 2 p = ,0836			
	1	2	3	Total
<= Median: observed	9.00000	7.00000	5.00000	21.00000
expected	12.29268	5.63415	3.07317	
obs.-exp.	-3,29268	1.36585	1.92683	
> Median: observed	15.00000	4.00000	1.00000	20.00000
expected	11.70732	5.36585	2.92683	
obs.-exp.	3.29268	-1,36585	-1,92683	
Total: observed	24.00000	11.00000	6.00000	41.00000

Depend.:<br>PC ae C34:0	Kruskal-Wallis ANOVA by Ranks; PC ae C34:0<br>Independent (grouping) variable: Hashimoto 1 Hypo-no-Hashimoto 2 control 3<br>Kruskal-Wallis test: H (2, N= 41) =7,436659 p =,0243			
	Code	Valid N	Sum of Ranks	Mean Rank
1	1	24	596.5000	24.85417
2	2	11	199.5000	18.13636
3	3	6	65.0000	10.83333

Dependent: PC ae C34:0	Median Test, Overall Median = ,970000; PC ae C34:0<br>Independent (grouping) variable: Hashimoto 1 Hypo-no-Hashimoto 2 control 3<br>Chi-Square = 6,573256 df = 2 p = ,0374			
	1	2	3	Total
<= Median: observed	10.00000	6.00000	6.00000	22.00000
expected	12.87805	5.90244	3.21951	
obs.-exp.	-2,87805	0.09756	2.78049	
> Median: observed	14.00000	5.00000	0.00000	19.00000
expected	11.12195	5.09756	2.78049	
obs.-exp.	2.87805	-0,09756	-2,78049	
Total: observed	24.00000	11.00000	6.00000	41.00000

Depend.: PC ae C34:1	Kruskal-Wallis ANOVA by Ranks; PC ae C34:1<br>Independent (grouping) variable: Hashimoto 1 Hypo-no-Hashimoto 2 control 3<br>Kruskal-Wallis test: H (2, N= 41) =2,455415 p =,2930			
	Code	Valid N	Sum of Ranks	Mean Rank
1	1	24	560.5000	23.35417
2	2	11	205.5000	18.68182
3	3	6	95.0000	15.83333

Dependent: PC ae C34:1	Median Test, Overall Median = 8,38000; PC ae C34:1<br>Independent (grouping) variable: Hashimoto 1 Hypo-no-Hashimoto 2 control 3<br>Chi-Square = 2,128391 df = 2 p = ,3450			
	1	2	3	Total
<= Median: observed	10.00000	7.00000	4.000000	21.00000
expected	12.29268	5.63415	3.073171	
obs.-exp.	-2,29268	1.36585	0.926829	
> Median: observed	14.00000	4.00000	2.000000	20.00000
expected	11.70732	5.36585	2.926829	
obs.-exp.	2.29268	-1,36585	-0,926829	
Total: observed	24.00000	11.00000	6.000000	41.00000

Depend.: PC ae C34:2	Kruskal-Wallis ANOVA by Ranks; PC ae C34:2<br>Independent (grouping) variable: Hashimoto 1 Hypo-no-Hashimoto 2 control 3<br>Kruskal-Wallis test: H (2, N= 41) =4,919781 p =,0854			
	Code	Valid N	Sum of Ranks	Mean Rank
1	1	24	587.5000	24.47917
2	2	11	181.5000	16.50000
3	3	6	92.0000	15.33333

Dependent: PC ae C34:2	Median Test, Overall Median = 10,3000; PC ae C34:2<br>Independent (grouping) variable: Hashimoto 1 Hypo-no-Hashimoto 2 control 3<br>Chi-Square = 4,963411 df = 2 p = ,0836			
	1	2	3	Total
<= Median: observed	9.00000	7.00000	5.00000	21.00000
expected	12.29268	5.63415	3.07317	
obs.-exp.	-3,29268	1.36585	1.92683	
> Median: observed	15.00000	4.00000	1.00000	20.00000
expected	11.70732	5.36585	2.92683	
obs.-exp.	3.29268	-1,36585	-1,92683	
Total: observed	24.00000	11.00000	6.00000	41.00000

Depend.: PC ae C34:3	Kruskal-Wallis ANOVA by Ranks; PC ae C34:3<br>Independent (grouping) variable: Hashimoto 1 Hypo-no-Hashimoto 2 control 3<br>Kruskal-Wallis test: H (2, N= 41) =3,485126 p =,1751			
	Code	Valid N	Sum of Ranks	Mean Rank
1	1	24	573.0000	23.87500
2	2	11	195.5000	17.77273
3	3	6	92.5000	15.41667

Dependent: PC ae C34:3	Median Test, Overall Median = 6,50000; PC ae C34:3<br>Independent (grouping) variable: Hashimoto 1 Hypo-no-Hashimoto 2 control 3<br>Chi-Square = 7,586183 df = 2 p = ,0225			
	1	2	3	Total
<= Median: observed	8.00000	8.00000	5.00000	21.00000
expected	12.29268	5.63415	3.07317	
obs.-exp.	-4,29268	2.36585	1.92683	
> Median: observed	16.00000	3.00000	1.00000	20.00000
expected	11.70732	5.36585	2.92683	
obs.-exp.	4.29268	-2,36585	-1,92683	
Total: observed	24.00000	11.00000	6.00000	41.00000

Depend.: PC ae C36:0	Kruskal-Wallis ANOVA by Ranks; PC ae C36:0<br>Independent (grouping) variable: Hashimoto 1 Hypo-no-Hashimoto 2 control 3<br>Kruskal-Wallis test: H (2, N= 41) =,8114601 p =,6665			
	Code	Valid N	Sum of Ranks	Mean Rank
1	1	24	538.0000	22.41667
2	2	11	208.0000	18.90909
3	3	6	115.0000	19.16667

Dependent: PC ae C36:0	Median Test, Overall Median = ,546000; PC ae C36:0<br>Independent (grouping) variable: Hashimoto 1 Hypo-no-Hashimoto 2 control 3<br>Chi-Square = ,9003878 df = 2 p = ,6375			
	1	2	3	Total
<= Median: observed	11.00000	6.00000	4.000000	21.00000
expected	12.29268	5.63415	3.073171	
obs.-exp.	-1,29268	0.36585	0.926829	
> Median: observed	13.00000	5.00000	2.000000	20.00000
expected	11.70732	5.36585	2.926829	
obs.-exp.	1.29268	-0,36585	-0,926829	
Total: observed	24.00000	11.00000	6.000000	41.00000

Depend.: PC ae C36:1	Kruskal-Wallis ANOVA by Ranks; PC ae C36:1<br>Independent (grouping) variable: Hashimoto 1 Hypo-no-Hashimoto 2 control 3<br>Kruskal-Wallis test: H (2, N= 41) =2,879686 p =,2370			
	Code	Valid N	Sum of Ranks	Mean Rank
1	1	24	559.0000	23.29167
2	2	11	216.0000	19.63636
3	3	6	86.0000	14.33333

Dependent: PC ae C36:1	Median Test, Overall Median = 5,75000; PC ae C36:1<br>Independent (grouping) variable: Hashimoto 1 Hypo-no-Hashimoto 2 control 3<br>Chi-Square = 2,128391 df = 2 p = ,3450			
	1	2	3	Total
<= Median: observed	10.00000	7.00000	4.000000	21.00000
expected	12.29268	5.63415	3.073171	
obs.-exp.	-2,29268	1.36585	0.926829	
> Median: observed	14.00000	4.00000	2.000000	20.00000
expected	11.70732	5.36585	2.926829	
obs.-exp.	2.29268	-1,36585	-0,926829	
Total: observed	24.00000	11.00000	6.000000	41.00000

Depend.: PC ae C36:2	Kruskal-Wallis ANOVA by Ranks; PC ae C36:2<br>Independent (grouping) variable: Hashimoto 1 Hypo-no-Hashimoto 2 control 3<br>Kruskal-Wallis test: H (2, N= 41) =3,142384 p =,2078			
	Code	Valid N	Sum of Ranks	Mean Rank
1	1	24	568.5000	23.68750
2	2	11	200.5000	18.22727
3	3	6	92.0000	15.33333

Dependent: PC ae C36:2	Median Test, Overall Median = 10,6000; PC ae C36:2<br>Independent (grouping) variable: Hashimoto 1 Hypo-no-Hashimoto 2 control 3<br>Chi-Square = ,9003878 df = 2 p = ,6375			
	1	2	3	Total
<= Median: observed	11.00000	6.00000	4.000000	21.00000
expected	12.29268	5.63415	3.073171	
obs.-exp.	-1,29268	0.36585	0.926829	
> Median: observed	13.00000	5.00000	2.000000	20.00000
expected	11.70732	5.36585	2.926829	
obs.-exp.	1.29268	-0,36585	-0,926829	
Total: observed	24.00000	11.00000	6.000000	41.00000

Depend.: PC ae C36:3	Kruskal-Wallis ANOVA by Ranks; PC ae C36:3<br>Independent (grouping) variable: Hashimoto 1 Hypo-no-Hashimoto 2 control 3<br>Kruskal-Wallis test: H (2, N= 41) =4,301399 p =,1164			
	Code	Valid N	Sum of Ranks	Mean Rank
1	1	24	578.0000	24.08333
2	2	11	167.0000	15.18182
3	3	6	116.0000	19.33333

Dependent: PC ae C36:3	Median Test, Overall Median = 5,77000; PC ae C36:3<br>Independent (grouping) variable: Hashimoto 1 Hypo-no-Hashimoto 2 control 3<br>Chi-Square = 2,128391 df = 2 p = ,3450			
	1	2	3	Total
<= Median: observed	10.00000	7.00000	4.000000	21.00000
expected	12.29268	5.63415	3.073171	
obs.-exp.	-2,29268	1.36585	0.926829	
> Median: observed	14.00000	4.00000	2.000000	20.00000
expected	11.70732	5.36585	2.926829	
obs.-exp.	2.29268	-1,36585	-0,926829	
Total: observed	24.00000	11.00000	6.000000	41.00000

Depend.: PC ae C36:4	Kruskal-Wallis ANOVA by Ranks; PC ae C36:4<br>Independent (grouping) variable: Hashimoto 1 Hypo-no-Hashimoto 2 control 3<br>Kruskal-Wallis test: H (2, N= 41) =8,468897 p =,0145			
	Code	Valid N	Sum of Ranks	Mean Rank
1	1	24	613.0000	25.54167
2	2	11	151.5000	13.77273
3	3	6	96.5000	16.08333

Dependent: PC ae C36:4	Median Test, Overall Median = 14,5000; PC ae C36:4 Independent (grouping) variable: Hashimoto 1 Hypo-no-Hashimoto 2 control 3 Chi-Square = 5,933685 df = 2 p = ,0515			
	1	2	3	Total
<= Median: observed	9.00000	9.00000	3.000000	21.00000
expected	12.29268	5.63415	3.073171	
obs.-exp.	-3,29268	3.36585	-0,073171	
> Median: observed	15.00000	2.00000	3.000000	20.00000
expected	11.70732	5.36585	2.926829	
obs.-exp.	3.29268	-3,36585	0.073171	
Total: observed	24.00000	11.00000	6.000000	41.00000

Depend.: PC ae C36:5	Kruskal-Wallis ANOVA by Ranks; PC ae C36:5 Independent (grouping) variable: Hashimoto 1 Hypo-no-Hashimoto 2 control 3 Kruskal-Wallis test: H (2, N= 41) =7,218848 p =,0271			
	Code	Valid N	Sum of Ranks	Mean Rank
1	1	24	603.0000	25.12500
2	2	11	181.0000	16.45455
3	3	6	77.0000	12.83333

Dependent: PC ae C36:5	Median Test, Overall Median = 8,89000; PC ae C36:5 Independent (grouping) variable: Hashimoto 1 Hypo-no-Hashimoto 2 control 3 Chi-Square = 2,128391 df = 2 p = ,3450			
	1	2	3	Total
<= Median: observed	10.00000	7.00000	4.000000	21.00000
expected	12.29268	5.63415	3.073171	
obs.-exp.	-2,29268	1.36585	0.926829	
> Median: observed	14.00000	4.00000	2.000000	20.00000
expected	11.70732	5.36585	2.926829	
obs.-exp.	2.29268	-1,36585	-0,926829	
Total: observed	24.00000	11.00000	6.000000	41.00000

Depend.: PC ae C38:0	Kruskal-Wallis ANOVA by Ranks; PC ae C38:0 Independent (grouping) variable: Hashimoto 1 Hypo-no-Hashimoto 2 control 3 Kruskal-Wallis test: H (2, N= 41) =,4227323 p =,8095			
	Code	Valid N	Sum of Ranks	Mean Rank
1	1	24	506.0000	21.08333
2	2	11	245.0000	22.27273
3	3	6	110.0000	18.33333

Dependent: PC ae C38:0	Median Test, Overall Median = 1,38000; PC ae C38:0<br>Independent (grouping) variable: Hashimoto 1 Hypo-no-Hashimoto 2 control 3<br>Chi-Square = ,2333243 df = 2 p = ,8899			
	1	2	3	Total
<= Median: observed	13.00000	5.00000	3.000000	21.00000
expected	12.29268	5.63415	3.073171	
obs.-exp.	0.70732	-0,63415	-0,073171	
> Median: observed	11.00000	6.00000	3.000000	20.00000
expected	11.70732	5.36585	2.926829	
obs.-exp.	-0,70732	0.63415	0.073171	
Total: observed	24.00000	11.00000	6.000000	41.00000

Depend.: PC ae C38:1	Kruskal-Wallis ANOVA by Ranks; PC ae C38:1<br>Independent (grouping) variable: Hashimoto 1 Hypo-no-Hashimoto 2 control 3<br>Kruskal-Wallis test: H (2, N= 41) =4,803251 p =,0906			
	Code	Valid N	Sum of Ranks	Mean Rank
1	1	24	503.0000	20.95833
2	2	11	283.0000	25.72727
3	3	6	75.0000	12.50000

Dependent: PC ae C38:1	Median Test, Overall Median = ,208000; PC ae C38:1<br>Independent (grouping) variable: Hashimoto 1 Hypo-no-Hashimoto 2 control 3<br>Chi-Square = 5,084695 df = 2 p = ,0787			
	1	2	3	Total
<= Median: observed	13.00000	3.00000	5.00000	21.00000
expected	12.29268	5.63415	3.07317	
obs.-exp.	0.70732	-2,63415	1.92683	
> Median: observed	11.00000	8.00000	1.00000	20.00000
expected	11.70732	5.36585	2.92683	
obs.-exp.	-0,70732	2.63415	-1,92683	
Total: observed	24.00000	11.00000	6.00000	41.00000

Depend.: PC ae C38:2	Kruskal-Wallis ANOVA by Ranks; PC ae C38:2<br>Independent (grouping) variable: Hashimoto 1 Hypo-no-Hashimoto 2 control 3<br>Kruskal-Wallis test: H (2, N= 41) =,8617820 p =,6499			
	Code	Valid N	Sum of Ranks	Mean Rank
1	1	24	528.0000	22.00000
2	2	11	199.5000	18.13636
3	3	6	133.5000	22.25000

Dependent: PC ae C38:2	Median Test, Overall Median = 1,00000; PC ae C38:2<br>Independent (grouping) variable: Hashimoto 1 Hypo-no-Hashimoto 2 control 3<br>Chi-Square = ,1500512 df = 2 p = ,9277			
PC ae C38:2	1	2	3	Total
<= Median: observed	14.00000	6.00000	3.000000	23.00000
expected	13.46341	6.17073	3.365854	
obs.-exp.	0.53659	-0,17073	-0,365854	
> Median: observed	10.00000	5.00000	3.000000	18.00000
expected	10.53659	4.82927	2.634146	
obs.-exp.	-0,53659	0.17073	0.365854	
Total: observed	24.00000	11.00000	6.000000	41.00000

Depend.: PC ae C38:3	Kruskal-Wallis ANOVA by Ranks; PC ae C38:3<br>Independent (grouping) variable: Hashimoto 1 Hypo-no-Hashimoto 2 control 3<br>Kruskal-Wallis test: H (2, N= 41) =4,608795 p =,0998			
	Code	Valid N	Sum of Ranks	Mean Rank
1	1	24	569.5000	23.72917
2	2	11	218.5000	19.86364
3	3	6	73.0000	12.16667

Dependent: PC ae C38:3	Median Test, Overall Median = 2,30000; PC ae C38:3<br>Independent (grouping) variable: Hashimoto 1 Hypo-no-Hashimoto 2 control 3<br>Chi-Square = 3,401876 df = 2 p = ,1825			
PC ae C38:3	1	2	3	Total
<= Median: observed	10.00000	6.00000	5.00000	21.00000
expected	12.29268	5.63415	3.07317	
obs.-exp.	-2,29268	0.36585	1.92683	
> Median: observed	14.00000	5.00000	1.00000	20.00000
expected	11.70732	5.36585	2.92683	
obs.-exp.	2.29268	-0,36585	-1,92683	
Total: observed	24.00000	11.00000	6.00000	41.00000

Depend.: PC ae C38:4	Kruskal-Wallis ANOVA by Ranks; PC ae C38:4<br>Independent (grouping) variable: Hashimoto 1 Hypo-no-Hashimoto 2 control 3<br>Kruskal-Wallis test: H (2, N= 41) =6,069585 p =,0481			
	Code	Valid N	Sum of Ranks	Mean Rank
1	1	24	592.0000	24.66667
2	2	11	193.0000	17.54545
3	3	6	76.0000	12.66667

Dependent: PC ae C38:4	Median Test, Overall Median = 9,11000; PC ae C38:4<br>Independent (grouping) variable: Hashimoto 1 Hypo-no-Hashimoto 2 control 3<br>Chi-Square = 7,571023 df = 2 p = ,0227			
	1	2	3	Total
<= Median: observed	9.00000	6.00000	6.00000	21.00000
expected	12.29268	5.63415	3.07317	
obs.-exp.	-3,29268	0.36585	2.92683	
> Median: observed	15.00000	5.00000	0.00000	20.00000
expected	11.70732	5.36585	2.92683	
obs.-exp.	3.29268	-0,36585	-2,92683	
Total: observed	24.00000	11.00000	6.00000	41.00000

Depend.: PC ae C38:5	Kruskal-Wallis ANOVA by Ranks; PC ae C38:5<br>Independent (grouping) variable: Hashimoto 1 Hypo-no-Hashimoto 2 control 3<br>Kruskal-Wallis test: H (2, N= 41) =6,094534 p =,0475			
	Code	Valid N	Sum of Ranks	Mean Rank
1	1	24	595.5000	24.81250
2	2	11	160.5000	14.59091
3	3	6	105.0000	17.50000

Dependent: PC ae C38:5	Median Test, Overall Median = 13,3000; PC ae C38:5<br>Independent (grouping) variable: Hashimoto 1 Hypo-no-Hashimoto 2 control 3<br>Chi-Square = 4,417632 df = 2 p = ,1098			
	1	2	3	Total
<= Median: observed	9.00000	8.00000	4.000000	21.00000
expected	12.29268	5.63415	3.073171	
obs.-exp.	-3,29268	2.36585	0.926829	
> Median: observed	15.00000	3.00000	2.000000	20.00000
expected	11.70732	5.36585	2.926829	
obs.-exp.	3.29268	-2,36585	-0,926829	
Total: observed	24.00000	11.00000	6.000000	41.00000

Depend.: PC ae C38:6	Kruskal-Wallis ANOVA by Ranks; PC ae C38:6<br>Independent (grouping) variable: Hashimoto 1 Hypo-no-Hashimoto 2 control 3<br>Kruskal-Wallis test: H (2, N= 41) =5,543430 p =,0626			
	Code	Valid N	Sum of Ranks	Mean Rank
1	1	24	586.0000	24.41667
2	2	11	199.5000	18.13636
3	3	6	75.5000	12.58333

Dependent: PC ae C38:6	Median Test, Overall Median = 5,45000; PC ae C38:6<br>Independent (grouping) variable: Hashimoto 1 Hypo-no-Hashimoto 2 control 3<br>Chi-Square = 4,417632 df = 2 p = ,1098			
	1	2	3	Total
<= Median: observed	9.00000	8.00000	4.000000	21.00000
expected	12.29268	5.63415	3.073171	
obs.-exp.	-3,29268	2.36585	0.926829	
> Median: observed	15.00000	3.00000	2.000000	20.00000
expected	11.70732	5.36585	2.926829	
obs.-exp.	3.29268	-2,36585	-0,926829	
Total: observed	24.00000	11.00000	6.000000	41.00000

Depend.: PC ae C40:1	Kruskal-Wallis ANOVA by Ranks; PC ae C40:1<br>Independent (grouping) variable: Hashimoto 1 Hypo-no-Hashimoto 2 control 3<br>Kruskal-Wallis test: H (2, N= 41) =1,042700 p =,5937			
	Code	Valid N	Sum of Ranks	Mean Rank
1	1	24	542.5000	22.60417
2	2	11	204.5000	18.59091
3	3	6	114.0000	19.00000

Dependent: PC ae C40:1	Median Test, Overall Median = ,778000; PC ae C40:1<br>Independent (grouping) variable: Hashimoto 1 Hypo-no-Hashimoto 2 control 3<br>Chi-Square = 2,128391 df = 2 p = ,3450			
	1	2	3	Total
<= Median: observed	10.00000	7.00000	4.000000	21.00000
expected	12.29268	5.63415	3.073171	
obs.-exp.	-2,29268	1.36585	0.926829	
> Median: observed	14.00000	4.00000	2.000000	20.00000
expected	11.70732	5.36585	2.926829	
obs.-exp.	2.29268	-1,36585	-0,926829	
Total: observed	24.00000	11.00000	6.000000	41.00000

Depend.: PC ae C40:2	Kruskal-Wallis ANOVA by Ranks; PC ae C40:2<br>Independent (grouping) variable: Hashimoto 1 Hypo-no-Hashimoto 2 control 3<br>Kruskal-Wallis test: H (2, N= 41) =4,440254 p =,1086			
	Code	Valid N	Sum of Ranks	Mean Rank
1	1	24	556.0000	23.16667
2	2	11	235.0000	21.36364
3	3	6	70.0000	11.66667

Dependent: PC ae C40:2	Median Test, Overall Median = 1,16000; PC ae C40:2<br>Independent (grouping) variable: Hashimoto 1 Hypo-no-Hashimoto 2 control 3<br>Chi-Square = 6,737193 df = 2 p = ,0344			
	1	2	3	Total
<= Median: observed	10.00000	5.00000	6.00000	21.00000
expected	12.29268	5.63415	3.07317	
obs.-exp.	-2,29268	-0,63415	2.92683	
> Median: observed	14.00000	6.00000	0.00000	20.00000
expected	11.70732	5.36585	2.92683	
obs.-exp.	2.29268	0.63415	-2,92683	
Total: observed	24.00000	11.00000	6.00000	41.00000

Depend.: PC ae C40:3	Kruskal-Wallis ANOVA by Ranks; PC ae C40:3<br>Independent (grouping) variable: Hashimoto 1 Hypo-no-Hashimoto 2 control 3<br>Kruskal-Wallis test: H (2, N= 41) =,6817654 p =,7111			
	Code	Valid N	Sum of Ranks	Mean Rank
1	1	24	531.5000	22.14583
2	2	11	204.0000	18.54545
3	3	6	125.5000	20.91667

Dependent: PC ae C40:3	Median Test, Overall Median = ,586000; PC ae C40:3<br>Independent (grouping) variable: Hashimoto 1 Hypo-no-Hashimoto 2 control 3<br>Chi-Square = ,0665584 df = 2 p = ,9673			
	1	2	3	Total
<= Median: observed	12.00000	6.00000	3.000000	21.00000
expected	12.29268	5.63415	3.073171	
obs.-exp.	-0,29268	0.36585	-0,073171	
> Median: observed	12.00000	5.00000	3.000000	20.00000
expected	11.70732	5.36585	2.926829	
obs.-exp.	0.29268	-0,36585	0.073171	
Total: observed	24.00000	11.00000	6.000000	41.00000

Depend.: PC ae C40:4	Kruskal-Wallis ANOVA by Ranks; PC ae C40:4<br>Independent (grouping) variable: Hashimoto 1 Hypo-no-Hashimoto 2 control 3<br>Kruskal-Wallis test: H (2, N= 41) =1,235963 p =,5390			
	Code	Valid N	Sum of Ranks	Mean Rank
1	1	24	537.5000	22.39583
2	2	11	193.5000	17.59091
3	3	6	130.0000	21.66667

Dependent: PC ae C40:4	Median Test, Overall Median = 1,38000; PC ae C40:4<br>Independent (grouping) variable: Hashimoto 1 Hypo-no-Hashimoto 2 control 3<br>Chi-Square = ,0665584 df = 2 p = ,9673			
	1	2	3	Total
<= Median: observed	12.00000	6.00000	3.000000	21.00000
expected	12.29268	5.63415	3.073171	
obs.-exp.	-0,29268	0.36585	-0,073171	
> Median: observed	12.00000	5.00000	3.000000	20.00000
expected	11.70732	5.36585	2.926829	
obs.-exp.	0.29268	-0,36585	0.073171	
Total: observed	24.00000	11.00000	6.000000	41.00000

Depend.: PC ae C40:5	Kruskal-Wallis ANOVA by Ranks; PC ae C40:5<br>Independent (grouping) variable: Hashimoto 1 Hypo-no-Hashimoto 2 control 3<br>Kruskal-Wallis test: H (2, N= 41) =2,244340 p =,3256			
	Code	Valid N	Sum of Ranks	Mean Rank
1	1	24	555.5000	23.14583
2	2	11	183.0000	16.63636
3	3	6	122.5000	20.41667

Dependent: PC ae C40:5	Median Test, Overall Median = 2,08000; PC ae C40:5<br>Independent (grouping) variable: Hashimoto 1 Hypo-no-Hashimoto 2 control 3<br>Chi-Square = ,9610299 df = 2 p = ,6185			
	1	2	3	Total
<= Median: observed	11.00000	7.00000	3.000000	21.00000
expected	12.29268	5.63415	3.073171	
obs.-exp.	-1,29268	1.36585	-0,073171	
> Median: observed	13.00000	4.00000	3.000000	20.00000
expected	11.70732	5.36585	2.926829	
obs.-exp.	1.29268	-1,36585	0.073171	
Total: observed	24.00000	11.00000	6.000000	41.00000

Depend.: PC ae C40:6	Kruskal-Wallis ANOVA by Ranks; PC ae C40:6<br>Independent (grouping) variable: Hashimoto 1 Hypo-no-Hashimoto 2 control 3<br>Kruskal-Wallis test: H (2, N= 41) =1,682768 p =,4311			
	Code	Valid N	Sum of Ranks	Mean Rank
1	1	24	548.0000	22.83333
2	2	11	216.0000	19.63636
3	3	6	97.0000	16.16667

Dependent: PC ae C40:6	Median Test, Overall Median = 2,80000; PC ae C40:6<br>Independent (grouping) variable: Hashimoto 1 Hypo-no-Hashimoto 2 control 3<br>Chi-Square = 7,768110 df = 2 p = ,0206			
	1	2	3	Total
<= Median: observed	8.00000	9.00000	4.000000	21.00000
expected	12.29268	5.63415	3.073171	
obs.-exp.	-4,29268	3.36585	0.926829	
> Median: observed	16.00000	2.00000	2.000000	20.00000
expected	11.70732	5.36585	2.926829	
obs.-exp.	4.29268	-3,36585	-0,926829	
Total: observed	24.00000	11.00000	6.000000	41.00000

Depend.: PC ae C42:0	Kruskal-Wallis ANOVA by Ranks; PC ae C42:0<br>Independent (grouping) variable: Hashimoto 1 Hypo-no-Hashimoto 2 control 3<br>Kruskal-Wallis test: H (2, N= 41) =17,34746 p =,0002			
	Code	Valid N	Sum of Ranks	Mean Rank
1	1	24	596.0000	24.83333
2	2	11	238.0000	21.63636
3	3	6	27.0000	4.50000

Dependent: PC ae C42:0	Median Test, Overall Median = 101,000; PC ae C42:0<br>Independent (grouping) variable: Hashimoto 1 Hypo-no-Hashimoto 2 control 3<br>Chi-Square = 0,000000 df = 2 p = 1,000			
	1	2	3	Total
<= Median: observed	24.00000	11.00000	6.000000	41.00000
expected	24.00000	11.00000	6.000000	
obs.-exp.	0.00000	0.00000	0.000000	
> Median: observed	0.00000	0.00000	0.000000	0.00000
expected	0.00000	0.00000	0.000000	
obs.-exp.	0.00000	0.00000	0.000000	
Total: observed	24.00000	11.00000	6.000000	41.00000

Depend.: PC ae C42:1	Kruskal-Wallis ANOVA by Ranks; PC ae C42:1<br>Independent (grouping) variable: Hashimoto 1 Hypo-no-Hashimoto 2 control 3<br>Kruskal-Wallis test: H (2, N= 41) =,0908708 p =,9556			
	Code	Valid N	Sum of Ranks	Mean Rank
1	1	24	496.5000	20.68750
2	2	11	230.5000	20.95455
3	3	6	134.0000	22.33333

Dependent: PC ae C42:1	Median Test, Overall Median = ,184000; PC ae C42:1<br>Independent (grouping) variable: Hashimoto 1 Hypo-no-Hashimoto 2 control 3<br>Chi-Square = ,9003878 df = 2 p = ,6375			
	1	2	3	Total
<= Median: observed	13.00000	6.00000	2.00000	21.00000
expected	12.29268	5.63415	3.07317	
obs.-exp.	0.70732	0.36585	-1,07317	
> Median: observed	11.00000	5.00000	4.00000	20.00000
expected	11.70732	5.36585	2.92683	
obs.-exp.	-0,70732	-0,36585	1.07317	
Total: observed	24.00000	11.00000	6.00000	41.00000

Depend.: PC ae C42:2	Kruskal-Wallis ANOVA by Ranks; PC ae C42:2<br>Independent (grouping) variable: Hashimoto 1 Hypo-no-Hashimoto 2 control 3<br>Kruskal-Wallis test: H (2, N= 41) =,3718042 p =,8304			
	Code	Valid N	Sum of Ranks	Mean Rank
1	1	24	516.5000	21.52083
2	2	11	235.0000	21.36364
3	3	6	109.5000	18.25000

Dependent: PC ae C42:2	Median Test, Overall Median = ,321000; PC ae C42:2<br>Independent (grouping) variable: Hashimoto 1 Hypo-no-Hashimoto 2 control 3<br>Chi-Square = ,0382685 df = 2 p = ,9810			
	1	2	3	Total
<= Median: observed	13.00000	6.00000	3.000000	22.00000
expected	12.87805	5.90244	3.219512	
obs.-exp.	0.12195	0.09756	-0,219512	
> Median: observed	11.00000	5.00000	3.000000	19.00000
expected	11.12195	5.09756	2.780488	
obs.-exp.	-0,12195	-0,09756	0.219512	
Total: observed	24.00000	11.00000	6.000000	41.00000

Depend.: PC ae C42:3	Kruskal-Wallis ANOVA by Ranks; PC ae C42:3<br>Independent (grouping) variable: Hashimoto 1 Hypo-no-Hashimoto 2 control 3<br>Kruskal-Wallis test: H (2, N= 41) =1,456645 p =,4827			
	Code	Valid N	Sum of Ranks	Mean Rank
1	1	24	502.5000	20.93750
2	2	11	203.5000	18.50000
3	3	6	155.0000	25.83333

Dependent: PC ae C42:3	Median Test, Overall Median = ,467000; PC ae C42:3<br>Independent (grouping) variable: Hashimoto 1 Hypo-no-Hashimoto 2 control 3<br>Chi-Square = 4,917929 df = 2 p = ,0855			
	1	2	3	Total
<= Median: observed	12.00000	8.00000	1.00000	21.00000
expected	12.29268	5.63415	3.07317	
obs.-exp.	-0,29268	2.36585	-2,07317	
> Median: observed	12.00000	3.00000	5.00000	20.00000
expected	11.70732	5.36585	2.92683	
obs.-exp.	0.29268	-2,36585	2.07317	
Total: observed	24.00000	11.00000	6.00000	41.00000

Depend.: PC ae C42:4	Kruskal-Wallis ANOVA by Ranks; PC ae C42:4<br>Independent (grouping) variable: Hashimoto 1 Hypo-no-Hashimoto 2 control 3<br>Kruskal-Wallis test: H (2, N= 41) =1,718818 p =,4234			
	Code	Valid N	Sum of Ranks	Mean Rank
1	1	24	538.0000	22.41667
2	2	11	186.5000	16.95455
3	3	6	136.5000	22.75000

Dependent: PC ae C42:4	Median Test, Overall Median = ,540000; PC ae C42:4<br>Independent (grouping) variable: Hashimoto 1 Hypo-no-Hashimoto 2 control 3<br>Chi-Square = ,0665584 df = 2 p = ,9673			
	1	2	3	Total
<= Median: observed	12.00000	6.00000	3.000000	21.00000
expected	12.29268	5.63415	3.073171	
obs.-exp.	-0,29268	0.36585	-0,073171	
> Median: observed	12.00000	5.00000	3.000000	20.00000
expected	11.70732	5.36585	2.926829	
obs.-exp.	0.29268	-0,36585	0.073171	
Total: observed	24.00000	11.00000	6.000000	41.00000

Depend.: PC ae C42:5	Kruskal-Wallis ANOVA by Ranks; PC ae C42:5<br>Independent (grouping) variable: Hashimoto 1 Hypo-no-Hashimoto 2 control 3<br>Kruskal-Wallis test: H (2, N= 41) =,8297469 p =,6604			
	Code	Valid N	Sum of Ranks	Mean Rank
1	1	24	502.5000	20.93750
2	2	11	210.5000	19.13636
3	3	6	148.0000	24.66667

Dependent: PC ae C42:5	Median Test, Overall Median = 1,42000; PC ae C42:5 Independent (grouping) variable: Hashimoto 1 Hypo-no-Hashimoto 2 control 3 Chi-Square = ,1500512 df = 2 p = ,9277			
	1	2	3	Total
<= Median: observed	14.00000	6.00000	3.000000	23.00000
expected	13.46341	6.17073	3.365854	
obs.-exp.	0.53659	-0,17073	-0,365854	
> Median: observed	10.00000	5.00000	3.000000	18.00000
expected	10.53659	4.82927	2.634146	
obs.-exp.	-0,53659	0.17073	0.365854	
Total: observed	24.00000	11.00000	6.000000	41.00000

Depend.: PC ae C44:3	Kruskal-Wallis ANOVA by Ranks; PC ae C44:3 Independent (grouping) variable: Hashimoto 1 Hypo-no-Hashimoto 2 control 3 Kruskal-Wallis test: H (2, N= 41) =1,219591 p =,5435			
	Code	Valid N	Sum of Ranks	Mean Rank
1	1	24	462.5000	19.27083
2	2	11	260.5000	23.68182
3	3	6	138.0000	23.00000

Dependent: PC ae C44:3	Median Test, Overall Median = ,071000; PC ae C44:3 Independent (grouping) variable: Hashimoto 1 Hypo-no-Hashimoto 2 control 3 Chi-Square = ,5409598 df = 2 p = ,7630			
	1	2	3	Total
<= Median: observed	14.00000	5.00000	3.000000	22.00000
expected	12.87805	5.90244	3.219512	
obs.-exp.	1.12195	-0,90244	-0,219512	
> Median: observed	10.00000	6.00000	3.000000	19.00000
expected	11.12195	5.09756	2.780488	
obs.-exp.	-1,12195	0.90244	0.219512	
Total: observed	24.00000	11.00000	6.000000	41.00000

Depend.: PC ae C44:4	Kruskal-Wallis ANOVA by Ranks; PC ae C44:4 Independent (grouping) variable: Hashimoto 1 Hypo-no-Hashimoto 2 control 3 Kruskal-Wallis test: H (2, N= 41) =1,563043 p =,4577			
	Code	Valid N	Sum of Ranks	Mean Rank
1	1	24	472.0000	19.66667
2	2	11	230.0000	20.90909
3	3	6	159.0000	26.50000

Dependent: PC ae C44:4	Median Test, Overall Median = ,232000; PC ae C44:4<br>Independent (grouping) variable: Hashimoto 1 Hypo-no-Hashimoto 2 control 3<br>Chi-Square = 1,400685 df = 2 p = ,4964			
	1	2	3	Total
<= Median: observed	14.00000	5.00000	2.00000	21.00000
expected	12.29268	5.63415	3.07317	
obs.-exp.	1.70732	-0,63415	-1,07317	
> Median: observed	10.00000	6.00000	4.00000	20.00000
expected	11.70732	5.36585	2.92683	
obs.-exp.	-1,70732	0.63415	1.07317	
Total: observed	24.00000	11.00000	6.00000	41.00000

Depend.: PC ae C44:5	Kruskal-Wallis ANOVA by Ranks; PC ae C44:5<br>Independent (grouping) variable: Hashimoto 1 Hypo-no-Hashimoto 2 control 3<br>Kruskal-Wallis test: H (2, N= 41) =1,568241 p =,4565			
	Code	Valid N	Sum of Ranks	Mean Rank
1	1	24	491.0000	20.45833
2	2	11	211.0000	19.18182
3	3	6	159.0000	26.50000

Dependent: PC ae C44:5	Median Test, Overall Median = 1,23000; PC ae C44:5<br>Independent (grouping) variable: Hashimoto 1 Hypo-no-Hashimoto 2 control 3<br>Chi-Square = 1,439711 df = 2 p = ,4868			
	1	2	3	Total
<= Median: observed	13.00000	7.00000	2.00000	22.00000
expected	12.87805	5.90244	3.21951	
obs.-exp.	0.12195	1.09756	-1,21951	
> Median: observed	11.00000	4.00000	4.00000	19.00000
expected	11.12195	5.09756	2.78049	
obs.-exp.	-0,12195	-1,09756	1.21951	
Total: observed	24.00000	11.00000	6.00000	41.00000

Depend.: PC ae C44:6	Kruskal-Wallis ANOVA by Ranks; PC ae C44:6<br>Independent (grouping) variable: Hashimoto 1 Hypo-no-Hashimoto 2 control 3<br>Kruskal-Wallis test: H (2, N= 41) =1,356185 p =,5076			
	Code	Valid N	Sum of Ranks	Mean Rank
1	1	24	471.5000	19.64583
2	2	11	233.5000	21.22727
3	3	6	156.0000	26.00000

Dependent: PC ae C44:6	Median Test, Overall Median = ,755000; PC ae C44:6<br>Independent (grouping) variable: Hashimoto 1 Hypo-no-Hashimoto 2 control 3<br>Chi-Square = 1,400685 df = 2 p = ,4964			
	1	2	3	Total
<= Median: observed	14.00000	5.00000	2.00000	21.00000
expected	12.29268	5.63415	3.07317	
obs.-exp.	1.70732	-0,63415	-1,07317	
> Median: observed	10.00000	6.00000	4.00000	20.00000
expected	11.70732	5.36585	2.92683	
obs.-exp.	-1,70732	0.63415	1.07317	
Total: observed	24.00000	11.00000	6.00000	41.00000

Depend.: SM (OH) C14:1	Kruskal-Wallis ANOVA by Ranks; SM (OH) C14:1<br>Independent (grouping) variable: Hashimoto 1 Hypo-no-Hashimoto 2 control 3<br>Kruskal-Wallis test: H (2, N= 41) =3,873297 p =,1442			
	Code	Valid N	Sum of Ranks	Mean Rank
1	1	24	558.0000	23.25000
2	2	11	228.0000	20.72727
3	3	6	75.0000	12.50000

Dependent: SM(OH) C14:1	Median Test, Overall Median = 4,29000; SM(OH) C14:1<br>Independent (grouping) variable: Hashimoto 1 Hypo-no-Hashimoto 2 control 3<br>Chi-Square = ,9003878 df = 2 p = ,6375			
	1	2	3	Total
<= Median: observed	11.00000	6.00000	4.000000	21.00000
expected	12.29268	5.63415	3.073171	
obs.-exp.	-1,29268	0.36585	0.926829	
> Median: observed	13.00000	5.00000	2.000000	20.00000
expected	11.70732	5.36585	2.926829	
obs.-exp.	1.29268	-0,36585	-0,926829	
Total: observed	24.00000	11.00000	6.000000	41.00000

Depend.: SM (OH) C16:1	Kruskal-Wallis ANOVA by Ranks; SM (OH) C16:1<br>Independent (grouping) variable: Hashimoto 1 Hypo-no-Hashimoto 2 control 3<br>Kruskal-Wallis test: H (2, N= 41) =4,371151 p =,1124			
	Code	Valid N	Sum of Ranks	Mean Rank
1	1	24	567.0000	23.62500
2	2	11	220.0000	20.00000
3	3	6	74.0000	12.33333

Dependent: SM(OH) C16:1	Median Test, Overall Median = 2,15000; SM(OH) C16:1<br>Independent (grouping) variable: Hashimoto 1 Hypo-no-Hashimoto 2 control 3<br>Chi-Square = 3,401876 df = 2 p = ,1825			
	1	2	3	Total
<= Median: observed	10.00000	6.00000	5.00000	21.00000
expected	12.29268	5.63415	3.07317	
obs.-exp.	-2,29268	0.36585	1.92683	
> Median: observed	14.00000	5.00000	1.00000	20.00000
expected	11.70732	5.36585	2.92683	
obs.-exp.	2.29268	-0,36585	-1,92683	
Total: observed	24.00000	11.00000	6.00000	41.00000

Depend.: SM (OH) C22:1	Kruskal-Wallis ANOVA by Ranks; SM (OH) C22:1<br>Independent (grouping) variable: Hashimoto 1 Hypo-no-Hashimoto 2 control 3<br>Kruskal-Wallis test: H (2, N= 41) =1,843577 p =,3978			
	Code	Valid N	Sum of Ranks	Mean Rank
1	1	24	538.0000	22.41667
2	2	11	233.0000	21.18182
3	3	6	90.0000	15.00000

Dependent: SM(OH) C22:1	Median Test, Overall Median = 7,75000; SM(OH) C22:1<br>Independent (grouping) variable: Hashimoto 1 Hypo-no-Hashimoto 2 control 3<br>Chi-Square = ,9003878 df = 2 p = ,6375			
	1	2	3	Total
<= Median: observed	11.00000	6.00000	4.000000	21.00000
expected	12.29268	5.63415	3.073171	
obs.-exp.	-1,29268	0.36585	0.926829	
> Median: observed	13.00000	5.00000	2.000000	20.00000
expected	11.70732	5.36585	2.926829	
obs.-exp.	1.29268	-0,36585	-0,926829	
Total: observed	24.00000	11.00000	6.000000	41.00000

Depend.: SM (OH) C22:2	Kruskal-Wallis ANOVA by Ranks; SM (OH) C22:2<br>Independent (grouping) variable: Hashimoto 1 Hypo-no-Hashimoto 2 control 3<br>Kruskal-Wallis test: H (2, N= 41) =4,394038 p =,1111			
	Code	Valid N	Sum of Ranks	Mean Rank
1	1	24	562.5000	23.43750
2	2	11	226.5000	20.59091
3	3	6	72.0000	12.00000

Dependent: SM(OH) C22:2	Median Test, Overall Median = 6,71000; SM(OH) C22:2<br>Independent (grouping) variable: Hashimoto 1 Hypo-no-Hashimoto 2 control 3<br>Chi-Square = 3,401876 df = 2 p = ,1825			
	1	2	3	Total
<= Median: observed	10.00000	6.00000	5.00000	21.00000
expected	12.29268	5.63415	3.07317	
obs.-exp.	-2,29268	0.36585	1.92683	
> Median: observed	14.00000	5.00000	1.00000	20.00000
expected	11.70732	5.36585	2.92683	
obs.-exp.	2.29268	-0,36585	-1,92683	
Total: observed	24.00000	11.00000	6.00000	41.00000

Depend.: SM (OH) C24:1	Kruskal-Wallis ANOVA by Ranks; SM (OH) C24:1<br>Independent (grouping) variable: Hashimoto 1 Hypo-no-Hashimoto 2 control 3<br>Kruskal-Wallis test: H (2, N= 41) =,8843730 p =,6426			
	Code	Valid N	Sum of Ranks	Mean Rank
1	1	24	539.5000	22.47917
2	2	11	209.0000	19.00000
3	3	6	112.5000	18.75000

Dependent: SM(OH) C24:1	Median Test, Overall Median = ,705000; SM(OH) C24:1<br>Independent (grouping) variable: Hashimoto 1 Hypo-no-Hashimoto 2 control 3<br>Chi-Square = ,9610299 df = 2 p = ,6185			
	1	2	3	Total
<= Median: observed	11.00000	7.00000	3.000000	21.00000
expected	12.29268	5.63415	3.073171	
obs.-exp.	-1,29268	1.36585	-0,073171	
> Median: observed	13.00000	4.00000	3.000000	20.00000
expected	11.70732	5.36585	2.926829	
obs.-exp.	1.29268	-1,36585	0.073171	
Total: observed	24.00000	11.00000	6.000000	41.00000

Depend.: SM C16:0	Kruskal-Wallis ANOVA by Ranks; SM C16:0<br>Independent (grouping) variable: Hashimoto 1 Hypo-no-Hashimoto 2 control 3<br>Kruskal-Wallis test: H (2, N= 41) =4,124887 p =,1271			
	Code	Valid N	Sum of Ranks	Mean Rank
1	1	24	576.0000	24.00000
2	2	11	201.0000	18.27273
3	3	6	84.0000	14.00000

Dependent: SM C16:0	Median Test, Overall Median = 90,2000; SM C16:0<br>Independent (grouping) variable: Hashimoto 1 Hypo-no-Hashimoto 2 control 3<br>Chi-Square = 2,128391 df = 2 p = ,3450			
	1	2	3	Total
<= Median: observed	10.00000	7.00000	4.000000	21.00000
expected	12.29268	5.63415	3.073171	
obs.-exp.	-2,29268	1.36585	0.926829	
> Median: observed	14.00000	4.00000	2.000000	20.00000
expected	11.70732	5.36585	2.926829	
obs.-exp.	2.29268	-1,36585	-0,926829	
Total: observed	24.00000	11.00000	6.000000	41.00000

	Kruskal-Wallis ANOVA by Ranks; SM C16:1<br>Independent (grouping) variable: Hashimoto 1 Hypo-no-Hashimoto 2 control 3<br>Kruskal-Wallis test: H (2, N= 41) =2,679622 p =,2619			
Depend.: SM C16:1	Code	Valid N	Sum of Ranks	Mean Rank
1	1	24	551.5000	22.97917
2	2	11	225.0000	20.45455
3	3	6	84.5000	14.08333

Dependent: SM C16:1	Median Test, Overall Median = 11,6000; SM C16:1<br>Independent (grouping) variable: Hashimoto 1 Hypo-no-Hashimoto 2 control 3<br>Chi-Square = ,9003878 df = 2 p = ,6375			
	1	2	3	Total
<= Median: observed	11.00000	6.00000	4.000000	21.00000
expected	12.29268	5.63415	3.073171	
obs.-exp.	-1,29268	0.36585	0.926829	
> Median: observed	13.00000	5.00000	2.000000	20.00000
expected	11.70732	5.36585	2.926829	
obs.-exp.	1.29268	-0,36585	-0,926829	
Total: observed	24.00000	11.00000	6.000000	41.00000

	Kruskal-Wallis ANOVA by Ranks; SM C18:0<br>Independent (grouping) variable: Hashimoto 1 Hypo-no-Hashimoto 2 control 3<br>Kruskal-Wallis test: H (2, N= 41) =5,723040 p =,0572			
Depend.: SM C18:0	Code	Valid N	Sum of Ranks	Mean Rank
1	1	24	575.0000	23.95833
2	2	11	220.0000	20.00000
3	3	6	66.0000	11.00000

Dependent: SM C18:0	Median Test, Overall Median = 15,6000; SM C18:0<br>Independent (grouping) variable: Hashimoto 1 Hypo-no-Hashimoto 2 control 3<br>Chi-Square = 4,963411 df = 2 p = ,0836			
	1	2	3	Total
<= Median: observed	9.00000	7.00000	5.00000	21.00000
expected	12.29268	5.63415	3.07317	
obs.-exp.	-3,29268	1.36585	1.92683	
> Median: observed	15.00000	4.00000	1.00000	20.00000
expected	11.70732	5.36585	2.92683	
obs.-exp.	3.29268	-1,36585	-1,92683	
Total: observed	24.00000	11.00000	6.00000	41.00000

Depend.: SM C18:1	Kruskal-Wallis ANOVA by Ranks; SM C18:1<br>Independent (grouping) variable: Hashimoto 1 Hypo-no-Hashimoto 2 control 3<br>Kruskal-Wallis test: H (2, N= 41) =5,970296 p =,0505			
	Code	Valid N	Sum of Ranks	Mean Rank
1	1	24	581.0000	24.20833
2	2	11	213.0000	19.36364
3	3	6	67.0000	11.16667

Dependent: SM C18:1	Median Test, Overall Median = 7,09000; SM C18:1<br>Independent (grouping) variable: Hashimoto 1 Hypo-no-Hashimoto 2 control 3<br>Chi-Square = 3,401876 df = 2 p = ,1825			
	1	2	3	Total
<= Median: observed	10.00000	6.00000	5.00000	21.00000
expected	12.29268	5.63415	3.07317	
obs.-exp.	-2,29268	0.36585	1.92683	
> Median: observed	14.00000	5.00000	1.00000	20.00000
expected	11.70732	5.36585	2.92683	
obs.-exp.	2.29268	-0,36585	-1,92683	
Total: observed	24.00000	11.00000	6.00000	41.00000

Depend.: SM C20:2	Kruskal-Wallis ANOVA by Ranks; SM C20:2<br>Independent (grouping) variable: Hashimoto 1 Hypo-no-Hashimoto 2 control 3<br>Kruskal-Wallis test: H (2, N= 41) =,6545346 p =,7209			
	Code	Valid N	Sum of Ranks	Mean Rank
1	1	24	513.5000	21.39583
2	2	11	243.0000	22.09091
3	3	6	104.5000	17.41667

Dependent: SM C20:2	Median Test, Overall Median = ,187000; SM C20:2<br>Independent (grouping) variable: Hashimoto 1 Hypo-no-Hashimoto 2 control 3<br>Chi-Square = ,7085236 df = 2 p = ,7017			
	1	2	3	Total
<= Median: observed	13.00000	5.00000	4.000000	22.00000
expected	12.87805	5.90244	3.219512	
obs.-exp.	0.12195	-0,90244	0.780488	
> Median: observed	11.00000	6.00000	2.000000	19.00000
expected	11.12195	5.09756	2.780488	
obs.-exp.	-0,12195	0.90244	-0,780488	
Total: observed	24.00000	11.00000	6.000000	41.00000

Depend.: SM C22:3	Kruskal-Wallis ANOVA by Ranks; SM C22:3<br>Independent (grouping) variable: Hashimoto 1 Hypo-no-Hashimoto 2 control 3<br>Kruskal-Wallis test: H (2, N= 41) =0,000000 p =1,000			
	Code	Valid N	Sum of Ranks	Mean Rank
1	1	24	504.0000	21.00000
2	2	11	231.0000	21.00000
3	3	6	126.0000	21.00000

Dependent: SM C22:3	Median Test, Overall Median = 101,000; SM C22:3<br>Independent (grouping) variable: Hashimoto 1 Hypo-no-Hashimoto 2 control 3<br>Chi-Square = 0,000000 df = 2 p = 1,000			
	1	2	3	Total
<= Median: observed	24.00000	11.00000	6.000000	41.00000
expected	24.00000	11.00000	6.000000	
obs.-exp.	0.00000	0.00000	0.000000	
> Median: observed	0.00000	0.00000	0.000000	0.00000
expected	0.00000	0.00000	0.000000	
obs.-exp.	0.00000	0.00000	0.000000	
Total: observed	24.00000	11.00000	6.000000	41.00000

Depend.: SM C24:0	Kruskal-Wallis ANOVA by Ranks; SM C24:0<br>Independent (grouping) variable: Hashimoto 1 Hypo-no-Hashimoto 2 control 3<br>Kruskal-Wallis test: H (2, N= 41) =,2834391 p =,8679			
	Code	Valid N	Sum of Ranks	Mean Rank
1	1	24	517.0000	21.54167
2	2	11	213.0000	19.36364
3	3	6	131.0000	21.83333

Dependent: SM C24:0	Median Test, Overall Median = 11,8000; SM C24:0<br>Independent (grouping) variable: Hashimoto 1 Hypo-no-Hashimoto 2 control 3<br>Chi-Square = ,2333243 df = 2 p = ,8899			
	1	2	3	Total
<= Median: observed	13.00000	5.00000	3.000000	21.00000
expected	12.29268	5.63415	3.073171	
obs.-exp.	0.70732	-0,63415	-0,073171	
> Median: observed	11.00000	6.00000	3.000000	20.00000
expected	11.70732	5.36585	2.926829	
obs.-exp.	-0,70732	0.63415	0.073171	
Total: observed	24.00000	11.00000	6.000000	41.00000

Depend.: SM C24:1	Kruskal-Wallis ANOVA by Ranks; SM C24:1<br>Independent (grouping) variable: Hashimoto 1 Hypo-no-Hashimoto 2 control 3<br>Kruskal-Wallis test: H (2, N= 41) =2,744651 p =,2535			
	Code	Valid N	Sum of Ranks	Mean Rank
1	1	24	557.0000	23.20833
2	2	11	217.5000	19.77273
3	3	6	86.5000	14.41667

Dependent: SM C24:1	Median Test, Overall Median = 37,6000; SM C24:1<br>Independent (grouping) variable: Hashimoto 1 Hypo-no-Hashimoto 2 control 3<br>Chi-Square = ,9003878 df = 2 p = ,6375			
	1	2	3	Total
<= Median: observed	11.00000	6.00000	4.000000	21.00000
expected	12.29268	5.63415	3.073171	
obs.-exp.	-1,29268	0.36585	0.926829	
> Median: observed	13.00000	5.00000	2.000000	20.00000
expected	11.70732	5.36585	2.926829	
obs.-exp.	1.29268	-0,36585	-0,926829	
Total: observed	24.00000	11.00000	6.000000	41.00000

Depend.: SM C26:0	Kruskal-Wallis ANOVA by Ranks; SM C26:0<br>Independent (grouping) variable: Hashimoto 1 Hypo-no-Hashimoto 2 control 3<br>Kruskal-Wallis test: H (2, N= 41) =,1326104 p =,9358			
	Code	Valid N	Sum of Ranks	Mean Rank
1	1	24	516.5000	21.52083
2	2	11	226.5000	20.59091
3	3	6	118.0000	19.66667

Dependent: SM C26:0	Median Test, Overall Median = ,089000; SM C26:0<br>Independent (grouping) variable: Hashimoto 1 Hypo-no-Hashimoto 2 control 3<br>Chi-Square = ,0665584 df = 2 p = ,9673			
	1	2	3	Total
<= Median: observed	12.00000	6.00000	3.000000	21.00000
expected	12.29268	5.63415	3.073171	
obs.-exp.	-0,29268	0.36585	-0,073171	
> Median: observed	12.00000	5.00000	3.000000	20.00000
expected	11.70732	5.36585	2.926829	
obs.-exp.	0.29268	-0,36585	0.073171	
Total: observed	24.00000	11.00000	6.000000	41.00000

Depend.: SM C26:1	Kruskal-Wallis ANOVA by Ranks; SM C26:1<br>Independent (grouping) variable: Hashimoto 1 Hypo-no-Hashimoto 2 control 3<br>Kruskal-Wallis test: H (2, N= 41) =,8068047 p =,6680			
	Code	Valid N	Sum of Ranks	Mean Rank
1	1	24	534.0000	22.25000
2	2	11	221.5000	20.13636
3	3	6	105.5000	17.58333

Dependent: SM C26:1	Median Test, Overall Median = ,223000; SM C26:1<br>Independent (grouping) variable: Hashimoto 1 Hypo-no-Hashimoto 2 control 3<br>Chi-Square = ,9003878 df = 2 p = ,6375			
	1	2	3	Total
<= Median: observed	11.00000	6.00000	4.000000	21.00000
expected	12.29268	5.63415	3.073171	
obs.-exp.	-1,29268	0.36585	0.926829	
> Median: observed	13.00000	5.00000	2.000000	20.00000
expected	11.70732	5.36585	2.926829	
obs.-exp.	1.29268	-0,36585	-0,926829	
Total: observed	24.00000	11.00000	6.000000	41.00000

Kruskal-Wallis ANOVA by Ranks; Age (years)
Independent (grouping) variable: Hashimoto 1 Hypo-no-Hashimoto 2 control 3
Kruskal-Wallis test: H (2, N= 41) =2,001866 p =,3675

Depend.: Age (years)	Code	Valid N	Sum of Ranks	Mean Rank
1	1	24	556.5000	23.18750
2	2	11	201.0000	18.27273
3	3	6	103.5000	17.25000

Median Test, Overall Median = 26,0000; Age (years)
Independent (grouping) variable: Hashimoto 1 Hypo-no-Hashimoto 2 control 3
Chi-Square = 3,401876 df = 2 p = ,1825

Dependent: Age (years)	1	2	3	Total
<= Median: observed	10.00000	6.00000	5.00000	21.00000
expected	12.29268	5.63415	3.07317	
obs.-exp.	-2,29268	0.36585	1.92683	
> Median: observed	14.00000	5.00000	1.00000	20.00000
expected	11.70732	5.36585	2.92683	
obs.-exp.	2.29268	-0,36585	-1,92683	
Total: observed	24.00000	11.00000	6.00000	41.00000

Kruskal-Wallis ANOVA by Ranks; BMI (kg/m2)
Independent (grouping) variable: Hashimoto 1 Hypo-no-Hashimoto 2 control 3
Kruskal-Wallis test: H (2, N= 41) =1,394230 p =,4980

Depend.: BMI (kg/m2)	Code	Valid N	Sum of Ranks	Mean Rank
1	1	24	527.0000	21.95833
2	2	11	240.0000	21.81818
3	3	6	94.0000	15.66667

Dependent: BMI (kg/m2)	Median Test, Overall Median = 23,2000; BMI (kg/m2)<br>Independent (grouping) variable: Hashimoto 1 Hypo-no-Hashimoto 2 control 3<br>Chi-Square = 3,462518 df = 2 p = ,1771			
	1	2	3	Total
<= Median: observed	12.00000	4.00000	5.00000	21.00000
expected	12.29268	5.63415	3.07317	
obs.-exp.	-0,29268	-1,63415	1.92683	
> Median: observed	12.00000	7.00000	1.00000	20.00000
expected	11.70732	5.36585	2.92683	
obs.-exp.	0.29268	1.63415	-1,92683	
Total: observed	24.00000	11.00000	6.00000	41.00000

Depend.: Hypothyroidism (years)	Kruskal-Wallis ANOVA by Ranks; Hypothyroidism (years)<br>Independent (grouping) variable: Hashimoto 1 Hypo-no-Hashimoto 2 control 3<br>Kruskal-Wallis test: H (2, N= 35) =0,000000 p =1,000			
	Code	Valid N	Sum of Ranks	Mean Rank
1	1	24	473.5000	19.72917
2	2	11	156.5000	14.22727
3	3	0		15.66667

Dependent: Hypothyroidism (years)	Median Test, Overall Median = 4,00000; Hypothyroidism (years)<br>Independent (grouping) variable: Hashimoto 1 Hypo-no-Hashimoto 2 control 3<br>Chi-Square = 2,913201 df = 2 p = ,2330			
	1	2	3	Total
<= Median: observed	10.00000	8.00000	0.00	18.00000
expected	12.34286	5.65714	0.00	
obs.-exp.	-2,34286	2.34286	0.00	
> Median: observed	14.00000	3.00000	0.00	17.00000
expected	11.65714	5.34286	0.00	
obs.-exp.	2.34286	-2,34286	0.00	
Total: observed	24.00000	11.00000	0.00	35.00000

Depend.: lT4 dose	Kruskal-Wallis ANOVA by Ranks; lT4 dose<br>Independent (grouping) variable: Hashimoto 1 Hypo-no-Hashimoto 2 control 3<br>Kruskal-Wallis test: H (2, N= 35) =0,000000 p =1,000			
	Code	Valid N	Sum of Ranks	Mean Rank
1	1	24	511.5000	21.31250
2	2	11	118.5000	10.77273
3	3	0		15.66667

Dependent: IT4 dose	Median Test, Overall Median = 62,5000; IT4 dose<br>Independent (grouping) variable: Hashimoto 1 Hypo-no-Hashimoto 2 control 3<br>Chi-Square = 10,00990 df = 2 p = ,0067			
	1	2	3	Total
<= Median: observed	8.00000	10.00000	0.00	18.00000
expected	12.34286	5.65714	0.00	
obs.-exp.	-4,34286	4.34286	0.00	
> Median: observed	16.00000	1.00000	0.00	17.00000
expected	11.65714	5.34286	0.00	
obs.-exp.	4.34286	-4,34286	0.00	
Total: observed	24.00000	11.00000	0.00	35.00000

Depend.: TSH (uIU/mL)	Kruskal-Wallis ANOVA by Ranks; TSH (uIU/mL)<br>Independent (grouping) variable: Hashimoto 1 Hypo-no-Hashimoto 2 control 3<br>Kruskal-Wallis test: H (2, N= 41) =2,951331 p =,2286			
	Code	Valid N	Sum of Ranks	Mean Rank
1	1	24	567.0000	23.62500
2	2	11	200.0000	18.18182
3	3	6	94.0000	15.66667

Dependent: TSH(uIU/mL)	Median Test, Overall Median = 1,78000; TSH(uIU/mL)<br>Independent (grouping) variable: Hashimoto 1 Hypo-no-Hashimoto 2 control 3<br>Chi-Square = 4,963411 df = 2 p = ,0836			
	1	2	3	Total
<= Median: observed	9.00000	7.00000	5.00000	21.00000
expected	12.29268	5.63415	3.07317	
obs.-exp.	-3,29268	1.36585	1.92683	
> Median: observed	15.00000	4.00000	1.00000	20.00000
expected	11.70732	5.36585	2.92683	
obs.-exp.	3.29268	-1,36585	-1,92683	
Total: observed	24.00000	11.00000	6.00000	41.00000

Depend.: fT4 (ng/dL)	Kruskal-Wallis ANOVA by Ranks; fT4 (ng/dL)<br>Independent (grouping) variable: Hashimoto 1 Hypo-no-Hashimoto 2 control 3<br>Kruskal-Wallis test: H (2, N= 41) =3,308598 p =,1912			
	Code	Valid N	Sum of Ranks	Mean Rank
1	1	24	435.5000	18.14583
2	2	11	272.0000	24.72727
3	3	6	153.5000	25.58333

Median Test, Overall Median = 1,21000; fT4 (ng/dL)
Independent (grouping) variable: Hashimoto 1 Hypo-no-Hashimoto 2 control 3
Chi-Square = 5,232746 df = 2 p = ,0731

Dependent: fT4 (ng/dL)			1	2	3	Total
<= Median:	observed		16.00000	5.00000	1.00000	22.00000
		expected	12.87805	5.90244	3.21951	
		obs.-exp.	3.12195	-0,90244	-2,21951	
> Median: observed			8.00000	6.00000	5.00000	19.00000
		expected	11.12195	5.09756	2.78049	
		obs.-exp.	-3,12195	0.90244	2.21951	
	Total:	observed	24.00000	11.00000	6.00000	41.00000

Kruskal-Wallis ANOVA by Ranks; fT3 (pg/mL)
Independent (grouping) variable: Hashimoto 1 Hypo-no-Hashimoto 2 control 3
Kruskal-Wallis test: H (2, N= 41) =1,628421 p =,4430

Depend.: fT3 (pg/mL)	Code	Valid N	Sum of Ranks	Mean Rank
1	1	24	458.5000	19.10417
2	2	11	250.5000	22.77273
3	3	6	152.0000	25.33333

Median Test, Overall Median = 3,11000; fT3 (pg/mL)
Independent (grouping) variable: Hashimoto 1 Hypo-no-Hashimoto 2 control 3
Chi-Square = 1,211215 df = 2 p = ,5457

Dependent: fT3 (pg/mL)	1	2	3	Total
<= Median: observed	14.00000	6.00000	2.00000	22.00000
expected	12.87805	5.90244	3.21951	
obs.-exp.	1.12195	0.09756	-1,21951	
> Median: observed	10.00000	5.00000	4.00000	19.00000
expected	11.12195	5.09756	2.78049	
obs.-exp.	-1,12195	-0,09756	1.21951	
Total: observed	24.00000	11.00000	6.00000	41.00000

Kruskal-Wallis ANOVA by Ranks; anty-TPO levels above the upper limit
Independent (grouping) variable: Hashimoto 1 Hypo-no-Hashimoto 2 control 3
Kruskal-Wallis test: H (2, N= 24) =0,000000 p =1,000

Depend.: anty-TPO levels above the upper limit	Code	Valid N	Sum of Ranks	Mean Rank
1	1	24	300.0000	12.50000
2	2	0		22.77273
3	3	0		25.33333

Dependent: anty-TPO levels above the upper limit	Median Test, Overall Median = 12,8000; anty-TPO levels above the upper limit<br>Independent (grouping) variable: Hashimoto 1 Hypo-no-Hashimoto 2 control 3<br>Chi-Square = 0,000000 df = 2 p = 1,000			
	1	2	3	Total
<= Median: observed	12.00000	0.00	0.00	12.00000
expected	12.00000	0.00	0.00	
obs.-exp.	0.00000	0.00	0.00	
> Median: observed	12.00000	0.00	0.00	12.00000
expected	12.00000	0.00	0.00	
obs.-exp.	0.00000	0.00	0.00	
Total: observed	24.00000	0.00	0.00	24.00000

Depend.: Total cholesterol (mg/dL)	Kruskal-Wallis ANOVA by Ranks; Total cholesterol (mg/dL)<br>Independent (grouping) variable: Hashimoto 1 Hypo-no-Hashimoto 2 control 3<br>Kruskal-Wallis test: H (2, N= 41) =4,105231 p =,1284			
	Code	Valid N	Sum of Ranks	Mean Rank
1	1	24	574.5000	23.93750
2	2	11	204.0000	18.54545
3	3	6	82.5000	13.75000

Dependent: Total cholesterol (mg/dL)	Median Test, Overall Median = 168,000; Total cholesterol (mg/dL)<br>Independent (grouping) variable: Hashimoto 1 Hypo-no-Hashimoto 2 control 3<br>Chi-Square = 7,586183 df = 2 p = ,0225			
	1	2	3	Total
<= Median: observed	8.00000	8.00000	5.00000	21.00000
expected	12.29268	5.63415	3.07317	
obs.-exp.	-4,29268	2.36585	1.92683	
> Median: observed	16.00000	3.00000	1.00000	20.00000
expected	11.70732	5.36585	2.92683	
obs.-exp.	4.29268	-2,36585	-1,92683	
Total: observed	24.00000	11.00000	6.00000	41.00000

Depend.: HDL cholesterol (mg/dL)	Kruskal-Wallis ANOVA by Ranks; HDL cholesterol (mg/dL)<br>Independent (grouping) variable: Hashimoto 1 Hypo-no-Hashimoto 2 control 3<br>Kruskal-Wallis test: H (2, N= 41) =4,820234 p =,0898			
	Code	Valid N	Sum of Ranks	Mean Rank
1	1	24	579.0000	24.12500
2	2	11	204.5000	18.59091
3	3	6	77.5000	12.91667

Dependent: HDL cholesterol (mg/dL)	Median Test, Overall Median = 58,0000; HDL cholesterol (mg/dL)<br>Independent (grouping) variable: Hashimoto 1 Hypo-no-Hashimoto 2 control 3<br>Chi-Square = 7,586183 df = 2 p = ,0225			
	1	2	3	Total
<= Median: observed	8.00000	8.00000	5.00000	21.00000
expected	12.29268	5.63415	3.07317	
obs.-exp.	-4,29268	2.36585	1.92683	
> Median: observed	16.00000	3.00000	1.00000	20.00000
expected	11.70732	5.36585	2.92683	
obs.-exp.	4.29268	-2,36585	-1,92683	
Total: observed	24.00000	11.00000	6.00000	41.00000

Depend.: LDL cholesterol (mg/dL)	Kruskal-Wallis ANOVA by Ranks; LDL cholesterol (mg/dL)<br>Independent (grouping) variable: Hashimoto 1 Hypo-no-Hashimoto 2 control 3<br>Kruskal-Wallis test: H (2, N= 41) =,1102600 p =,9464			
	Code	Valid N	Sum of Ranks	Mean Rank
1	1	24	510.0000	21.25000
2	2	11	234.0000	21.27273
3	3	6	117.0000	19.50000

Dependent: LDL cholesterol (mg/dL)	Median Test, Overall Median = 92,4000; LDL cholesterol (mg/dL)<br>Independent (grouping) variable: Hashimoto 1 Hypo-no-Hashimoto 2 control 3<br>Chi-Square = ,7336219 df = 2 p = ,6929			
	1	2	3	Total
<= Median: observed	12.00000	5.00000	4.000000	21.00000
expected	12.29268	5.63415	3.073171	
obs.-exp.	-0,29268	-0,63415	0.926829	
> Median: observed	12.00000	6.00000	2.000000	20.00000
expected	11.70732	5.36585	2.926829	
obs.-exp.	0.29268	0.63415	-0,926829	
Total: observed	24.00000	11.00000	6.000000	41.00000

Depend.: Triglycerides	Kruskal-Wallis ANOVA by Ranks; Triglycerides<br>Independent (grouping) variable: Hashimoto 1 Hypo-no-Hashimoto 2 control 3<br>Kruskal-Wallis test: H (2, N= 41) =3,684938 p =,1584			
	Code	Valid N	Sum of Ranks	Mean Rank
1	1	24	541.5000	22.56250
2	2	11	245.5000	22.31818
3	3	6	74.0000	12.33333

Dependent: Triglycerides	Median Test, Overall Median = 82,4300; Triglycerides<br>Independent (grouping) variable: Hashimoto 1 Hypo-no-Hashimoto 2 control 3<br>Chi-Square = 2,901578 df = 2 p = ,2344			
	1	2	3	Total
<= Median: observed	11.00000	5.00000	5.00000	21.00000
expected	12.29268	5.63415	3.07317	
obs.-exp.	-1,29268	-0,63415	1.92683	
> Median: observed	13.00000	6.00000	1.00000	20.00000
expected	11.70732	5.36585	2.92683	
obs.-exp.	1.29268	0.63415	-1,92683	
Total: observed	24.00000	11.00000	6.00000	41.00000

Depend.: AIP	Kruskal-Wallis ANOVA by Ranks; AIP<br>Independent (grouping) variable: Hashimoto 1 Hypo-no-Hashimoto 2 control 3<br>Kruskal-Wallis test: H (2, N= 41) =2,731200 p =,2552			
	Code	Valid N	Sum of Ranks	Mean Rank
1	1	24	497.5000	20.72917
2	2	11	274.0000	24.90909
3	3	6	89.5000	14.91667

Dependent: AIP	Median Test, Overall Median = ,148939; AIP<br>Independent (grouping) variable: Hashimoto 1 Hypo-no-Hashimoto 2 control 3<br>Chi-Square = 5,084695 df = 2 p = ,0787			
	1	2	3	Total
<= Median: observed	13.00000	3.00000	5.00000	21.00000
expected	12.29268	5.63415	3.07317	
obs.-exp.	0.70732	-2,63415	1.92683	
> Median: observed	11.00000	8.00000	1.00000	20.00000
expected	11.70732	5.36585	2.92683	
obs.-exp.	-0,70732	2.63415	-1,92683	
Total: observed	24.00000	11.00000	6.00000	41.00000

Depend.: Fasting glucose (mg/dL)	Kruskal-Wallis ANOVA by Ranks; Fasting glucose (mg/dL)<br>Independent (grouping) variable: Hashimoto 1 Hypo-no-Hashimoto 2 control 3<br>Kruskal-Wallis test: H (2, N= 41) =2,968163 p =,2267			
	Code	Valid N	Sum of Ranks	Mean Rank
1	1	24	481.5000	20.06250
2	2	11	207.5000	18.86364
3	3	6	172.0000	28.66667

Dependent: Fasting glucose (mg/dL)	Median Test, Overall Median = 89,0000; Fasting glucose (mg/dL)<br>Independent (grouping) variable: Hashimoto 1 Hypo-no-Hashimoto 2 control 3<br>Chi-Square = ,9003878 df = 2 p = ,6375			
	1	2	3	Total
<= Median: observed	13.00000	6.00000	2.00000	21.00000
expected	12.29268	5.63415	3.07317	
obs.-exp.	0.70732	0.36585	-1,07317	
> Median: observed	11.00000	5.00000	4.00000	20.00000
expected	11.70732	5.36585	2.92683	
obs.-exp.	-0,70732	-0,36585	1.07317	
Total: observed	24.00000	11.00000	6.00000	41.00000

Depend.: Fasting insulin (mIU/L)	Kruskal-Wallis ANOVA by Ranks; Fasting insulin (mIU/L)<br>Independent (grouping) variable: Hashimoto 1 Hypo-no-Hashimoto 2 control 3<br>Kruskal-Wallis test: H(2, N= 41) =,4665433 p =,7919			
	Code	Valid N	Sum of Ranks	Mean Rank
1	1	24	525.5000	21.89583
2	2	11	226.0000	20.54545
3	3	6	109.5000	18.25000

Dependent: Fasting insulin (mIU/L)	Median Test, Overall Median = 9,10000; Fasting insulin (mIU/L)<br>Independent (grouping) variable: Hashimoto 1 Hypo-no-Hashimoto 2 control 3<br>Chi-Square = ,0665584 df = 2 p = ,9673			
	1	2	3	Total
<= Median: observed	12.00000	6.00000	3.000000	21.00000
expected	12.29268	5.63415	3.073171	
obs.-exp.	-0,29268	0.36585	-0,073171	
> Median: observed	12.00000	5.00000	3.000000	20.00000
expected	11.70732	5.36585	2.926829	
obs.-exp.	0.29268	-0,36585	0.073171	
Total: observed	24.00000	11.00000	6.000000	41.00000

Depend.: HOMA-IR	Kruskal-Wallis ANOVA by Ranks; HOMA-IR<br>Independent (grouping) variable: Hashimoto 1 Hypo-no-Hashimoto 2 control 3<br>Kruskal-Wallis test: H (2, N= 41) =,2723497 p =,8727			
	Code	Valid N	Sum of Ranks	Mean Rank
1	1	24	523.0000	21.79167
2	2	11	222.0000	20.18182
3	3	6	116.0000	19.33333

Dependent: HOMA-IR	Median Test, Overall Median = 1,92345; HOMA-IR<br>Independent (grouping) variable: Hashimoto 1 Hypo-no-Hashimoto 2 control 3<br>Chi-Square = ,0665584 df = 2 p = ,9673			
	1	2	3	Total
<= Median: observed	12.00000	6.00000	3.000000	21.00000
expected	12.29268	5.63415	3.073171	
obs.-exp.	-0,29268	0.36585	-0,073171	
> Median: observed	12.00000	5.00000	3.000000	20.00000
expected	11.70732	5.36585	2.926829	
obs.-exp.	0.29268	-0,36585	0.073171	
Total: observed	24.00000	11.00000	6.000000	41.00000

Depend.: Mn [ppb]=[µg/L serum]	Kruskal-Wallis ANOVA by Ranks; Mn [ppb]=[µg/L serum]<br>Independent (grouping) variable: Hashimoto 1 Hypo-no-Hashimoto 2 control 3<br>Kruskal-Wallis test: H(2, N= 41) =,1262802 p =,9388			
	Code	Valid N	Sum of Ranks	Mean Rank
1	1	24	502.0000	20.91667
2	2	11	224.0000	20.36364
3	3	6	135.0000	22.50000

Dependent: Mn [ppb]=[µg/L serum]	Median Test, Overall Median = 2,28666; Mn [ppb]=[µg/L serum]<br>Independent (grouping) variable: Hashimoto 1 Hypo-no-Hashimoto 2 control 3<br>Chi-Square = ,2333243 df = 2 p = ,8899			
	1	2	3	Total
<= Median: observed	13.00000	5.00000	3.000000	21.00000
expected	12.29268	5.63415	3.073171	
obs.-exp.	0.70732	-0,63415	-0,073171	
> Median: observed	11.00000	6.00000	3.000000	20.00000
expected	11.70732	5.36585	2.926829	
obs.-exp.	-0,70732	0.63415	0.073171	
Total: observed	24.00000	11.00000	6.000000	41.00000

Depend.: Se mcg/L	Multiple Comparisons z' values; Se mcg/L<br>Independent (grouping) variable: Hashimoto 1 Hypo-no-Hashimoto 2 control 3<br>Kruskal-Wallis test: H (2, N= 41) =11,82093 p =,0027		
	1<br>R:19,125	2<br>R:16,727	3<br>R:36,333
1		0.549720	3.147266
2	0.549720		3.224868
3	3.147266	3.224868	

Depend.: Se mcg/L	Multiple Comparisons p values (2-tailed); Se mcg/L<br>Independent (grouping) variable: Hashimoto 1 Hypo-no-Hashimoto 2 control 3<br>Kruskal-Wallis test: H (2, N= 41) =11,82093 p =,0027		
	1<br>R:19,125	2<br>R:16,727	3<br>R:36,333
1		1.000000	0.004944
2	1.000000		0.003781
3	0.004944	0.003781	

Depend.: lysoPC a C14:0	Independent (grouping) variable: Hashimoto 1 Hypo-no-Hashimoto 2 control 3<br>Kruskal-Wallis test: H (2, N= 41) =2,564029 p =,2775		
	1<br>R:23,438	2<br>R:16,682	3<br>R:19,167
1		1.548856	0.781101
2	1.548856		0.408716
3	0.781101	0.408716	

Depend.: lysoPC a C14:0	Multiple Comparisons p values (2-tailed); lysoPC a C14:0<br>Independent (grouping) variable: Hashimoto 1 Hypo-no-Hashimoto 2 control 3<br>Kruskal-Wallis test: H (2, N= 41) =2,564029 p =,2775		
	1<br>R:23,438	2<br>R:16,682	3<br>R:19,167
1		0.364249	1.000000
2	0.364249		1.000000
3	1.000000	1.000000	

Depend.: lysoPC a C16:0	Independent (grouping) variable: Hashimoto 1 Hypo-no-Hashimoto 2 control 3<br>Kruskal-Wallis test: H (2, N= 41) =4,717705 p =,0945		
	1<br>R:23,917	2<br>R:14,455	3<br>R:21,333
1		2.169354	0.472471
2	2.169354		1.131445
3	0.472471	1.131445	

Depend.: lysoPC a C16:0	Multiple Comparisons p values (2-tailed); lysoPC a C16:0<br>Independent (grouping) variable: Hashimoto 1 Hypo-no-Hashimoto 2 control 3<br>Kruskal-Wallis test: H (2, N= 41) =4,717705 p =,0945		
	1<br>R:23,917	2<br>R:14,455	3<br>R:21,333
1		0.090167	1.000000
2	0.090167		0.773603
3	1.000000	0.773603	

Depend.: lysoPC a C16:1	Independent (grouping) variable: Hashimoto 1 Hypo-no-Hashimoto 2 control 3<br>Kruskal-Wallis test: H (2, N= 41) =2,918812 p =,2324		
	1<br>R:22,854	2<br>R:15,727	3<br>R:23,250
1		1.633963	0.072395
2	1.633963		1.237362
3	0.072395	1.237362	

Depend.: lysoPC a C16:1	Multiple Comparisons p values (2-tailed); lysoPC a C16:1<br>Independent (grouping) variable: Hashimoto 1 Hypo-no-Hashimoto 2 control 3<br>Kruskal-Wallis test: H (2, N= 41) =2,918812 p =,2324		
	1<br>R:22,854	2<br>R:15,727	3<br>R:23,250
1		0.306800	1.000000
2	0.306800		0.647858
3	1.000000	0.647858	

Depend.: lysoPC a C17:0	Independent (grouping) variable: Hashimoto 1 Hypo-no-Hashimoto 2 control 3<br>Kruskal-Wallis test: H (2, N= 41) =1,625275 p =,4437		
	1<br>R:23,000	2<br>R:17,955	3<br>R:18,583
1		1.156757	0.807773
2	1.156757		0.103425
3	0.807773	0.103425	

Depend.: lysoPC a C17:0	Multiple Comparisons p values (2-tailed); lysoPC a C17:0<br>Independent (grouping) variable: Hashimoto 1 Hypo-no-Hashimoto 2 control 3<br>Kruskal-Wallis test: H (2, N= 41) =1,625275 p =,4437		
	1<br>R:23,000	2<br>R:17,955	3<br>R:18,583
1		0.742114	1.000000
2	0.742114		1.000000
3	1.000000	1.000000	

Depend.: lysoPC a C18:0	Independent (grouping) variable: Hashimoto 1 Hypo-no-Hashimoto 2 control 3<br>Kruskal-Wallis test: H (2, N= 41) =3,782203 p =,1509		
	1<br>R:24,042	2<br>R:16,227	3<br>R:17,583
1		1.791584	1.181177
2	1.791584		0.223049
3	1.181177	0.223049	

Depend.: lysoPC a C18:0	Multiple Comparisons p values (2-tailed); lysoPC a C18:0<br>Independent (grouping) variable: Hashimoto 1 Hypo-no-Hashimoto 2 control 3<br>Kruskal-Wallis test: H (2, N= 41) =3,782203 p =,1509		
	1<br>R:24,042	2<br>R:16,227	3<br>R:17,583
1		0.219599	0.712597
2	0.219599		1.000000
3	0.712597	1.000000	

Depend.: lysoPC a C18:1	Independent (grouping) variable: Hashimoto 1 Hypo-no-Hashimoto 2 control 3<br>Kruskal-Wallis test: H (2, N= 41) =5,245047 p =,0726		
	1<br>R:23,354	2<br>R:13,955	3<br>R:24,500
1		2.155025	0.209564
2	2.155025		1.734550
3	0.209564	1.734550	

Depend.: lysoPC a C18:1	Multiple Comparisons p values (2-tailed); lysoPC a C18:1<br>Independent (grouping) variable: Hashimoto 1 Hypo-no-Hashimoto 2 control 3<br>Kruskal-Wallis test: H (2, N= 41) =5,245047 p =,0726		
	1<br>R:23,354	2<br>R:13,955	3<br>R:24,500
1		0.093480	1.000000
2	0.093480		0.248462
3	1.000000	0.248462	

Depend.: lysoPC a C18:2	Independent (grouping) variable: Hashimoto 1 Hypo-no-Hashimoto 2 control 3<br>Kruskal-Wallis test: H (2, N= 41) =5,742201 p =,0566		
	1<br>R:22,375	2<br>R:14,273	3<br>R:27,833
1		1.857586	0.998285
2	1.857586		2.230492
3	0.998285	2.230492	

Depend.: lysoPC a C18:2	Multiple Comparisons p values (2-tailed); lysoPC a C18:2<br>Independent (grouping) variable: Hashimoto 1 Hypo-no-Hashimoto 2 control 3<br>Kruskal-Wallis test: H (2, N= 41) =5,742201 p =,0566		
	1<br>R:22,375	2<br>R:14,273	3<br>R:27,833
1		0.189684	0.954423
2	0.189684		0.077144
3	0.954423	0.077144	

Depend.: lysoPC a C20:3	Independent (grouping) variable: Hashimoto 1 Hypo-no-Hashimoto 2 control 3<br>Kruskal-Wallis test: H (2, N= 41) =6,675612 p =,0355		
	1<br>R:23,688	2<br>R:13,045	3<br>R:24,833
1		2.439872	0.209564
2	2.439872		1.938908
3	0.209564	1.938908	

Depend.: lysoPC a C20:3	Multiple Comparisons p values (2-tailed); lysoPC a C20:3<br>Independent (grouping) variable: Hashimoto 1 Hypo-no-Hashimoto 2 control 3<br>Kruskal-Wallis test: H (2, N= 41) =6,675612 p =,0355		
	1<br>R:23,688	2<br>R:13,045	3<br>R:24,833
1		0.044077	1.000000
2	0.044077		0.157538
3	1.000000	0.157538	

Depend.: lysoPC a C20:4	Independent (grouping) variable: Hashimoto 1 Hypo-no-Hashimoto 2 control 3<br>Kruskal-Wallis test: H (2, N= 41) =2,722178 p =,2564		
	1<br>R:22,958	2<br>R:15,909	3<br>R:22,500
1		1.616160	0.083825
2	1.616160		1.084094
3	0.083825	1.084094	

Depend.: lysoPC a C20:4	Multiple Comparisons p values (2-tailed); lysoPC a C20:4<br>Independent (grouping) variable: Hashimoto 1 Hypo-no-Hashimoto 2 control 3<br>Kruskal-Wallis test: H (2, N= 41) =2,722178 p =,2564		
	1<br>R:22,958	2<br>R:15,909	3<br>R:22,500
1		0.318179	1.000000
2	0.318179		0.834970
3	1.000000	0.834970	

Depend.: lysoPC a C24:0	Independent (grouping) variable: Hashimoto 1 Hypo-no-Hashimoto 2 control 3<br>Kruskal-Wallis test: H (2, N= 41) =2,188112 p =,3349		
	1<br>R:19,167	2<br>R:25,545	3<br>R:20,000
1		1.462447	0.152410
2	1.462447		0.912134
3	0.152410	0.912134	

Depend.: lysoPC a C24:0	Multiple Comparisons p values (2-tailed); lysoPC a C24:0<br>Independent (grouping) variable: Hashimoto 1 Hypo-no-Hashimoto 2 control 3<br>Kruskal-Wallis test: H (2, N= 41) =2,188112 p =,3349		
	1<br>R:19,167	2<br>R:25,545	3<br>R:20,000
1		0.430856	1.000000
2	0.430856		1.000000
3	1.000000	1.000000	

Depend.: lysoPC a C26:0	Independent (grouping) variable: Hashimoto 1 Hypo-no-Hashimoto 2 control 3<br>Kruskal-Wallis test: H (2, N= 41) =1,321587 p =,5164		
	1<br>R:19,625	2<br>R:24,545	3<br>R:20,000
1		1.128099	0.068584
2	1.128099		0.747651
3	0.068584	0.747651	

Depend.: lysoPC a C26:0	Multiple Comparisons p values (2-tailed); lysoPC a C26:0<br>Independent (grouping) variable: Hashimoto 1 Hypo-no-Hashimoto 2 control 3<br>Kruskal-Wallis test: H (2, N= 41) =1,321587 p =,5164		
	1<br>R:19,625	2<br>R:24,545	3<br>R:20,000
1		0.777834	1.000000
2	0.777834		1.000000
3	1.000000	1.000000	

Depend.: lysoPC a C26:1	Independent (grouping) variable: Hashimoto 1 Hypo-no-Hashimoto 2 control 3<br>Kruskal-Wallis test: H (2, N= 41) =,8012846 p =,6699		
	1<br>R:19,708	2<br>R:23,591	3<br>R:21,417
1		0.890147	0.312440
2	0.890147		0.357626
3	0.312440	0.357626	

Depend.: lysoPC a C26:1	Multiple Comparisons p values (2-tailed); lysoPC a C26:1<br>Independent (grouping) variable: Hashimoto 1 Hypo-no-Hashimoto 2 control 3<br>Kruskal-Wallis test: H (2, N= 41) =,8012846 p =,6699		
	1<br>R:19,708	2<br>R:23,591	3<br>R:21,417
1		1.000000	1.000000
2	1.000000		1.000000
3	1.000000	1.000000	

Depend.: lysoPC a C28:0	Independent (grouping) variable: Hashimoto 1 Hypo-no-Hashimoto 2 control 3<br>Kruskal-Wallis test: H (2, N= 41) =1,084261 p =,5815		
	1<br>R:20,250	2<br>R:24,045	3<br>R:18,417
1		0.870173	0.335302
2	0.870173		0.925841
3	0.335302	0.925841	

Depend.: lysoPC a C28:0	Multiple Comparisons p values (2-tailed); lysoPC a C28:0<br>Independent (grouping) variable: Hashimoto 1 Hypo-no-Hashimoto 2 control 3<br>Kruskal-Wallis test: H (2, N= 41) =1,084261 p =,5815		
	1<br>R:20,250	2<br>R:24,045	3<br>R:18,417
1		1.000000	1.000000
2	1.000000		1.000000
3	1.000000	1.000000	

Depend.: lysoPC a C28:1	Independent (grouping) variable: Hashimoto 1 Hypo-no-Hashimoto 2 control 3<br>Kruskal-Wallis test: H (2, N= 41) =1,360296 p =,5065		
	1<br>R:20,333	2<br>R:24,273	3<br>R:17,667
1		0.903174	0.487712
2	0.903174		1.086586
3	0.487712	1.086586	

Depend.: lysoPC a C28:1	Multiple Comparisons p values (2-tailed); lysoPC a C28:1<br>Independent (grouping) variable: Hashimoto 1 Hypo-no-Hashimoto 2 control 3<br>Kruskal-Wallis test: H (2, N= 41) =1,360296 p =,5065		
	1<br>R:20,333	2<br>R:24,273	3<br>R:17,667
1		1.000000	1.000000
2	1.000000		0.831659
3	1.000000	0.831659	

Depend.: PC aa C24:0	Independent (grouping) variable: Hashimoto 1 Hypo-no-Hashimoto 2 control 3<br>Kruskal-Wallis test: H (2, N= 41) =6,544281 p =,0379		
	1<br>R:23,875	2<br>R:20,545	3<br>R:10,333
1		0.763356	2.476662
2	0.763356		1.679722
3	2.476662	1.679722	

Depend.: PC aa C24:0	Multiple Comparisons p values (2-tailed); PC aa C24:0<br>Independent (grouping) variable: Hashimoto 1 Hypo-no-Hashimoto 2 control 3<br>Kruskal-Wallis test: H (2, N= 41) =6,544281 p =,0379		
	1<br>R:23,875	2<br>R:20,545	3<br>R:10,333
1		1.000000	0.039785
2	1.000000		0.279034
3	0.039785	0.279034	

Depend.: PC aa C26:0	Independent (grouping) variable: Hashimoto 1 Hypo-no-Hashimoto 2 control 3<br>Kruskal-Wallis test: H (2, N= 41) =15,82482 p =,0004		
	1<br>R:25,854	2<br>R:17,955	3<br>R:7,1667
1		1.811124	3.417794
2	1.811124		1.774425
3	3.417794	1.774425	

Depend.: PC aa C26:0	Multiple Comparisons p values (2-tailed); PC aa C26:0<br>Independent (grouping) variable: Hashimoto 1 Hypo-no-Hashimoto 2 control 3<br>Kruskal-Wallis test: H (2, N= 41) =15,82482 p =,0004		
	1<br>R:25,854	2<br>R:17,955	3<br>R:7,1667
1		0.210365	0.001894
2	0.210365		0.227979
3	0.001894	0.227979	

Depend.: PC aa C28:1	Independent (grouping) variable: Hashimoto 1 Hypo-no-Hashimoto 2 control 3<br>Kruskal-Wallis test: H (2, N= 41) =2,799776 p =,2466		
	1<br>R:22,958	2<br>R:20,636	3<br>R:13,833
1		0.532352	1.668889
2	0.532352		1.118984
3	1.668889	1.118984	

Depend.: PC aa C28:1	Multiple Comparisons p values (2-tailed); PC aa C28:1<br>Independent (grouping) variable: Hashimoto 1 Hypo-no-Hashimoto 2 control 3<br>Kruskal-Wallis test: H (2, N= 41) =2,799776 p =,2466		
	1<br>R:22,958	2<br>R:20,636	3<br>R:13,833
1		1.000000	0.285418
2	1.000000		0.789441
3	0.285418	0.789441	

Depend.: PC aa C30:0	Independent (grouping) variable: Hashimoto 1 Hypo-no-Hashimoto 2 control 3<br>Kruskal-Wallis test: H (2, N= 41) =4,849983 p =,0885		
	1<br>R:23,646	2<br>R:20,318	3<br>R:11,667
1		0.762921	2.190894
2	0.762921		1.423029
3	2.190894	1.423029	

Depend.: PC aa C30:0	Multiple Comparisons p values (2-tailed); PC aa C30:0<br>Independent (grouping) variable: Hashimoto 1 Hypo-no-Hashimoto 2 control 3<br>Kruskal-Wallis test: H (2, N= 41) =4,849983 p =,0885		
	1<br>R:23,646	2<br>R:20,318	3<br>R:11,667
1		1.000000	0.085378
2	1.000000		0.464183
3	0.085378	0.464183	

Depend.: PC aa C30:2	[illegible]<br>Independent (grouping) variable: Hashimoto 1 Hypo-no-Hashimoto 2 control 3<br>Kruskal-Wallis test: H (2, N= 41) =0,000000 p =1,000		
	1<br>R:21,000	2<br>R:21,000	3<br>R:21,000
1		0.00	0.00
2	0.00		0.00
3	0.00	0.00	

Depend.: PC aa C30:2	Multiple Comparisons p values (2-tailed); PC aa C30:2<br>Independent (grouping) variable: Hashimoto 1 Hypo-no-Hashimoto 2 control 3<br>Kruskal-Wallis test: H (2, N= 41) =0,000000 p =1,000		
	1<br>R:21,000	2<br>R:21,000	3<br>R:21,000
1		1.000000	1.000000
2	1.000000		1.000000
3	1.000000	1.000000	

Depend.: PC aa C32:0	[illegible]<br>Independent (grouping) variable: Hashimoto 1 Hypo-no-Hashimoto 2 control 3<br>Kruskal-Wallis test: H (2, N= 41) =4,083830 p =,1298		
	1<br>R:23,792	2<br>R:19,136	3<br>R:13,250
1		1.067308	1.927986
2	1.067308		0.968208
3	1.927986	0.968208	

Depend.: PC aa C32:0	Multiple Comparisons p values (2-tailed); PC aa C32:0<br>Independent (grouping) variable: Hashimoto 1 Hypo-no-Hashimoto 2 control 3<br>Kruskal-Wallis test: H (2, N= 41) =4,083830 p =,1298		
	1<br>R:23,792	2<br>R:19,136	3<br>R:13,250
1		0.857498	0.161570
2	0.857498		0.998822
3	0.161570	0.998822	

	Independent (grouping) variable: Hashimoto 1 Hypo-no-Hashimoto 2 control 3<br>Kruskal-Wallis test: H (2, N= 41) =,9301031 p =,6281		
Depend.:<br>PC aa C32:1	1<br>R:22,438	2<br>R:19,636	3<br>R:17,750
1		0.642209	0.857306
2	0.642209		0.310275
3	0.857306	0.310275	

	Multiple Comparisons p values (2-tailed); PC aa C32:1<br>Independent (grouping) variable: Hashimoto 1 Hypo-no-Hashimoto 2 control 3<br>Kruskal-Wallis test: H (2, N= 41) =,9301031 p =,6281		
Depend.:<br>PC aa C32:1	1<br>R:22,438	2<br>R:19,636	3<br>R:17,750
1		1.000000	1.000000
2	1.000000		1.000000
3	1.000000	1.000000	

	Independent (grouping) variable: Hashimoto 1 Hypo-no-Hashimoto 2 control 3<br>Kruskal-Wallis test: H (2, N= 41) =1,985590 p =,3705		
Depend.:<br>PC aa C32:2	1<br>R:23,167	2<br>R:18,591	3<br>R:16,750
1		1.049071	1.173557
2	1.049071		0.302799
3	1.173557	0.302799	

	Multiple Comparisons p values (2-tailed); PC aa C32:2<br>Independent (grouping) variable: Hashimoto 1 Hypo-no-Hashimoto 2 control 3<br>Kruskal-Wallis test: H (2, N= 41) =1,985590 p =,3705		
Depend.:<br>PC aa C32:2	1<br>R:23,167	2<br>R:18,591	3<br>R:16,750
1		0.882436	0.721718
2	0.882436		1.000000
3	0.721718	1.000000	

	Independent (grouping) variable: Hashimoto 1 Hypo-no-Hashimoto 2 control 3<br>Kruskal-Wallis test: H (2, N= 41) =1,406820 p =,4949		
Depend.:<br>PC aa C32:3	1<br>R:22,854	2<br>R:18,682	3<br>R:17,833
1		0.956583	0.918270
2	0.956583		0.139562
3	0.918270	0.139562	

Depend.: PC aa C32:3	Multiple Comparisons p values (2-tailed); PC aa C32:3<br>Independent (grouping) variable: Hashimoto 1 Hypo-no-Hashimoto 2 control 3<br>Kruskal-Wallis test: H (2, N= 41) =1,406820 p =,4949		
	1<br>R:22,854	2<br>R:18,682	3<br>R:17,833
1		1.000000	1.000000
2	1.000000		1.000000
3	1.000000	1.000000	

Depend.: PC aa C34:1	Independent (grouping) variable: Hashimoto 1 Hypo-no-Hashimoto 2 control 3<br>Kruskal-Wallis test: H (2, N= 41) =1,163256 p =,5590		
	1<br>R:22,688	2<br>R:18,364	3<br>R:19,083
1		0.991320	0.659173
2	0.991320		0.118378
3	0.659173	0.118378	

Depend.: PC aa C34:1	Multiple Comparisons p values (2-tailed); PC aa C34:1<br>Independent (grouping) variable: Hashimoto 1 Hypo-no-Hashimoto 2 control 3<br>Kruskal-Wallis test: H (2, N= 41) =1,163256 p =,5590		
	1<br>R:22,688	2<br>R:18,364	3<br>R:19,083
1		0.964588	1.000000
2	0.964588		1.000000
3	1.000000	1.000000	

Depend.: PC aa C34:2	Independent (grouping) variable: Hashimoto 1 Hypo-no-Hashimoto 2 control 3<br>Kruskal-Wallis test: H (2, N= 41) =3,370050 p =,1854		
	1<br>R:23,854	2<br>R:17,591	3<br>R:15,833
1		1.435960	1.466946
2	1.435960		0.289092
3	1.466946	0.289092	

Depend.: PC aa C34:2	Multiple Comparisons p values (2-tailed); PC aa C34:2<br>Independent (grouping) variable: Hashimoto 1 Hypo-no-Hashimoto 2 control 3<br>Kruskal-Wallis test: H (2, N= 41) =3,370050 p =,1854		
	1<br>R:23,854	2<br>R:17,591	3<br>R:15,833
1		0.453041	0.427172
2	0.453041		1.000000
3	0.427172	1.000000	

	Independent (grouping) variable: Hashimoto 1 Hypo-no-Hashimoto 2 control 3<br>Kruskal-Wallis test: H (2, N= 41) =1,430817 p =,4890		
Depend.:<br>PC aa C34:3	1<br>R:22,813	2<br>R:19,136	3<br>R:17,167
1		0.842818	1.032578
2	0.842818		0.323982
3	1.032578	0.323982	

	Multiple Comparisons p values (2-tailed); PC aa C34:3<br>Independent (grouping) variable: Hashimoto 1 Hypo-no-Hashimoto 2 control 3<br>Kruskal-Wallis test: H (2, N= 41) =1,430817 p =,4890		
Depend.:<br>PC aa C34:3	1<br>R:22,813	2<br>R:19,136	3<br>R:17,167
1		1.000000	0.905405
2	1.000000		1.000000
3	0.905405	1.000000	

	Independent (grouping) variable: Hashimoto 1 Hypo-no-Hashimoto 2 control 3<br>Kruskal-Wallis test: H (2, N= 41) =2,941642 p =,2297		
Depend.:<br>PC aa C34:4	1<br>R:23,646	2<br>R:18,000	3<br>R:15,917
1		1.294405	1.413603
2	1.294405		0.342673
3	1.413603	0.342673	

	Multiple Comparisons p values (2-tailed); PC aa C34:4<br>Independent (grouping) variable: Hashimoto 1 Hypo-no-Hashimoto 2 control 3<br>Kruskal-Wallis test: H (2, N= 41) =2,941642 p =,2297		
Depend.:<br>PC aa C34:4	1<br>R:23,646	2<br>R:18,000	3<br>R:15,917
1		0.586577	0.472436
2	0.586577		1.000000
3	0.472436	1.000000	

	Independent (grouping) variable: Hashimoto 1 Hypo-no-Hashimoto 2 control 3<br>Kruskal-Wallis test: H (2, N= 41) =4,692229 p =,0957		
Depend.:<br>PC aa C36:0	1<br>R:23,708	2<br>R:20,000	3<br>R:12,000
1		0.850199	2.141360
2	0.850199		1.315866
3	2.141360	1.315866	

	Multiple Comparisons p values (2-tailed); PC aa C36:0<br>Independent (grouping) variable: Hashimoto 1 Hypo-no-Hashimoto 2 control 3<br>Kruskal-Wallis test: H (2, N= 41) =4,692229 p =,0957		
Depend.:<br>PC aa C36:0	1<br>R:23,708	2<br>R:20,000	3<br>R:12,000
1		1.000000	0.096735
2	1.000000		0.564657
3	0.096735	0.564657	

	Independent (grouping) variable: Hashimoto 1 Hypo-no-Hashimoto 2 control 3<br>Kruskal-Wallis test: H (2, N= 41) =2,468280 p =,2911		
Depend.:<br>PC aa C36:1	1<br>R:23,396	2<br>R:18,455	3<br>R:16,083
1		1.132875	1.337398
2	1.132875		0.390025
3	1.337398	0.390025	

	Multiple Comparisons p values (2-tailed); PC aa C36:1<br>Independent (grouping) variable: Hashimoto 1 Hypo-no-Hashimoto 2 control 3<br>Kruskal-Wallis test: H (2, N= 41) =2,468280 p =,2911		
Depend.:<br>PC aa C36:1	1<br>R:23,396	2<br>R:18,455	3<br>R:16,083
1		0.771800	0.543279
2	0.771800		1.000000
3	0.543279	1.000000	

	Independent (grouping) variable: Hashimoto 1 Hypo-no-Hashimoto 2 control 3<br>Kruskal-Wallis test: H (2, N= 41) =2,805639 p =,2459		
Depend.:<br>PC aa C36:2	1<br>R:23,563	2<br>R:18,227	3<br>R:15,833
1		1.223193	1.413603
2	1.223193		0.393763
3	1.413603	0.393763	

	Multiple Comparisons p values (2-tailed); PC aa C36:2<br>Independent (grouping) variable: Hashimoto 1 Hypo-no-Hashimoto 2 control 3<br>Kruskal-Wallis test: H (2, N= 41) =2,805639 p =,2459		
Depend.:<br>PC aa C36:2	1<br>R:23,563	2<br>R:18,227	3<br>R:15,833
1		0.663771	0.472436
2	0.663771		1.000000
3	0.472436	1.000000	

	Independent (grouping) variable: Hashimoto 1 Hypo-no-Hashimoto 2 control 3<br>Kruskal-Wallis test: H (2, N= 41) =1,187274 p =,5523		
Depend.:<br>PC aa C36:3	1<br>R:22,625	2<br>R:19,455	3<br>R:17,333
1		0.726881	0.967803
2	0.726881		0.348904
3	0.967803	0.348904	

	Multiple Comparisons p values (2-tailed); PC aa C36:3 (Tylko wyniki przyslane w czerwcu 2023)<br>Independent (grouping) variable: Hashimoto 1 Hypo-no-Hashimoto 2 control 3<br>Kruskal-Wallis test: H (2, N= 41) =1,187274 p =,5523		
Depend.:<br>PC aa C36:3	1<br>R:22,625	2<br>R:19,455	3<br>R:17,333
1		1.000000	0.999428
2	1.000000		1.000000
3	0.999428	1.000000	

	Independent (grouping) variable: Hashimoto 1 Hypo-no-Hashimoto 2 control 3<br>Kruskal-Wallis test: H (2, N= 41) =2,891786 p =,2355		
Depend.:<br>PC aa C36:4	1<br>R:23,500	2<br>R:18,773	3<br>R:15,083
1		1.083809	1.539341
2	1.083809		0.606843
3	1.539341	0.606843	

	Multiple Comparisons p values (2-tailed); PC aa C36:4<br>Independent (grouping) variable: Hashimoto 1 Hypo-no-Hashimoto 2 control 3<br>Kruskal-Wallis test: H (2, N= 41) =2,891786 p =,2355		
Depend.:<br>PC aa C36:4	1<br>R:23,500	2<br>R:18,773	3<br>R:15,083
1		0.835349	0.371163
2	0.835349		1.000000
3	0.371163	1.000000	

	Independent (grouping) variable: Hashimoto 1 Hypo-no-Hashimoto 2 control 3<br>Kruskal-Wallis test: H (2, N= 41) =,2505579 p =,8823		
Depend.:<br>PC aa C36:5	1<br>R:20,417	2<br>R:22,545	3<br>R:20,500
1		0.488061	0.015241
2	0.488061		0.336443
3	0.015241	0.336443	

	Multiple Comparisons p values (2-tailed); PC aa C36:5<br>Independent (grouping) variable: Hashimoto 1 Hypo-no-Hashimoto 2 control 3<br>Kruskal-Wallis test: H (2, N= 41) =,2505579 p =,8823		
Depend.:<br>PC aa C36:5	1<br>R:20,417	2<br>R:22,545	3<br>R:20,500
1		1.000000	1.000000
2	1.000000		1.000000
3	1.000000	1.000000	

	Independent (grouping) variable: Hashimoto 1 Hypo-no-Hashimoto 2 control 3<br>Kruskal-Wallis test: H (2, N= 41) =1,206234 p =,5471		
Depend.:<br>PC aa C36:6	1<br>R:22,250	2<br>R:20,864	3<br>R:16,250
1		0.317848	1.097352
2	0.317848		0.758866
3	1.097352	0.758866	

	Multiple Comparisons p values (2-tailed); PC aa C36:6<br>Independent (grouping) variable: Hashimoto 1 Hypo-no-Hashimoto 2 control 3<br>Kruskal-Wallis test: H (2, N= 41) =1,206234 p =,5471		
Depend.:<br>PC aa C36:6	1<br>R:22,250	2<br>R:20,864	3<br>R:16,250
1		1.000000	0.817463
2	1.000000		1.000000
3	0.817463	1.000000	

	Independent (grouping) variable: Hashimoto 1 Hypo-no-Hashimoto 2 control 3<br>Kruskal-Wallis test: H (2, N= 41) =3,748093 p =,1535		
Depend.:<br>PC aa C38:0	1<br>R:23,750	2<br>R:18,909	3<br>R:13,833
1		1.109862	1.813679
2	1.109862		0.834877
3	1.813679	0.834877	

	Multiple Comparisons p values (2-tailed); PC aa C38:0<br>Independent (grouping) variable: Hashimoto 1 Hypo-no-Hashimoto 2 control 3<br>Kruskal-Wallis test: H (2, N= 41) =3,748093 p =,1535		
Depend.:<br>PC aa C38:0	1<br>R:23,750	2<br>R:18,909	3<br>R:13,833
1		0.801176	0.209182
2	0.801176		1.000000
3	0.209182	1.000000	

	Independent (grouping) variable: Hashimoto 1 Hypo-no-Hashimoto 2 control 3<br>Kruskal-Wallis test: H (2, N= 41) =2,671646 p =,2629		
Depend.:<br>PC aa C38:1	1<br>R:20,208	2<br>R:25,455	3<br>R:16,000
1		1.202785	0.769670
2	1.202785		1.555114
3	0.769670	1.555114	

	Multiple Comparisons p values (2-tailed); PC aa C38:1<br>Independent (grouping) variable: Hashimoto 1 Hypo-no-Hashimoto 2 control 3<br>Kruskal-Wallis test: H (2, N= 41) =2,671646 p =,2629		
Depend.:<br>PC aa C38:1	1<br>R:20,208	2<br>R:25,455	3<br>R:16,000
1		0.687179	1.000000
2	0.687179		0.359757
3	1.000000	0.359757	

	Independent (grouping) variable: Hashimoto 1 Hypo-no-Hashimoto 2 control 3<br>Kruskal-Wallis test: H (2, N= 41) =4,919548 p =,0855		
Depend.:<br>PC aa C38:3	1<br>R:24,375	2<br>R:17,455	3<br>R:14,000
1		1.586633	1.897504
2	1.586633		0.568215
3	1.897504	0.568215	

	Multiple Comparisons p values (2-tailed); PC aa C38:3<br>Independent (grouping) variable: Hashimoto 1 Hypo-no-Hashimoto 2 control 3<br>Kruskal-Wallis test: H (2, N= 41) =4,919548 p =,0855		
Depend.:<br>PC aa C38:3	1<br>R:24,375	2<br>R:17,455	3<br>R:14,000
1		0.337787	0.173284
2	0.337787		1.000000
3	0.173284	1.000000	

	Independent (grouping) variable: Hashimoto 1 Hypo-no-Hashimoto 2 control 3<br>Kruskal-Wallis test: H (2, N= 41) =2,932727 p =,2308		
Depend.:<br>PC aa C38:4	1<br>R:23,688	2<br>R:16,909	3<br>R:17,750
1		1.554067	1.085921
2	1.554067		0.138315
3	1.085921	0.138315	

Depend.: PC aa C38:4	Multiple Comparisons p values (2-tailed); PC aa C38:4<br>Independent (grouping) variable: Hashimoto 1 Hypo-no-Hashimoto 2 control 3<br>Kruskal-Wallis test: H (2, N= 41) =2,932727 p =,2308		
	1<br>R:23,688	2<br>R:16,909	3<br>R:17,750
1		0.360505	0.832542
2	0.360505		1.000000
3	0.832542	1.000000	

Depend.: PC aa C38:5	Independent (grouping) variable: Hashimoto 1 Hypo-no-Hashimoto 2 control 3<br>Kruskal-Wallis test: H (2, N= 41) =,5155108 p =,7728		
	1<br>R:22,083	2<br>R:19,909	3<br>R:18,667
1		0.498483	0.624881
2	0.498483		0.204358
3	0.624881	0.204358	

Depend.: PC aa C38:5	Multiple Comparisons p values (2-tailed); PC aa C38:5<br>Independent (grouping) variable: Hashimoto 1 Hypo-no-Hashimoto 2 control 3<br>Kruskal-Wallis test: H (2, N= 41) =,5155108 p =,7728		
	1<br>R:22,083	2<br>R:19,909	3<br>R:18,667
1		1.000000	1.000000
2	1.000000		1.000000
3	1.000000	1.000000	

Depend.: PC aa C38:6	Independent (grouping) variable: Hashimoto 1 Hypo-no-Hashimoto 2 control 3<br>Kruskal-Wallis test: H (2, N= 41) =2,574167 p =,2761		
	1<br>R:21,438	2<br>R:23,773	3<br>R:14,167
1		0.535391	1.329777
2	0.535391		1.580036
3	1.329777	1.580036	

Depend.: PC aa C38:6	Multiple Comparisons p values (2-tailed); PC aa C38:6<br>Independent (grouping) variable: Hashimoto 1 Hypo-no-Hashimoto 2 control 3<br>Kruskal-Wallis test: H (2, N= 41) =2,574167 p =,2761		
	1<br>R:21,438	2<br>R:23,773	3<br>R:14,167
1		1.000000	0.550775
2	1.000000		0.342296
3	0.550775	0.342296	

Depend.: PC aa C40:1	Independent (grouping) variable: Hashimoto 1 Hypo-no-Hashimoto 2 control 3<br>Kruskal-Wallis test: H (2, N= 41) =31,40706 p =,0000		
	1<br>R:24,250	2<br>R:23,455	3<br>R:3,5000
1		0.182372	3.795009
2	0.182372		3.282188
3	3.795009	3.282188	

Depend.: PC aa C40:1	Multiple Comparisons p values (2-tailed); PC aa C40:1<br>Independent (grouping) variable: Hashimoto 1 Hypo-no-Hashimoto 2 control 3<br>Kruskal-Wallis test: H (2, N= 41) =31,40706 p =,0000		
	1<br>R:24,250	2<br>R:23,455	3<br>R:3,5000
1		1.000000	0.000443
2	1.000000		0.003090
3	0.000443	0.003090	

Depend.: PC aa C40:2	Independent (grouping) variable: Hashimoto 1 Hypo-no-Hashimoto 2 control 3<br>Kruskal-Wallis test: H (2, N= 41) =,0892477 p =,9564		
	1<br>R:21,292	2<br>R:21,091	3<br>R:19,667
1		0.046027	0.297199
2	0.046027		0.234264
3	0.297199	0.234264	

Depend.: PC aa C40:2	Multiple Comparisons p values (2-tailed); PC aa C40:2<br>Independent (grouping) variable: Hashimoto 1 Hypo-no-Hashimoto 2 control 3<br>Kruskal-Wallis test: H (2, N= 41) =,0892477 p =,9564		
	1<br>R:21,292	2<br>R:21,091	3<br>R:19,667
1		1.000000	1.000000
2	1.000000		1.000000
3	1.000000	1.000000	

Depend.: PC aa C40:3	Independent (grouping) variable: Hashimoto 1 Hypo-no-Hashimoto 2 control 3<br>Kruskal-Wallis test: H (2, N= 41) =,0612556 p =,9698		
	1<br>R:20,917	2<br>R:21,636	3<br>R:20,167
1		0.165003	0.137169
2	0.165003		0.241740
3	0.137169	0.241740	

	Multiple Comparisons p values (2-tailed); PC aa C40:3<br>Independent (grouping) variable: Hashimoto 1 Hypo-no-Hashimoto 2 control 3<br>Kruskal-Wallis test: H (2, N= 41) =,0612556 p =,9698		
Depend.:<br>PC aa C40:3	1<br>R:20,917	2<br>R:21,636	3<br>R:20,167
1		1.000000	1.000000
2	1.000000		1.000000
3	1.000000	1.000000	

	[illegible]<br>Independent (grouping) variable: Hashimoto 1 Hypo-no-Hashimoto 2 control 3<br>Kruskal-Wallis test: H (2, N= 41) =3,180061 p =,2039		
Depend.:<br>PC aa C40:4	1<br>R:23,792	2<br>R:17,455	3<br>R:16,333
1		1.452894	1.364069
2	1.452894		0.184421
3	1.364069	0.184421	

	Multiple Comparisons p values (2-tailed); PC aa C40:4<br>Independent (grouping) variable: Hashimoto 1 Hypo-no-Hashimoto 2 control 3<br>Kruskal-Wallis test: H (2, N= 41) =3,180061 p =,2039		
Depend.:<br>PC aa C40:4	1<br>R:23,792	2<br>R:17,455	3<br>R:16,333
1		0.438759	0.517637
2	0.438759		1.000000
3	0.517637	1.000000	

	[illegible]<br>Independent (grouping) variable: Hashimoto 1 Hypo-no-Hashimoto 2 control 3<br>Kruskal-Wallis test: H (2, N= 41) =,3172856 p =,8533		
Depend.:<br>PC aa C40:5	1<br>R:21,708	2<br>R:19,273	3<br>R:21,333
1		0.558405	0.068584
2	0.558405		0.338935
3	0.068584	0.338935	

	Multiple Comparisons p values (2-tailed); PC aa C40:5<br>Independent (grouping) variable: Hashimoto 1 Hypo-no-Hashimoto 2 control 3<br>Kruskal-Wallis test: H (2, N= 41) =,3172856 p =,8533		
Depend.:<br>PC aa C40:5	1<br>R:21,708	2<br>R:19,273	3<br>R:21,333
1		1.000000	1.000000
2	1.000000		1.000000
3	1.000000	1.000000	

Depend.: PC aa C40:6	Independent (grouping) variable: Hashimoto 1 Hypo-no-Hashimoto 2 control 3<br>Kruskal-Wallis test: H (2, N= 41) =2,594611 p =,2733		
	1<br>R:21,708	2<br>R:23,318	3<br>R:13,917
1		0.369086	1.425033
2	0.369086		1.546391
3	1.425033	1.546391	

Depend.: PC aa C40:6	Multiple Comparisons p values (2-tailed); PC aa C40:6<br>Independent (grouping) variable: Hashimoto 1 Hypo-no-Hashimoto 2 control 3<br>Kruskal-Wallis test: H (2, N= 41) =2,594611 p =,2733		
	1<br>R:21,708	2<br>R:23,318	3<br>R:13,917
1		1.000000	0.462443
2	1.000000		0.366030
3	0.462443	0.366030	

Depend.: PC aa C42:0	Independent (grouping) variable: Hashimoto 1 Hypo-no-Hashimoto 2 control 3<br>Kruskal-Wallis test: H (2, N= 41) =,2267513 p =,8928		
	1<br>R:20,375	2<br>R:21,318	3<br>R:22,917
1		0.216241	0.464850
2	0.216241		0.262924
3	0.464850	0.262924	

Depend.: PC aa C42:0	Multiple Comparisons p values (2-tailed); PC aa C42:0<br>Independent (grouping) variable: Hashimoto 1 Hypo-no-Hashimoto 2 control 3<br>Kruskal-Wallis test: H (2, N= 41) =,2267513 p =,8928		
	1<br>R:20,375	2<br>R:21,318	3<br>R:22,917
1		1.000000	1.000000
2	1.000000		1.000000
3	1.000000	1.000000	

Depend.: PC aa C42:1	Independent (grouping) variable: Hashimoto 1 Hypo-no-Hashimoto 2 control 3<br>Kruskal-Wallis test: H (2, N= 41) =,2312210 p =,8908		
	1<br>R:20,292	2<br>R:22,364	3<br>R:21,333
1		0.475035	0.190512
2	0.475035		0.169468
3	0.190512	0.169468	

	Multiple Comparisons p values (2-tailed); PC aa C42:1<br>Independent (grouping) variable: Hashimoto 1 Hypo-no-Hashimoto 2 control 3<br>Kruskal-Wallis test: H (2, N= 41) =,2312210 p =,8908		
Depend.:<br>PC aa C42:1	1<br>R:20,292	2<br>R:22,364	3<br>R:21,333
1		1.000000	1.000000
2	1.000000		1.000000
3	1.000000	1.000000	

	Independent (grouping) variable: Hashimoto 1 Hypo-no-Hashimoto 2 control 3<br>Kruskal-Wallis test: H (2, N= 41) =,0107442 p =,9946		
Depend.:<br>PC aa C42:2	1<br>R:20,896	2<br>R:21,318	3<br>R:20,833
1		0.096831	0.011431
2	0.096831		0.079749
3	0.011431	0.079749	

	Multiple Comparisons p values (2-tailed); PC aa C42:2<br>Independent (grouping) variable: Hashimoto 1 Hypo-no-Hashimoto 2 control 3<br>Kruskal-Wallis test: H (2, N= 41) =,0107442 p =,9946		
Depend.:<br>PC aa C42:2	1<br>R:20,896	2<br>R:21,318	3<br>R:20,833
1		1.000000	1.000000
2	1.000000		1.000000
3	1.000000	1.000000	

	Independent (grouping) variable: Hashimoto 1 Hypo-no-Hashimoto 2 control 3<br>Kruskal-Wallis test: H (2, N= 41) =,8079135 p =,6677		
Depend.:<br>PC aa C42:4	1<br>R:21,542	2<br>R:18,455	3<br>R:23,500
1		0.707776	0.358163
2	0.707776		0.829893
3	0.358163	0.829893	

	Multiple Comparisons p values (2-tailed); PC aa C42:4<br>Independent (grouping) variable: Hashimoto 1 Hypo-no-Hashimoto 2 control 3<br>Kruskal-Wallis test: H (2, N= 41) =,8079135 p =,6677		
Depend.:<br>PC aa C42:4	1<br>R:21,542	2<br>R:18,455	3<br>R:23,500
1		1.000000	1.000000
2	1.000000		1.000000
3	1.000000	1.000000	

	Independent (grouping) variable: Hashimoto 1 Hypo-no-Hashimoto 2 control 3<br>Kruskal-Wallis test: H (2, N= 41) =,1114412 p =,9458		
Depend.:<br>PC aa C42:5	1<br>R:20,958	2<br>R:21,773	3<br>R:19,750
1		0.186714	0.220994
2	0.186714		0.332705
3	0.220994	0.332705	

	Multiple Comparisons p values (2-tailed); PC aa C42:5<br>Independent (grouping) variable: Hashimoto 1 Hypo-no-Hashimoto 2 control 3<br>Kruskal-Wallis test: H (2, N= 41) =,1114412 p =,9458		
Depend.:<br>PC aa C42:5	1<br>R:20,958	2<br>R:21,773	3<br>R:19,750
1		1.000000	1.000000
2	1.000000		1.000000
3	1.000000	1.000000	

	Independent (grouping) variable: Hashimoto 1 Hypo-no-Hashimoto 2 control 3<br>Kruskal-Wallis test: H (2, N= 41) =,0231049 p =,9885		
Depend.:<br>PC aa C42:6	1<br>R:21,208	2<br>R:20,545	3<br>R:21,000
1		0.151976	0.038102
2	0.151976		0.074765
3	0.038102	0.074765	

	Multiple Comparisons p values (2-tailed); PC aa C42:6<br>Independent (grouping) variable: Hashimoto 1 Hypo-no-Hashimoto 2 control 3<br>Kruskal-Wallis test: H (2, N= 41) =,0231049 p =,9885		
Depend.:<br>PC aa C42:6	1<br>R:21,208	2<br>R:20,545	3<br>R:21,000
1		1.000000	1.000000
2	1.000000		1.000000
3	1.000000	1.000000	

	Independent (grouping) variable: Hashimoto 1 Hypo-no-Hashimoto 2 control 3<br>Kruskal-Wallis test: H (2, N= 41) =5,768472 p =,0559		
Depend.:<br>PC ae C30:0	1<br>R:24,188	2<br>R:19,273	3<br>R:11,417
1		1.126796	2.335683
2	1.126796		1.292190
3	2.335683	1.292190	

Depend.: PC ae C30:0	Multiple Comparisons p values (2-tailed); PC ae C30:0<br>Independent (grouping) variable: Hashimoto 1 Hypo-no-Hashimoto 2 control 3<br>Kruskal-Wallis test: H (2, N= 41) =5,768472 p =,0559		
	1<br>R:24,188	2<br>R:19,273	3<br>R:11,417
1		0.779486	0.058523
2	0.779486		0.588874
3	0.058523	0.588874	

Depend.: PC ae C30:1	Independent (grouping) variable: Hashimoto 1 Hypo-no-Hashimoto 2 control 3<br>Kruskal-Wallis test: H (2, N= 41) =8,596205 p =,0136		
	1<br>R:24,313	2<br>R:19,818	3<br>R:9,9167
1		1.030400	2.632883
2	1.030400		1.628633
3	2.632883	1.628633	

Depend.: PC ae C30:1	Multiple Comparisons p values (2-tailed); PC ae C30:1<br>Independent (grouping) variable: Hashimoto 1 Hypo-no-Hashimoto 2 control 3<br>Kruskal-Wallis test: H (2, N= 41) =8,596205 p =,0136		
	1<br>R:24,313	2<br>R:19,818	3<br>R:9,9167
1		0.908467	0.025399
2	0.908467		0.310172
3	0.025399	0.310172	

Depend.: PC ae C30:2	Independent (grouping) variable: Hashimoto 1 Hypo-no-Hashimoto 2 control 3<br>Kruskal-Wallis test: H (2, N= 41) =,2798519 p =,8694		
	1<br>R:20,896	2<br>R:22,273	3<br>R:19,083
1		0.315677	0.331492
2	0.315677		0.524602
3	0.331492	0.524602	

Depend.: PC ae C30:2	Multiple Comparisons p values (2-tailed); PC ae C30:2<br>Independent (grouping) variable: Hashimoto 1 Hypo-no-Hashimoto 2 control 3<br>Kruskal-Wallis test: H (2, N= 41) =,2798519 p =,8694		
	1<br>R:20,896	2<br>R:22,273	3<br>R:19,083
1		1.000000	1.000000
2	1.000000		1.000000
3	1.000000	1.000000	

Depend.: PC ae C32:1	Independent (grouping) variable: Hashimoto 1 Hypo-no-Hashimoto 2 control 3<br>Kruskal-Wallis test: H (2, N= 41) =4,827489 p =,0895		
	1<br>R:24,313	2<br>R:17,682	3<br>R:13,833
1		1.520198	1.916556
2	1.520198		0.633011
3	1.916556	0.633011	

Depend.: PC ae C32:1	Multiple Comparisons p values (2-tailed); PC ae C32:1<br>Independent (grouping) variable: Hashimoto 1 Hypo-no-Hashimoto 2 control 3<br>Kruskal-Wallis test: H (2, N= 41) =4,827489 p =,0895		
	1<br>R:24,313	2<br>R:17,682	3<br>R:13,833
1		0.385384	0.165883
2	0.385384		1.000000
3	0.165883	1.000000	

Depend.: PC ae C32:2	Independent (grouping) variable: Hashimoto 1 Hypo-no-Hashimoto 2 control 3<br>Kruskal-Wallis test: H (2, N= 41) =3,919400 p =,1409		
	1<br>R:23,938	2<br>R:18,273	3<br>R:14,250
1		1.298747	1.771766
2	1.298747		0.661671
3	1.771766	0.661671	

Depend.: PC ae C32:2	Multiple Comparisons p values (2-tailed); PC ae C32:2<br>Independent (grouping) variable: Hashimoto 1 Hypo-no-Hashimoto 2 control 3<br>Kruskal-Wallis test: H (2, N= 41) =3,919400 p =,1409		
	1<br>R:23,938	2<br>R:18,273	3<br>R:14,250
1		0.582093	0.229300
2	0.582093		1.000000
3	0.229300	1.000000	

Depend.: PC ae C34:0	Independent (grouping) variable: Hashimoto 1 Hypo-no-Hashimoto 2 control 3<br>Kruskal-Wallis test: H (2, N= 41) =7,436659 p =,0243		
	1<br>R:24,854	2<br>R:18,136	3<br>R:10,833
1		1.540172	2.564298
2	1.540172		1.201226
3	2.564298	1.201226	

	Multiple Comparisons p values (2-tailed); PC ae C34:0<br>Independent (grouping) variable: Hashimoto 1 Hypo-no-Hashimoto 2 control 3<br>Kruskal-Wallis test: H (2, N= 41) =7,436659 p =,0243		
Depend.:<br>PC ae C34:0	1<br>R:24,854	2<br>R:18,136	3<br>R:10,833
1		0.370555	0.031015
2	0.370555		0.688991
3	0.031015	0.688991	

	Independent (grouping) variable: Hashimoto 1 Hypo-no-Hashimoto 2 control 3<br>Kruskal-Wallis test: H (2, N= 41) =2,455415 p =,2930		
Depend.:<br>PC ae C34:1	1<br>R:23,354	2<br>R:18,682	3<br>R:15,833
1		1.071216	1.375500
2	1.071216		0.468528
3	1.375500	0.468528	

	Multiple Comparisons p values (2-tailed); PC ae C34:1<br>Independent (grouping) variable: Hashimoto 1 Hypo-no-Hashimoto 2 control 3<br>Kruskal-Wallis test: H (2, N= 41) =2,455415 p =,2930		
Depend.:<br>PC ae C34:1	1<br>R:23,354	2<br>R:18,682	3<br>R:15,833
1		0.852216	0.506929
2	0.852216		1.000000
3	0.506929	1.000000	

	Independent (grouping) variable: Hashimoto 1 Hypo-no-Hashimoto 2 control 3<br>Kruskal-Wallis test: H (2, N= 41) =4,919781 p =,0854		
Depend.:<br>PC ae C34:2	1<br>R:24,479	2<br>R:16,500	3<br>R:15,333
1		1.829361	1.672700
2	1.829361		0.191897
3	1.672700	0.191897	

	Multiple Comparisons p values (2-tailed); PC ae C34:2<br>Independent (grouping) variable: Hashimoto 1 Hypo-no-Hashimoto 2 control 3<br>Kruskal-Wallis test: H (2, N= 41) =4,919781 p =,0854		
Depend.:<br>PC ae C34:2	1<br>R:24,479	2<br>R:16,500	3<br>R:15,333
1		0.202036	0.283159
2	0.202036		1.000000
3	0.283159	1.000000	

	Independent (grouping) variable: Hashimoto 1 Hypo-no-Hashimoto 2 control 3<br>Kruskal-Wallis test: H (2, N= 41) =3,485126 p =,1751		
Depend.:<br>PC ae C34:3	1<br>R:23,875	2<br>R:17,773	3<br>R:15,417
1		1.399051	1.546961
2	1.399051		0.387532
3	1.546961	0.387532	

	Multiple Comparisons p values (2-tailed); PC ae C34:3<br>Independent (grouping) variable: Hashimoto 1 Hypo-no-Hashimoto 2 control 3<br>Kruskal-Wallis test: H (2, N= 41) =3,485126 p =,1751		
Depend.:<br>PC ae C34:3	1<br>R:23,875	2<br>R:17,773	3<br>R:15,417
1		0.485393	0.365618
2	0.485393		1.000000
3	0.365618	1.000000	

	Independent (grouping) variable: Hashimoto 1 Hypo-no-Hashimoto 2 control 3<br>Kruskal-Wallis test: H (2, N= 41) =,8114601 p =,6665		
Depend.:<br>PC ae C36:0	1<br>R:22,417	2<br>R:18,909	3<br>R:19,167
1		0.804172	0.594399
2	0.804172		0.042367
3	0.594399	0.042367	

	Multiple Comparisons p values (2-tailed); PC ae C36:0<br>Independent (grouping) variable: Hashimoto 1 Hypo-no-Hashimoto 2 control 3<br>Kruskal-Wallis test: H (2, N= 41) =,8114601 p =,6665		
Depend.:<br>PC ae C36:0	1<br>R:22,417	2<br>R:18,909	3<br>R:19,167
1		1.000000	1.000000
2	1.000000		1.000000
3	1.000000	1.000000	

	Independent (grouping) variable: Hashimoto 1 Hypo-no-Hashimoto 2 control 3<br>Kruskal-Wallis test: H (2, N= 41) =2,879686 p =,2370		
Depend.:<br>PC ae C36:1	1<br>R:23,292	2<br>R:19,636	3<br>R:14,333
1		0.838041	1.638407
2	0.838041		0.872259
3	1.638407	0.872259	

Multiple Comparisons p values (2-tailed); PC ae C36:1
Independent (grouping) variable: Hashimoto 1 Hypo-no-Hashimoto 2 control 3
Kruskal-Wallis test: H (2, N= 41) =2,879686 p =,2370

Depend.:<br>PC ae C36:1	1<br>R:23,292	2<br>R:19,636	3<br>R:14,333
1		1.000000	0.304010
2	1.000000		1.000000
3	0.304010	1.000000	

Independent (grouping) variable: Hashimoto 1 Hypo-no-Hashimoto 2 control 3
Kruskal-Wallis test: H (2, N= 41) =3,142384 p =,2078

Depend.:<br>PC ae C36:2	1<br>R:23,688	2<br>R:18,227	3<br>R:15,333
1		1.251851	1.527910
2	1.251851		0.476004
3	1.527910	0.476004	

Multiple Comparisons p values (2-tailed); PC ae C36:2
Independent (grouping) variable: Hashimoto 1 Hypo-no-Hashimoto 2 control 3
Kruskal-Wallis test: H (2, N= 41) =3,142384 p =,2078

Depend.:<br>PC ae C36:2	1<br>R:23,688	2<br>R:18,227	3<br>R:15,333
1		0.631872	0.379605
2	0.631872		1.000000
3	0.379605	1.000000	

Independent (grouping) variable: Hashimoto 1 Hypo-no-Hashimoto 2 control 3
Kruskal-Wallis test: H (2, N= 41) =4,301399 p =,1164

Depend.:<br>PC ae C36:3	1<br>R:24,083	2<br>R:15,182	3<br>R:19,333
1		2.040826	0.868737
2	2.040826		0.682855
3	0.868737	0.682855	

Multiple Comparisons p values (2-tailed); PC ae C36:3
Independent (grouping) variable: Hashimoto 1 Hypo-no-Hashimoto 2 control 3
Kruskal-Wallis test: H (2, N= 41) =4,301399 p =,1164

Depend.:<br>PC ae C36:3	1<br>R:24,083	2<br>R:15,182	3<br>R:19,333
1		0.123804	1.000000
2	0.123804		1.000000
3	1.000000	1.000000	

Depend.: PC ae C36:4	Independent (grouping) variable: Hashimoto 1 Hypo-no-Hashimoto 2 control 3<br>Kruskal-Wallis test: H (2, N= 41) =8,468897 p =,0145		
	1<br>R:25,542	2<br>R:13,773	3<br>R:16,083
1		2.698232	1.729853
2	2.698232		0.380056
3	1.729853	0.380056	

Depend.: PC ae C36:4	Multiple Comparisons p values (2-tailed); PC ae C36:4<br>Independent (grouping) variable: Hashimoto 1 Hypo-no-Hashimoto 2 control 3<br>Kruskal-Wallis test: H (2, N= 41) =8,468897 p =,0145		
	1<br>R:25,542	2<br>R:13,773	3<br>R:16,083
1		0.020913	0.250969
2	0.020913		1.000000
3	0.250969	1.000000	

Depend.: PC ae C36:5	Independent (grouping) variable: Hashimoto 1 Hypo-no-Hashimoto 2 control 3<br>Kruskal-Wallis test: H (2, N= 41) =7,218848 p =,0271		
	1<br>R:25,125	2<br>R:16,455	3<br>R:12,833
1		1.987851	2.248047
2	1.987851		0.595629
3	2.248047	0.595629	

Depend.: PC ae C36:5	Multiple Comparisons p values (2-tailed); PC ae C36:5<br>Independent (grouping) variable: Hashimoto 1 Hypo-no-Hashimoto 2 control 3<br>Kruskal-Wallis test: H (2, N= 41) =7,218848 p =,0271		
	1<br>R:25,125	2<br>R:16,455	3<br>R:12,833
1		0.140484	0.073720
2	0.140484		1.000000
3	0.073720	1.000000	

Depend.: PC ae C38:0	Independent (grouping) variable: Hashimoto 1 Hypo-no-Hashimoto 2 control 3<br>Kruskal-Wallis test: H (2, N= 41) =,4227323 p =,8095		
	1<br>R:21,083	2<br>R:22,273	3<br>R:18,333
1		0.272689	0.502953
2	0.272689		0.647964
3	0.502953	0.647964	

	Multiple Comparisons p values (2-tailed); PC ae C38:0<br>Independent (grouping) variable: Hashimoto 1 Hypo-no-Hashimoto 2 control 3<br>Kruskal-Wallis test: H (2, N= 41) =,4227323 p =,8095		
Depend.:<br>PC ae C38:0	1<br>R:21,083	2<br>R:22,273	3<br>R:18,333
1		1.000000	1.000000
2	1.000000		1.000000
3	1.000000	1.000000	

	Independent (grouping) variable: Hashimoto 1 Hypo-no-Hashimoto 2 control 3<br>Kruskal-Wallis test: H (2, N= 41) =4,803251 p =,0906		
Depend.:<br>PC ae C38:1	1<br>R:20,958	2<br>R:25,727	3<br>R:12,500
1		1.093362	1.546961
2	1.093362		2.175664
3	1.546961	2.175664	

	Multiple Comparisons p values (2-tailed); PC ae C38:1<br>Independent (grouping) variable: Hashimoto 1 Hypo-no-Hashimoto 2 control 3<br>Kruskal-Wallis test: H (2, N= 41) =4,803251 p =,0906		
Depend.:<br>PC ae C38:1	1<br>R:20,958	2<br>R:25,727	3<br>R:12,500
1		0.822705	0.365618
2	0.822705		0.088741
3	0.365618	0.088741	

	Independent (grouping) variable: Hashimoto 1 Hypo-no-Hashimoto 2 control 3<br>Kruskal-Wallis test: H (2, N= 41) =,8617820 p =,6499		
Depend.:<br>PC ae C38:2	1<br>R:22,000	2<br>R:18,136	3<br>R:22,250
1		0.885805	0.045723
2	0.885805		0.676624
3	0.045723	0.676624	

	Multiple Comparisons p values (2-tailed); PC ae C38:2<br>Independent (grouping) variable: Hashimoto 1 Hypo-no-Hashimoto 2 control 3<br>Kruskal-Wallis test: H (2, N= 41) =,8617820 p =,6499		
Depend.:<br>PC ae C38:2	1<br>R:22,000	2<br>R:18,136	3<br>R:22,250
1		1.000000	1.000000
2	1.000000		1.000000
3	1.000000	1.000000	

Depend.: PC ae C38:3	Independent (grouping) variable: Hashimoto 1 Hypo-no-Hashimoto 2 control 3<br>Kruskal-Wallis test: H (2, N= 41) =4,608795 p =,0998		
	1<br>R:23,729	2<br>R:19,864	3<br>R:12,167
1		0.886239	2.114689
2	0.886239		1.266022
3	2.114689	1.266022	

Depend.: PC ae C38:3	Multiple Comparisons p values (2-tailed); PC ae C38:3<br>Independent (grouping) variable: Hashimoto 1 Hypo-no-Hashimoto 2 control 3<br>Kruskal-Wallis test: H (2, N= 41) =4,608795 p =,0998		
	1<br>R:23,729	2<br>R:19,864	3<br>R:12,167
1		1.000000	0.103370
2	1.000000		0.616515
3	0.103370	0.616515	

Depend.: PC ae C38:4	Independent (grouping) variable: Hashimoto 1 Hypo-no-Hashimoto 2 control 3<br>Kruskal-Wallis test: H (2, N= 41) =6,069585 p =,0481		
	1<br>R:24,667	2<br>R:17,545	3<br>R:12,667
1		1.632661	2.194704
2	1.632661		0.802479
3	2.194704	0.802479	

Depend.: PC ae C38:4	Multiple Comparisons p values (2-tailed); PC ae C38:4<br>Independent (grouping) variable: Hashimoto 1 Hypo-no-Hashimoto 2 control 3<br>Kruskal-Wallis test: H (2, N= 41) =6,069585 p =,0481		
	1<br>R:24,667	2<br>R:17,545	3<br>R:12,667
1		0.307621	0.084555
2	0.307621		1.000000
3	0.084555	1.000000	

Depend.: PC ae C38:5	Independent (grouping) variable: Hashimoto 1 Hypo-no-Hashimoto 2 control 3<br>Kruskal-Wallis test: H (2, N= 41) =6,094534 p =,0475		
	1<br>R:24,813	2<br>R:14,591	3<br>R:17,500
1		2.343476	1.337398
2	2.343476		0.478497
3	1.337398	0.478497	

	Multiple Comparisons p values (2-tailed); PC ae C38:5<br>Independent (grouping) variable: Hashimoto 1 Hypo-no-Hashimoto 2 control 3<br>Kruskal-Wallis test: H (2, N= 41) =6,094534 p =,0475		
Depend.:<br>PC ae C38:5	1<br>R:24,813	2<br>R:14,591	3<br>R:17,500
1		0.057315	0.543279
2	0.057315		1.000000
3	0.543279	1.000000	

	Independent (grouping) variable: Hashimoto 1 Hypo-no-Hashimoto 2 control 3<br>Kruskal-Wallis test: H (2, N= 41) =5,543430 p =,0626		
Depend.:<br>PC ae C38:6	1<br>R:24,417	2<br>R:18,136	3<br>R:12,583
1		1.439868	2.164222
2	1.439868		0.913380
3	2.164222	0.913380	

	Multiple Comparisons p values (2-tailed); PC ae C38:6<br>Independent (grouping) variable: Hashimoto 1 Hypo-no-Hashimoto 2 control 3<br>Kruskal-Wallis test: H (2, N= 41) =5,543430 p =,0626		
Depend.:<br>PC ae C38:6	1<br>R:24,417	2<br>R:18,136	3<br>R:12,583
1		0.449714	0.091342
2	0.449714		1.000000
3	0.091342	1.000000	

	Independent (grouping) variable: Hashimoto 1 Hypo-no-Hashimoto 2 control 3<br>Kruskal-Wallis test: H (2, N= 41) =1,042700 p =,5937		
Depend.:<br>PC ae C40:1	1<br>R:22,604	2<br>R:18,591	3<br>R:19,000
1		0.920108	0.659173
2	0.920108		0.067289
3	0.659173	0.067289	

	Multiple Comparisons p values (2-tailed); PC ae C40:1<br>Independent (grouping) variable: Hashimoto 1 Hypo-no-Hashimoto 2 control 3<br>Kruskal-Wallis test: H (2, N= 41) =1,042700 p =,5937		
Depend.:<br>PC ae C40:1	1<br>R:22,604	2<br>R:18,591	3<br>R:19,000
1		1.000000	1.000000
2	1.000000		1.000000
3	1.000000	1.000000	

Depend.: PC ae C40:2	Independent (grouping) variable: Hashimoto 1 Hypo-no-Hashimoto 2 control 3<br>Kruskal-Wallis test: H (2, N= 41) =4,440254 p =,1086		
	1<br>R:23,167	2<br>R:21,364	3<br>R:11,667
1		0.413376	2.103258
2	0.413376		1.594989
3	2.103258	1.594989	

Depend.: PC ae C40:2	Multiple Comparisons p values (2-tailed); PC ae C40:2<br>Independent (grouping) variable: Hashimoto 1 Hypo-no-Hashimoto 2 control 3<br>Kruskal-Wallis test: H (2, N= 41) =4,440254 p =,1086		
	1<br>R:23,167	2<br>R:21,364	3<br>R:11,667
1		1.000000	0.106330
2	1.000000		0.332144
3	0.106330	0.332144	

Depend.: PC ae C40:3	Independent (grouping) variable: Hashimoto 1 Hypo-no-Hashimoto 2 control 3<br>Kruskal-Wallis test: H (2, N= 41) =,6817654 p =,7111		
	1<br>R:22,146	2<br>R:18,545	3<br>R:20,917
1		0.825449	0.224805
2	0.825449		0.390025
3	0.224805	0.390025	

Depend.: PC ae C40:3	Multiple Comparisons p values (2-tailed); PC ae C40:3<br>Independent (grouping) variable: Hashimoto 1 Hypo-no-Hashimoto 2 control 3<br>Kruskal-Wallis test: H (2, N= 41) =,6817654 p =,7111		
	1<br>R:22,146	2<br>R:18,545	3<br>R:20,917
1		1.000000	1.000000
2	1.000000		1.000000
3	1.000000	1.000000	

Depend.: PC ae C40:4	Independent (grouping) variable: Hashimoto 1 Hypo-no-Hashimoto 2 control 3<br>Kruskal-Wallis test: H (2, N= 41) =1,235963 p =,5390		
	1<br>R:22,396	2<br>R:17,591	3<br>R:21,667
1		1.101612	0.133359
2	1.101612		0.670394
3	0.133359	0.670394	

Depend.: PC ae C40:4	Multiple Comparisons p values (2-tailed); PC ae C40:4<br>Independent (grouping) variable: Hashimoto 1 Hypo-no-Hashimoto 2 control 3<br>Kruskal-Wallis test: H (2, N= 41) =1,235963 p =,5390		
	1<br>R:22,396	2<br>R:17,591	3<br>R:21,667
1		0.811892	1.000000
2	0.811892		1.000000
3	1.000000	1.000000	

Depend.: PC ae C40:5	Independent (grouping) variable: Hashimoto 1 Hypo-no-Hashimoto 2 control 3<br>Kruskal-Wallis test: H (2, N= 41) =2,244340 p =,3256		
	1<br>R:23,146	2<br>R:16,636	3<br>R:20,417
1		1.492408	0.499143
2	1.492408		0.621796
3	0.499143	0.621796	

Depend.: PC ae C40:5	Multiple Comparisons p values (2-tailed); PC ae C40:5<br>Independent (grouping) variable: Hashimoto 1 Hypo-no-Hashimoto 2 control 3<br>Kruskal-Wallis test: H (2, N= 41) =2,244340 p =,3256		
	1<br>R:23,146	2<br>R:16,636	3<br>R:20,417
1		0.406777	1.000000
2	0.406777		1.000000
3	1.000000	1.000000	

Depend.: PC ae C40:6	Independent (grouping) variable: Hashimoto 1 Hypo-no-Hashimoto 2 control 3<br>Kruskal-Wallis test: H (2, N= 41) =1,682768 p =,4311		
	1<br>R:22,833	2<br>R:19,636	3<br>R:16,167
1		0.732960	1.219280
2	0.732960		0.570707
3	1.219280	0.570707	

Depend.: PC ae C40:6	Multiple Comparisons p values (2-tailed); PC ae C40:6<br>Independent (grouping) variable: Hashimoto 1 Hypo-no-Hashimoto 2 control 3<br>Kruskal-Wallis test: H (2, N= 41) =1,682768 p =,4311		
	1<br>R:22,833	2<br>R:19,636	3<br>R:16,167
1		1.000000	0.668214
2	1.000000		1.000000
3	0.668214	1.000000	

Depend.: PC ae C42:0	Independent (grouping) variable: Hashimoto 1 Hypo-no-Hashimoto 2 control 3<br>Kruskal-Wallis test: H (2, N= 41) =17,34746 p =,0002		
	1<br>R:24,833	2<br>R:21,636	3<br>R:4,5000
1		0.732960	3.718804
2	0.732960		2.818644
3	3.718804	2.818644	

Depend.: PC ae C42:0	Multiple Comparisons p values (2-tailed); PC ae C42:0<br>Independent (grouping) variable: Hashimoto 1 Hypo-no-Hashimoto 2 control 3<br>Kruskal-Wallis test: H (2, N= 41) =17,34746 p =,0002		
	1<br>R:24,833	2<br>R:21,636	3<br>R:4,5000
1		1.000000	0.000601
2	1.000000		0.014468
3	0.000601	0.014468	

Depend.: PC ae C42:1	Independent (grouping) variable: Hashimoto 1 Hypo-no-Hashimoto 2 control 3<br>Kruskal-Wallis test: H (2, N= 41) =,0908708 p =,9556		
	1<br>R:20,688	2<br>R:20,955	3<br>R:22,333
1		0.061225	0.301010
2	0.061225		0.226787
3	0.301010	0.226787	

Depend.: PC ae C42:1	Multiple Comparisons p values (2-tailed); PC ae C42:1<br>Independent (grouping) variable: Hashimoto 1 Hypo-no-Hashimoto 2 control 3<br>Kruskal-Wallis test: H (2, N= 41) =,0908708 p =,9556		
	1<br>R:20,688	2<br>R:20,955	3<br>R:22,333
1		1.000000	1.000000
2	1.000000		1.000000
3	1.000000	1.000000	

Depend.: PC ae C42:2	Independent (grouping) variable: Hashimoto 1 Hypo-no-Hashimoto 2 control 3<br>Kruskal-Wallis test: H (2, N= 41) =,3718042 p =,8304		
	1<br>R:21,521	2<br>R:21,364	3<br>R:18,250
1		0.036040	0.598209
2	0.036040		0.512141
3	0.598209	0.512141	

Depend.: PC ae C42:2	Multiple Comparisons p values (2-tailed); PC ae C42:2<br>Independent (grouping) variable: Hashimoto 1 Hypo-no-Hashimoto 2 control 3<br>Kruskal-Wallis test: H (2, N= 41) =,3718042 p =,8304		
	1<br>R:21,521	2<br>R:21,364	3<br>R:18,250
1		1.000000	1.000000
2	1.000000		1.000000
3	1.000000	1.000000	

Depend.: PC ae C42:3	Independent (grouping) variable: Hashimoto 1 Hypo-no-Hashimoto 2 control 3<br>Kruskal-Wallis test: H (2, N= 41) =1,456645 p =,4827		
	1<br>R:20,938	2<br>R:18,500	3<br>R:25,833
1		0.558839	0.895409
2	0.558839		1.206210
3	0.895409	1.206210	

Depend.: PC ae C42:3	Multiple Comparisons p values (2-tailed); PC ae C42:3<br>Independent (grouping) variable: Hashimoto 1 Hypo-no-Hashimoto 2 control 3<br>Kruskal-Wallis test: H (2, N= 41) =1,456645 p =,4827		
	1<br>R:20,938	2<br>R:18,500	3<br>R:25,833
1		1.000000	1.000000
2	1.000000		0.683209
3	1.000000	0.683209	

Depend.: PC ae C42:4	Independent (grouping) variable: Hashimoto 1 Hypo-no-Hashimoto 2 control 3<br>Kruskal-Wallis test: H (2, N= 41) =1,718818 p =,4234		
	1<br>R:22,417	2<br>R:16,955	3<br>R:22,750
1		1.252285	0.060964
2	1.252285		0.953255
3	0.060964	0.953255	

Depend.: PC ae C42:4	Multiple Comparisons p values (2-tailed); PC ae C42:4<br>Independent (grouping) variable: Hashimoto 1 Hypo-no-Hashimoto 2 control 3<br>Kruskal-Wallis test: H (2, N= 41) =1,718818 p =,4234		
	1<br>R:22,417	2<br>R:16,955	3<br>R:22,750
1		0.631398	1.000000
2	0.631398		1.000000
3	1.000000	1.000000	

Depend.: PC ae C42:5	Independent (grouping) variable: Hashimoto 1 Hypo-no-Hashimoto 2 control 3<br>Kruskal-Wallis test: H (2, N= 41) =,8297469 p =,6604		
	1<br>R:20,938	2<br>R:19,136	3<br>R:24,667
1		0.412942	0.682035
2	0.412942		0.909642
3	0.682035	0.909642	

Depend.: PC ae C42:5	Multiple Comparisons p values (2-tailed); PC ae C42:5<br>Independent (grouping) variable: Hashimoto 1 Hypo-no-Hashimoto 2 control 3<br>Kruskal-Wallis test: H (2, N= 41) =,8297469 p =,6604		
	1<br>R:20,938	2<br>R:19,136	3<br>R:24,667
1		1.000000	1.000000
2	1.000000		1.000000
3	1.000000	1.000000	

Depend.: PC ae C44:3	Independent (grouping) variable: Hashimoto 1 Hypo-no-Hashimoto 2 control 3<br>Kruskal-Wallis test: H (2, N= 41) =1,219591 p =,5435		
	1<br>R:19,271	2<br>R:23,682	3<br>R:23,000
1		1.011294	0.682035
2	1.011294		0.112148
3	0.682035	0.112148	

Depend.: PC ae C44:3	Multiple Comparisons p values (2-tailed); PC ae C44:3<br>Independent (grouping) variable: Hashimoto 1 Hypo-no-Hashimoto 2 control 3<br>Kruskal-Wallis test: H (2, N= 41) =1,219591 p =,5435		
	1<br>R:19,271	2<br>R:23,682	3<br>R:23,000
1		0.935627	1.000000
2	0.935627		1.000000
3	1.000000	1.000000	

Depend.: PC ae C44:4	Independent (grouping) variable: Hashimoto 1 Hypo-no-Hashimoto 2 control 3<br>Kruskal-Wallis test: H (2, N= 41) =1,563043 p =,4577		
	1<br>R:19,667	2<br>R:20,909	3<br>R:26,500
1		0.284847	1.249762
2	0.284847		0.919611
3	1.249762	0.919611	

	Multiple Comparisons p values (2-tailed); PC ae C44:4<br>Independent (grouping) variable: Hashimoto 1 Hypo-no-Hashimoto 2 control 3<br>Kruskal-Wallis test: H (2, N= 41) =1,563043 p =,4577		
Depend.:<br>PC ae C44:4	1<br>R:19,667	2<br>R:20,909	3<br>R:26,500
1		1.000000	0.634160
2	1.000000		1.000000
3	0.634160	1.000000	

	Independent (grouping) variable: Hashimoto 1 Hypo-no-Hashimoto 2 control 3<br>Kruskal-Wallis test: H (2, N= 41) =1,568241 p =,4565		
Depend.:<br>PC ae C44:5	1<br>R:20,458	2<br>R:19,182	3<br>R:26,500
1		0.292663	1.104972
2	0.292663		1.203718
3	1.104972	1.203718	

	Multiple Comparisons p values (2-tailed); PC ae C44:5<br>Independent (grouping) variable: Hashimoto 1 Hypo-no-Hashimoto 2 control 3<br>Kruskal-Wallis test: H (2, N= 41) =1,568241 p =,4565		
Depend.:<br>PC ae C44:5	1<br>R:20,458	2<br>R:19,182	3<br>R:26,500
1		1.000000	0.807515
2	1.000000		0.686096
3	0.807515	0.686096	

	Independent (grouping) variable: Hashimoto 1 Hypo-no-Hashimoto 2 control 3<br>Kruskal-Wallis test: H (2, N= 41) =1,356185 p =,5076		
Depend.:<br>PC ae C44:6	1<br>R:19,646	2<br>R:21,227	3<br>R:26,000
1		0.362572	1.162126
2	0.362572		0.785033
3	1.162126	0.785033	

	Multiple Comparisons p values (2-tailed); PC ae C44:6<br>Independent (grouping) variable: Hashimoto 1 Hypo-no-Hashimoto 2 control 3<br>Kruskal-Wallis test: H (2, N= 41) =1,356185 p =,5076		
Depend.:<br>PC ae C44:6	1<br>R:19,646	2<br>R:21,227	3<br>R:26,000
1		1.000000	0.735553
2	1.000000		1.000000
3	0.735553	1.000000	

Depend.: SM (OH) C14:1	Independent (grouping) variable: Hashimoto 1 Hypo-no-Hashimoto 2 control 3<br>Kruskal-Wallis test: H (2, N= 41) =3,873297 p =,1442		
	1<br>R:23,250	2<br>R:20,727	3<br>R:12,500
1		0.578379	1.966089
2	0.578379		1.353248
3	1.966089	1.353248	

Depend.: SM (OH) C14:1	Multiple Comparisons p values (2-tailed); SM (OH) C14:1<br>Independent (grouping) variable: Hashimoto 1 Hypo-no-Hashimoto 2 control 3<br>Kruskal-Wallis test: H (2, N= 41) =3,873297 p =,1442		
	1<br>R:23,250	2<br>R:20,727	3<br>R:12,500
1		1.000000	0.147865
2	1.000000		0.527929
3	0.147865	0.527929	

Depend.: SM (OH) C16:1	Independent (grouping) variable: Hashimoto 1 Hypo-no-Hashimoto 2 control 3<br>Kruskal-Wallis test: H (2, N= 41) =4,371151 p =,1124		
	1<br>R:23,625	2<br>R:20,000	3<br>R:12,333
1		0.831094	2.065155
2	0.831094		1.261038
3	2.065155	1.261038	

Depend.: SM (OH) C16:1	Multiple Comparisons p values (2-tailed); SM (OH) C16:1<br>Independent (grouping) variable: Hashimoto 1 Hypo-no-Hashimoto 2 control 3<br>Kruskal-Wallis test: H (2, N= 41) =4,371151 p =,1124		
	1<br>R:23,625	2<br>R:20,000	3<br>R:12,333
1		1.000000	0.116725
2	1.000000		0.621886
3	0.116725	0.621886	

Depend.: SM (OH) C22:1	Independent (grouping) variable: Hashimoto 1 Hypo-no-Hashimoto 2 control 3<br>Kruskal-Wallis test: H (2, N= 41) =1,843577 p =,3978		
	1<br>R:22,417	2<br>R:21,182	3<br>R:15,000
1		0.283110	1.356449
2	0.283110		1.016805
3	1.356449	1.016805	

	Multiple Comparisons p values (2-tailed); SM (OH) C22:1<br>Independent (grouping) variable: Hashimoto 1 Hypo-no-Hashimoto 2 control 3<br>Kruskal-Wallis test: H (2, N= 41) =1,843577 p =,3978		
Depend.:<br>SM (OH) C22:1	1<br>R:22,417	2<br>R:21,182	3<br>R:15,000
1		1.000000	0.524869
2	1.000000		0.927738
3	0.524869	0.927738	

	Independent (grouping) variable: Hashimoto 1 Hypo-no-Hashimoto 2 control 3<br>Kruskal-Wallis test: H (2, N= 41) =4,394038 p =,1111		
Depend.:<br>SM (OH) C22:2	1<br>R:23,438	2<br>R:20,591	3<br>R:12,000
1		0.652630	2.091827
2	0.652630		1.413060
3	2.091827	1.413060	

	Multiple Comparisons p values (2-tailed); SM (OH) C22:2<br>Independent (grouping) variable: Hashimoto 1 Hypo-no-Hashimoto 2 control 3<br>Kruskal-Wallis test: H (2, N= 41) =4,394038 p =,1111		
Depend.:<br>SM (OH) C22:2	1<br>R:23,438	2<br>R:20,591	3<br>R:12,000
1		1.000000	0.109362
2	1.000000		0.472914
3	0.109362	0.472914	

	Independent (grouping) variable: Hashimoto 1 Hypo-no-Hashimoto 2 control 3<br>Kruskal-Wallis test: H (2, N= 41) =,8843730 p =,6426		
Depend.:<br>SM (OH) C24:1	1<br>R:22,479	2<br>R:19,000	3<br>R:18,750
1		0.797659	0.682035
2	0.797659		0.041121
3	0.682035	0.041121	

	Multiple Comparisons p values (2-tailed); SM (OH) C24:1<br>Independent (grouping) variable: Hashimoto 1 Hypo-no-Hashimoto 2 control 3<br>Kruskal-Wallis test: H (2, N= 41) =,8843730 p =,6426		
Depend.:<br>SM (OH) C24:1	1<br>R:22,479	2<br>R:19,000	3<br>R:18,750
1		1.000000	1.000000
2	1.000000		1.000000
3	1.000000	1.000000	

Depend.: SM C16:0	Multiple Comparisons z' values; SM C16:0<br>Independent (grouping) variable: Hashimoto 1 Hypo-no-Hashimoto 2 control 3<br>Kruskal-Wallis test: H (2, N= 41) =4,124887 p =,1271		
	1<br>R:24,000	2<br>R:18,273	3<br>R:14,000
1		1.313076	1.828920
2	1.313076		0.702792
3	1.828920	0.702792	

Depend.: SM C16:0	Multiple Comparisons p values (2-tailed); SM C16:0<br>Independent (grouping) variable: Hashimoto 1 Hypo-no-Hashimoto 2 control 3<br>Kruskal-Wallis test: H (2, N= 41) =4,124887 p =,1271		
	1<br>R:24,000	2<br>R:18,273	3<br>R:14,000
1		0.567472	0.202235
2	0.567472		1.000000
3	0.202235	1.000000	

Depend.: SM C16:1	Multiple Comparisons z' values; SM C16:1<br>Independent (grouping) variable: Hashimoto 1 Hypo-no-Hashimoto 2 control 3<br>Kruskal-Wallis test: H (2, N= 41) =2,679622 p =,2619		
	1<br>R:22,979	2<br>R:20,455	3<br>R:14,083
1		0.578813	1.626977
2	0.578813		1.047957
3	1.626977	1.047957	

Depend.: SM C16:1	Multiple Comparisons p values (2-tailed); SM C16:1<br>Independent (grouping) variable: Hashimoto 1 Hypo-no-Hashimoto 2 control 3<br>Kruskal-Wallis test: H (2, N= 41) =2,679622 p =,2619		
	1<br>R:22,979	2<br>R:20,455	3<br>R:14,083
1		1.000000	0.311226
2	1.000000		0.883975
3	0.311226	0.883975	

Depend.: SM C18:0	Multiple Comparisons z' values; SM C18:0<br>Independent (grouping) variable: Hashimoto 1 Hypo-no-Hashimoto 2 control 3<br>Kruskal-Wallis test: H (2, N= 41) =5,723040 p =,0572		
	1<br>R:23,958	2<br>R:20,000	3<br>R:11,000
1		0.907516	2.369975
2	0.907516		1.480349
3	2.369975	1.480349	

	Multiple Comparisons p values (2-tailed); SM C18:0<br>Independent (grouping) variable: Hashimoto 1 Hypo-no-Hashimoto 2 control 3<br>Kruskal-Wallis test: H (2, N= 41) =5,723040 p =,0572		
Depend.:<br>SM C18:0	1<br>R:23,958	2<br>R:20,000	3<br>R:11,000
1		1.000000	0.053368
2	1.000000		0.416341
3	0.053368	0.416341	

	Multiple Comparisons z' values; SM C18:1<br>Independent (grouping) variable: Hashimoto 1 Hypo-no-Hashimoto 2 control 3<br>Kruskal-Wallis test: H (2, N= 41) =5,970296 p =,0505		
Depend.:<br>SM C18:1	1<br>R:24,208	2<br>R:19,364	3<br>R:11,167
1		1.110730	2.385216
2	1.110730		1.348264
3	2.385216	1.348264	

	Multiple Comparisons p values (2-tailed); SM C18:1<br>Independent (grouping) variable: Hashimoto 1 Hypo-no-Hashimoto 2 control 3<br>Kruskal-Wallis test: H (2, N= 41) =5,970296 p =,0505		
Depend.:<br>SM C18:1	1<br>R:24,208	2<br>R:19,364	3<br>R:11,167
1		0.800053	0.051207
2	0.800053		0.532721
3	0.051207	0.532721	

	Multiple Comparisons z' values; SM C20:2<br>Independent (grouping) variable: Hashimoto 1 Hypo-no-Hashimoto 2 control 3<br>Kruskal-Wallis test: H (2, N= 41) =,6545346 p =,7209		
Depend.:<br>SM C20:2	1<br>R:21,396	2<br>R:22,091	3<br>R:17,417
1		0.159358	0.727758
2	0.159358		0.768834
3	0.727758	0.768834	

	Multiple Comparisons p values (2-tailed); SM C20:2<br>Independent (grouping) variable: Hashimoto 1 Hypo-no-Hashimoto 2 control 3<br>Kruskal-Wallis test: H (2, N= 41) =,6545346 p =,7209		
Depend.:<br>SM C20:2	1<br>R:21,396	2<br>R:22,091	3<br>R:17,417
1		1.000000	1.000000
2	1.000000		1.000000
3	1.000000	1.000000	

	Multiple Comparisons z' values; SM C22:3<br>Independent (grouping) variable: Hashimoto 1 Hypo-no-Hashimoto 2 control 3<br>Kruskal-Wallis test: H (2, N= 41) =0,000000 p =1,000		
Depend.:<br>SM C22:3	1<br>R:21,000	2<br>R:21,000	3<br>R:21,000
1		0.00	0.00
2	0.00		0.00
3	0.00	0.00	

	Multiple Comparisons p values (2-tailed); SM C22:3<br>Independent (grouping) variable: Hashimoto 1 Hypo-no-Hashimoto 2 control 3<br>Kruskal-Wallis test: H (2, N= 41) =0,000000 p =1,000		
Depend.:<br>SM C22:3	1<br>R:21,000	2<br>R:21,000	3<br>R:21,000
1		1.000000	1.000000
2	1.000000		1.000000
3	1.000000	1.000000	

	Multiple Comparisons z' values; SM C24:0<br>Independent (grouping) variable: Hashimoto 1 Hypo-no-Hashimoto 2 control 3<br>Kruskal-Wallis test: H (2, N= 41) =,2834391 p =,8679		
Depend.:<br>SM C24:0	1<br>R:21,542	2<br>R:19,364	3<br>R:21,833
1		0.499351	0.053343
2	0.499351		0.406224
3	0.053343	0.406224	

	Multiple Comparisons p values (2-tailed); SM C24:0<br>Independent (grouping) variable: Hashimoto 1 Hypo-no-Hashimoto 2 control 3<br>Kruskal-Wallis test: H (2, N= 41) =,2834391 p =,8679		
Depend.:<br>SM C24:0	1<br>R:21,542	2<br>R:19,364	3<br>R:21,833
1		1.000000	1.000000
2	1.000000		1.000000
3	1.000000	1.000000	

	Multiple Comparisons z' values; SM C24:1<br>Independent (grouping) variable: Hashimoto 1 Hypo-no-Hashimoto 2 control 3<br>Kruskal-Wallis test: H (2, N= 41) =2,744651 p =,2535		
Depend.:<br>SM C24:1	1<br>R:23,208	2<br>R:19,773	3<br>R:14,417
1		0.787672	1.607925
2	0.787672		0.880982
3	1.607925	0.880982	

	Multiple Comparisons p values (2-tailed); SM C24:1<br>Independent (grouping) variable: Hashimoto 1 Hypo-no-Hashimoto 2 control 3<br>Kruskal-Wallis test: H (2, N= 41) =2,744651 p =,2535		
Depend.:<br>SM C24:1	1<br>R:23,208	2<br>R:19,773	3<br>R:14,417
1		1.000000	0.323555
2	1.000000		1.000000
3	0.323555	1.000000	

	Multiple Comparisons z' values; SM C26:0<br>Independent (grouping) variable: Hashimoto 1 Hypo-no-Hashimoto 2 control 3<br>Kruskal-Wallis test: H (2, N= 41) =,1326104 p =,9358		
Depend.:<br>SM C26:0	1<br>R:21,521	2<br>R:20,591	3<br>R:19,667
1		0.213201	0.339112
2	0.213201		0.152022
3	0.339112	0.152022	

	Multiple Comparisons p values (2-tailed); SM C26:0<br>Independent (grouping) variable: Hashimoto 1 Hypo-no-Hashimoto 2 control 3<br>Kruskal-Wallis test: H (2, N= 41) =,1326104 p =,9358		
Depend.:<br>SM C26:0	1<br>R:21,521	2<br>R:20,591	3<br>R:19,667
1		1.000000	1.000000
2	1.000000		1.000000
3	1.000000	1.000000	

	Multiple Comparisons z' values; SM C26:1<br>Independent (grouping) variable: Hashimoto 1 Hypo-no-Hashimoto 2 control 3<br>Kruskal-Wallis test: H (2, N= 41) =,8068047 p =,6680		
Depend.:<br>SM C26:1	1<br>R:22,250	2<br>R:20,136	3<br>R:17,583
1		0.484588	0.853496
2	0.484588		0.419931
3	0.853496	0.419931	

	Multiple Comparisons p values (2-tailed); SM C26:1<br>Independent (grouping) variable: Hashimoto 1 Hypo-no-Hashimoto 2 control 3<br>Kruskal-Wallis test: H (2, N= 41) =,8068047 p =,6680		
Depend.:<br>SM C26:1	1<br>R:22,250	2<br>R:20,136	3<br>R:17,583
1		1.000000	1.000000
2	1.000000		1.000000
3	1.000000	1.000000	

Depend.: Hexoses	Multiple Comparisons z' values; Hexoses<br>Independent (grouping) variable: Hashimoto 1 Hypo-no-Hashimoto 2 control 3<br>Kruskal-Wallis test: H (2, N= 41) =1,827130 p =,4011		
	1<br>R:22,375	2<br>R:21,273	3<br>R:15,000
1		0.252715	1.348828
2	0.252715		1.031758
3	1.348828	1.031758	

Depend.: Hexoses	Multiple Comparisons p values (2-tailed); Hexoses<br>Independent (grouping) variable: Hashimoto 1 Hypo-no-Hashimoto 2 control 3<br>Kruskal-Wallis test: H (2, N= 41) =1,827130 p =,4011		
	1<br>R:22,375	2<br>R:21,273	3<br>R:15,000
1		1.000000	0.532176
2	1.000000		0.906556
3	0.532176	0.906556	

Depend.: Age (years)	Multiple Comparisons z' values; Age (years)<br>Independent (grouping) variable: Hashimoto 1 Hypo-no-Hashimoto 2 control 3<br>Kruskal-Wallis test: H (2, N= 41) =2,001866 p =,3675		
	1<br>R:23,188	2<br>R:18,273	3<br>R:17,250
1		1.126796	1.085921
2	1.126796		0.168221
3	1.085921	0.168221	

Depend.: Age (years)	Multiple Comparisons p values (2-tailed); Age (years)<br>Independent (grouping) variable: Hashimoto 1 Hypo-no-Hashimoto 2 control 3<br>Kruskal-Wallis test: H (2, N= 41) =2,001866 p =,3675		
	1<br>R:23,188	2<br>R:18,273	3<br>R:17,250
1		0.779486	0.832542
2	0.779486		1.000000
3	0.832542	1.000000	

Depend.: BMI (kg/m2)	Multiple Comparisons z' values; BMI (kg/m2)<br>Independent (grouping) variable: Hashimoto 1 Hypo-no-Hashimoto 2 control 3<br>Kruskal-Wallis test: H (2, N= 41) =1,394230 p =,4980		
	1<br>R:21,958	2<br>R:21,818	3<br>R:15,667
1		0.032132	1.150695
2	0.032132		1.011821
3	1.150695	1.011821	

Depend.: BMI (kg/m2)	Multiple Comparisons p values (2-tailed); BMI (kg/m2)<br>Independent (grouping) variable: Hashimoto 1 Hypo-no-Hashimoto 2 control 3<br>Kruskal-Wallis test: H (2, N= 41) =1,394230 p =,4980		
	1<br>R:21,958	2<br>R:21,818	3<br>R:15,667
1		1.000000	0.749573
2	1.000000		0.934871
3	0.749573	0.934871	

Depend.: Hypothyroidism (years)	Multiple Comparisons z' values; Hypothyroidism (years)<br>Independent (grouping) variable: Hashimoto 1 Hypo-no-Hashimoto 2 control 3<br>Kruskal-Wallis test: H(2, N= 35) =0,000000 p =1,000		
	1<br>R:19,729	2<br>R:14,227	3<br>R:--
1		1.474638	
2	1.474638		
3			

Depend.: Hypothyroidism (years)	Multiple Comparisons p values (2-tailed); Hypothyroidism (years)<br>Independent (grouping) variable: Hashimoto 1 Hypo-no-Hashimoto 2 control 3<br>Kruskal-Wallis test: H (2, N= 35) =0,000000 p =1,000		
	1<br>R:19,729	2<br>R:14,227	3<br>R:--
1		0.420929	
2	0.420929		
3			

Depend.: IT4 dose	Multiple Comparisons z' values; IT4 dose<br>Independent (grouping) variable: Hashimoto 1 Hypo-no-Hashimoto 2 control 3<br>Kruskal-Wallis test: H (2, N= 35) =0,000000 p =1,000		
	1<br>R:21,313	2<br>R:10,773	3<br>R:--
1		2.824909	
2	2.824909		
3			

Depend.: IT4 dose	Multiple Comparisons p values (2-tailed); IT4 dose<br>Independent (grouping) variable: Hashimoto 1 Hypo-no-Hashimoto 2 control 3<br>Kruskal-Wallis test: H (2, N= 35) =0,000000 p =1,000		
	1<br>R:21,313	2<br>R:10,773	3<br>R:--
1<br>2	0.014188	0.014188	
3			

Depend.: TSH (uIU/mL)	Multiple Comparisons z' values; TSH (uIU/mL)<br>Independent (grouping) variable: Hashimoto 1 Hypo-no-Hashimoto 2 control 3<br>Kruskal-Wallis test: H (2, N= 41) =2,951331 p =,2286		
	1<br>R:23,625	2<br>R:18,182	3<br>R:15,667
1		1.247943	1.455515
2	1.247943		0.413700
3	1.455515	0.413700	

Depend.: TSH (uIU/mL)	Multiple Comparisons p values (2-tailed); TSH (uIU/mL)<br>Independent (grouping) variable: Hashimoto 1 Hypo-no-Hashimoto 2 control 3<br>Kruskal-Wallis test: H (2, N= 41) =2,951331 p =,2286		
	1<br>R:23,625	2<br>R:18,182	3<br>R:15,667
1		0.636156	0.436580
2	0.636156		1.000000
3	0.436580	1.000000	

Depend.: fT4 (ng/dL)	Multiple Comparisons z' values; fT4 (ng/dL)<br>Independent (grouping) variable: Hashimoto 1 Hypo-no-Hashimoto 2 control 3<br>Kruskal-Wallis test: H (2, N= 41) =3,308598 p =,1912		
	1<br>R:18,146	2<br>R:24,727	3<br>R:25,583
1		1.508908	1.360259
2	1.508908		0.140808
3	1.360259	0.140808	

Depend.: fT4 (ng/dL)	Multiple Comparisons p values (2-tailed); fT4 (ng/dL)<br>Independent (grouping) variable: Hashimoto 1 Hypo-no-Hashimoto 2 control 3<br>Kruskal-Wallis test: H (2, N= 41) =3,308598 p =,1912		
	1<br>R:18,146	2<br>R:24,727	3<br>R:25,583
1		0.393967	0.521244
2	0.393967		1.000000
3	0.521244	1.000000	

Depend.: fT3 (pg/mL)	Multiple Comparisons z' values; fT3 (pg/mL)<br>Independent (grouping) variable: Hashimoto 1 Hypo-no-Hashimoto 2 control 3<br>Kruskal-Wallis test: H (2, N= 41) =1,628421 p =,4430		
	1<br>R:19,104	2<br>R:22,773	3<br>R:25,333
1		0.841081	1.139265
2	0.841081		0.421177
3	1.139265	0.421177	

Depend.: fT3 (pg/mL)	Independent (grouping) variable: Hashimoto 1 Hypo-no-Hashimoto 2 control 3<br>Kruskal-Wallis test: H (2, N= 41) =1,628421 p =,4430		
	1<br>R:19,104	2<br>R:22,773	3<br>R:25,333
1		1.000000	0.763778
2	1.000000		1.000000
3	0.763778	1.000000	

Depend.: anty-TPO levels above the upper limit	Multiple Comparisons z' values; anty-TPO levels above the upper limit<br>Independent (grouping) variable: Hashimoto 1 Hypo-no-Hashimoto 2 control 3<br>Kruskal-Wallis test: H (2, N= 24) =0,000000 p =1,000		
	1<br>R:12,500	2<br>R:--	3<br>R:--
1<br>2			
3			

Depend.: anty-TPO levels above the upper limit	Multiple Comparisons p values (2-tailed); anty-TPO levels above the upper limit<br>Independent (grouping) variable: Hashimoto 1 Hypo-no-Hashimoto 2 control 3<br>Kruskal-Wallis test: H (2, N= 24) =0,000000 p =1,000		
	1<br>R:12,500	2<br>R:--	3<br>R:--
1<br>2			
3			

Depend.: Total cholesterol (mg/dL)	Multiple Comparisons z' values; Total cholesterol (mg/dL)<br>Independent (grouping) variable: Hashimoto 1 Hypo-no-Hashimoto 2 control 3<br>Kruskal-Wallis test: H (2, N= 41) =4,105231 p =,1284		
	1<br>R:23,938	2<br>R:18,545	3<br>R:13,750
1		1.236219	1.863212
2	1.236219		0.788772
3	1.863212	0.788772	

Depend.: Total cholesterol (mg/dL)	Multiple Comparisons p values (2-tailed); Total cholesterol (mg/dL)<br>Independent (grouping) variable: Hashimoto 1 Hypo-no-Hashimoto 2 control 3<br>Kruskal-Wallis test: H (2, N= 41) =4,105231 p =,1284		
	1<br>R:23,938	2<br>R:18,545	3<br>R:13,750
1		0.649131	0.187297
2	0.649131		1.000000
3	0.187297	1.000000	

	Multiple Comparisons z' values; HDL cholesterol (mg/dL)<br>Independent (grouping) variable: Hashimoto 1 Hypo-no-Hashimoto 2 control 3<br>Kruskal-Wallis test: H (2, N= 41) =4,820234 p =,0898		
Depend.:<br>HDL cholesterol (mg/dL)	1<br>R:24,125	2<br>R:18,591	3<br>R:12,917
1		1.268786	2.049914
2	1.268786		0.933318
3	2.049914	0.933318	

	Multiple Comparisons p values (2-tailed); HDL cholesterol (mg/dL)<br>Independent (grouping) variable: Hashimoto 1 Hypo-no-Hashimoto 2 control 3<br>Kruskal-Wallis test: H (2, N= 41) =4,820234 p =,0898		
Depend.:<br>HDL cholesterol (mg/dL)	1<br>R:24,125	2<br>R:18,591	3<br>R:12,917
1		0.613553	0.121118
2	0.613553		1.000000
3	0.121118	1.000000	

	Multiple Comparisons z' values; LDL cholesterol (mg/dL)<br>Independent (grouping) variable: Hashimoto 1 Hypo-no-Hashimoto 2 control 3<br>Kruskal-Wallis test: H (2, N= 41) =,1102600 p =,9464		
Depend.:<br>LDL cholesterol (mg/dL)	1<br>R:21,250	2<br>R:21,273	3<br>R:19,500
1		0.005211	0.320061
2	0.005211		0.291584
3	0.320061	0.291584	

	Multiple Comparisons p values (2-tailed); LDL cholesterol (mg/dL)<br>Independent (grouping) variable: Hashimoto 1 Hypo-no-Hashimoto 2 control 3<br>Kruskal-Wallis test: H (2, N= 41) =,1102600 p =,9464		
Depend.:<br>LDL cholesterol (mg/dL)	1<br>R:21,250	2<br>R:21,273	3<br>R:19,500
1		1.000000	1.000000
2	1.000000		1.000000
3	1.000000	1.000000	

	Multiple Comparisons z' values; Triglycerides<br>Independent (grouping) variable: Hashimoto 1 Hypo-no-Hashimoto 2 control 3<br>Kruskal-Wallis test: H (2, N= 41) =3,684938 p =,1584		
Depend.:<br>Triglycerides	1<br>R:22,563	2<br>R:22,318	3<br>R:12,333
1		0.056014	1.870833
2	0.056014		1.642340
3	1.870833	1.642340	

	Multiple Comparisons p values (2-tailed); Triglycerides<br>Independent (grouping) variable: Hashimoto 1 Hypo-no-Hashimoto 2 control 3<br>Kruskal-Wallis test: H (2, N= 41) =3,684938 p =,1584		
Depend.:<br>Triglycerides	1<br>R:22,563	2<br>R:22,318	3<br>R:12,333
1		1.000000	0.184105
2	1.000000		0.301559
3	0.184105	0.301559	

	Independent (grouping) variable: Hashimoto 1 Hypo-no-Hashimoto 2 control 3<br>Kruskal-Wallis test: H (2, N= 41) =2,731200 p =,2552		
Depend.:<br>AIP	1<br>R:20,729	2<br>R:24,909	3<br>R:14,917
1		0.958320	1.063060
2	0.958320		1.643586
3	1.063060	1.643586	

	Multiple Comparisons p values (2-tailed); AIP<br>Independent (grouping) variable: Hashimoto 1 Hypo-no-Hashimoto 2 control 3<br>Kruskal-Wallis test: H (2, N= 41) =2,731200 p =,2552		
Depend.:<br>AIP	1<br>R:20,729	2<br>R:24,909	3<br>R:14,917
1		1.000000	0.863265
2	1.000000		0.300785
3	0.863265	0.300785	

	Multiple Comparisons z' values; Fasting glucose (mg/dL)<br>Independent (grouping) variable: Hashimoto 1 Hypo-no-Hashimoto 2 control 3<br>Kruskal-Wallis test: H (2, N= 41) =2,968163 p =,2267		
Depend.:<br>Fasting glucose (mg/dL)	1<br>R:20,063	2<br>R:18,864	3<br>R:28,667
1		0.274860	1.573633
2	0.274860		1.612434
3	1.573633	1.612434	

	Multiple Comparisons p values (2-tailed); Fasting glucose (mg/dL)<br>Independent (grouping) variable: Hashimoto 1 Hypo-no-Hashimoto 2 control 3<br>Kruskal-Wallis test: H (2, N= 41) =2,968163 p =,2267		
Depend.:<br>Fasting glucose (mg/dL)	1<br>R:20,063	2<br>R:18,864	3<br>R:28,667
1		1.000000	0.346717
2	1.000000		0.320603
3	0.346717	0.320603	

Depend.: Fasting insulin (mg/dL)	Multiple Comparisons z' values; Fastig insulin (mIU/L)<br>Independent (grouping) variable: Hashimoto 1 Hypo-no-Hashimoto 2 control 3<br>Kruskal-Wallis test: H (2, N= 41) =,4665433 p =,7919		
	1<br>R:21,896	2<br>R:20,545	3<br>R:18,250
1		0.309598	0.666794
2	0.309598		0.377564
3	0.666794	0.377564	

Depend.: Fasting insulin (mIU/L)	Multiple Comparisons p values (2-tailed); Fasting insulin (mIU/L)<br>Independent (grouping) variable: Hashimoto 1 Hypo-no-Hashimoto 2 control 3<br>Kruskal-Wallis test: H (2, N= 41) =,4665433 p =,7919		
	1<br>R:21,896	2<br>R:20,545	3<br>R:18,250
1		1.000000	1.000000
2	1.000000		1.000000
3	1.000000	1.000000	

Depend.: HOMA-IR	Multiple Comparisons z' values; HOMA-IR<br>Independent (grouping) variable: Hashimoto 1 Hypo-no-Hashimoto 2 control 3<br>Kruskal-Wallis test: H (2, N= 41) =,2723497 p =,8727		
	1<br>R:21,792	2<br>R:20,182	3<br>R:19,333
1		0.369086	0.449609
2	0.369086		0.139562
3	0.449609	0.139562	

Depend.: HOMA-IR	Multiple Comparisons p values (2-tailed); HOMA-IR<br>Independent (grouping) variable: Hashimoto 1 Hypo-no-Hashimoto 2 control 3<br>Kruskal-Wallis test: H (2, N= 41) =,2723497 p =,8727		
	1<br>R:21,792	2<br>R:20,182	3<br>R:19,333
1		1.000000	1.000000
2	1.000000		1.000000
3	1.000000	1.000000	
